# Supplementary figures and images for: Reducing the effective dosage of flutamide on prostate cancer cell lines through combination with selenium nanoparticles: An in-vitro study
Source: PLoS One. 2025 May 19;20(5):e0318483. doi: 10.1371/journal.pone.0318483 (PMC12088047; doi:10.1371/journal.pone.0318483)

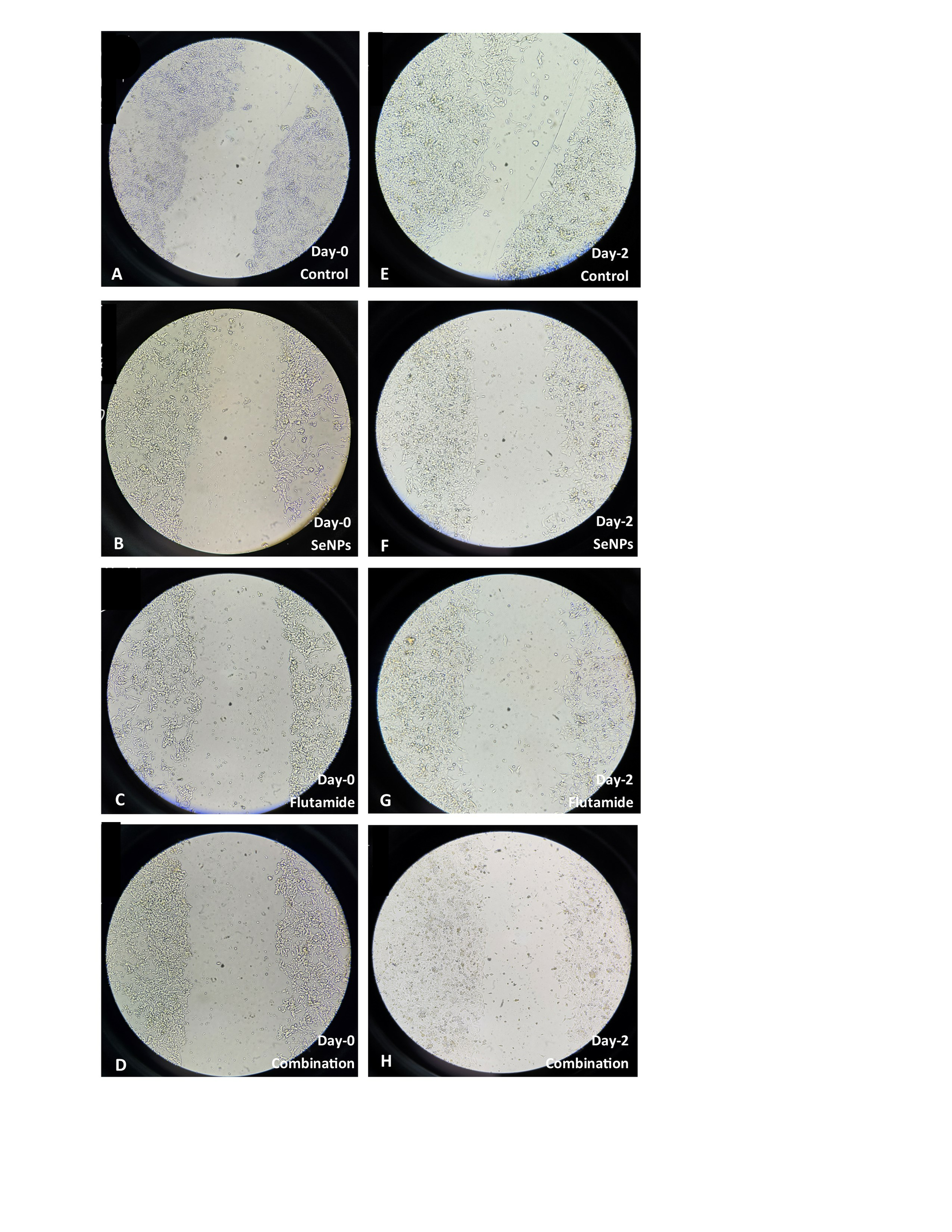

Supplement: S1 Fig — (A) control-Day 0 (B) SeNPs-Day 0 (C) Flutamide-Day 0 (D) Combination-Day 0 (E) control-Day 2 (F) SeNPs-Day 2 (G) Flutamide-Day 2 (H) combination-Day 2. (TIF) [file pone.0318483.s001.tif]

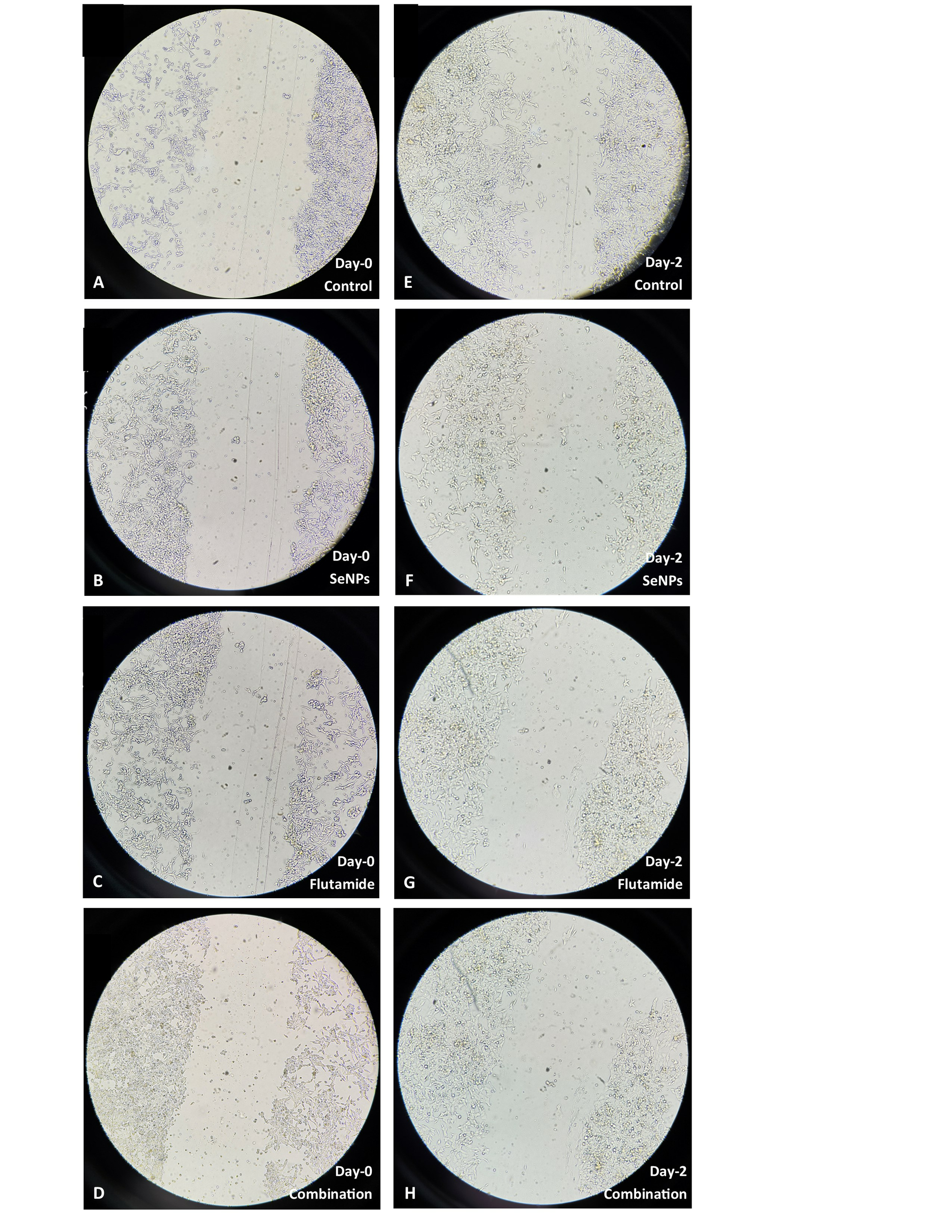

Supplement: S2 Fig — (A) control-Day 0 (B) SeNPs-Day 0 (C) Flutamide-Day 0 (D) Combination-Day 0 (E) control-Day 2 (F) SeNPs-Day 2 (G) Flutamide-Day 2 (H) combination-Day 2. (TIF) [file pone.0318483.s002.tif]
