# Supplementary material for: Reducing the effective dosage of flutamide on prostate cancer cell lines through combination with selenium nanoparticles: An in-vitro study
Source: PLoS One. 2025 May 19;20(5):e0318483. doi: 10.1371/journal.pone.0318483 (PMC12088047; doi:10.1371/journal.pone.0318483)

Figure 3A:

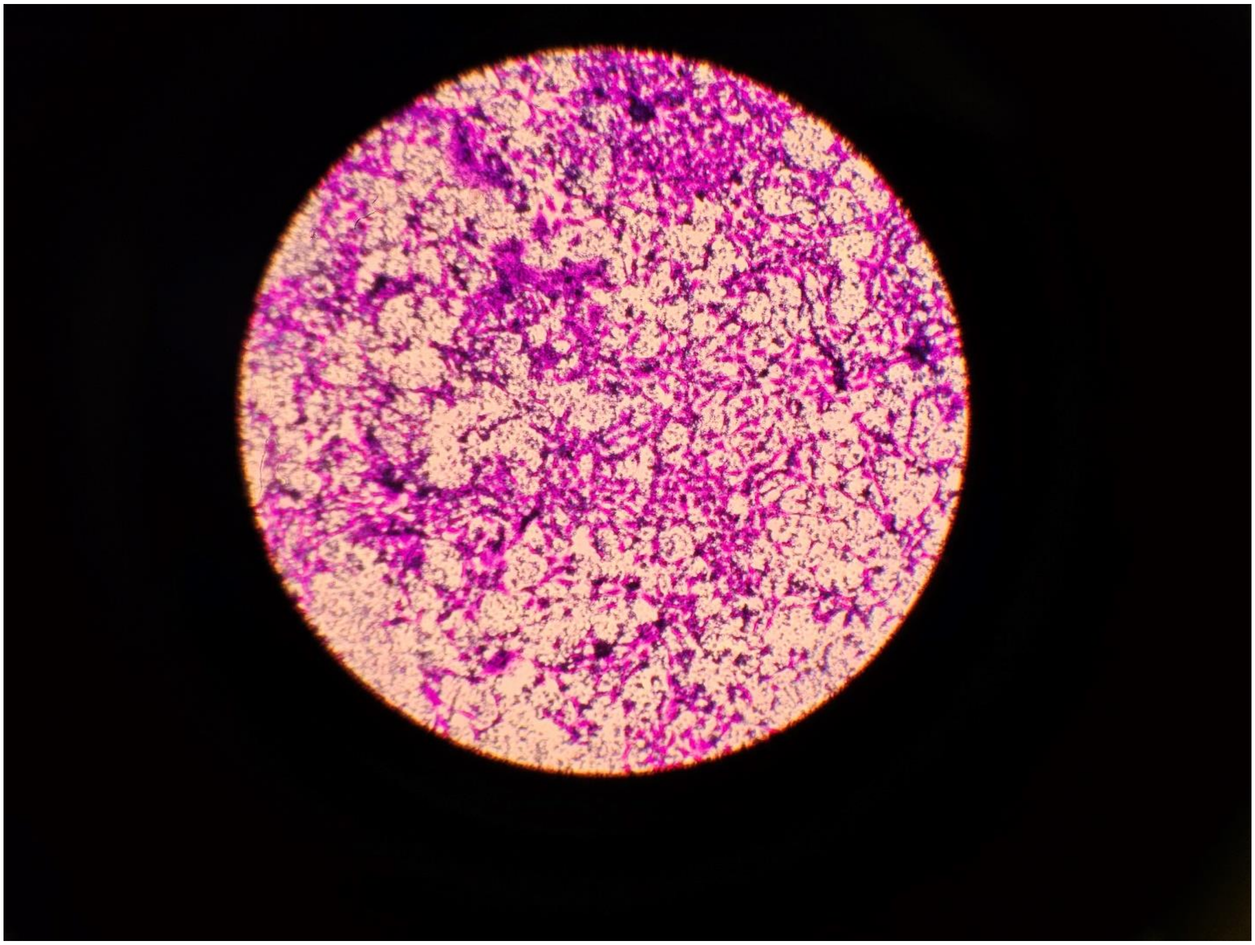

Figure 3B:

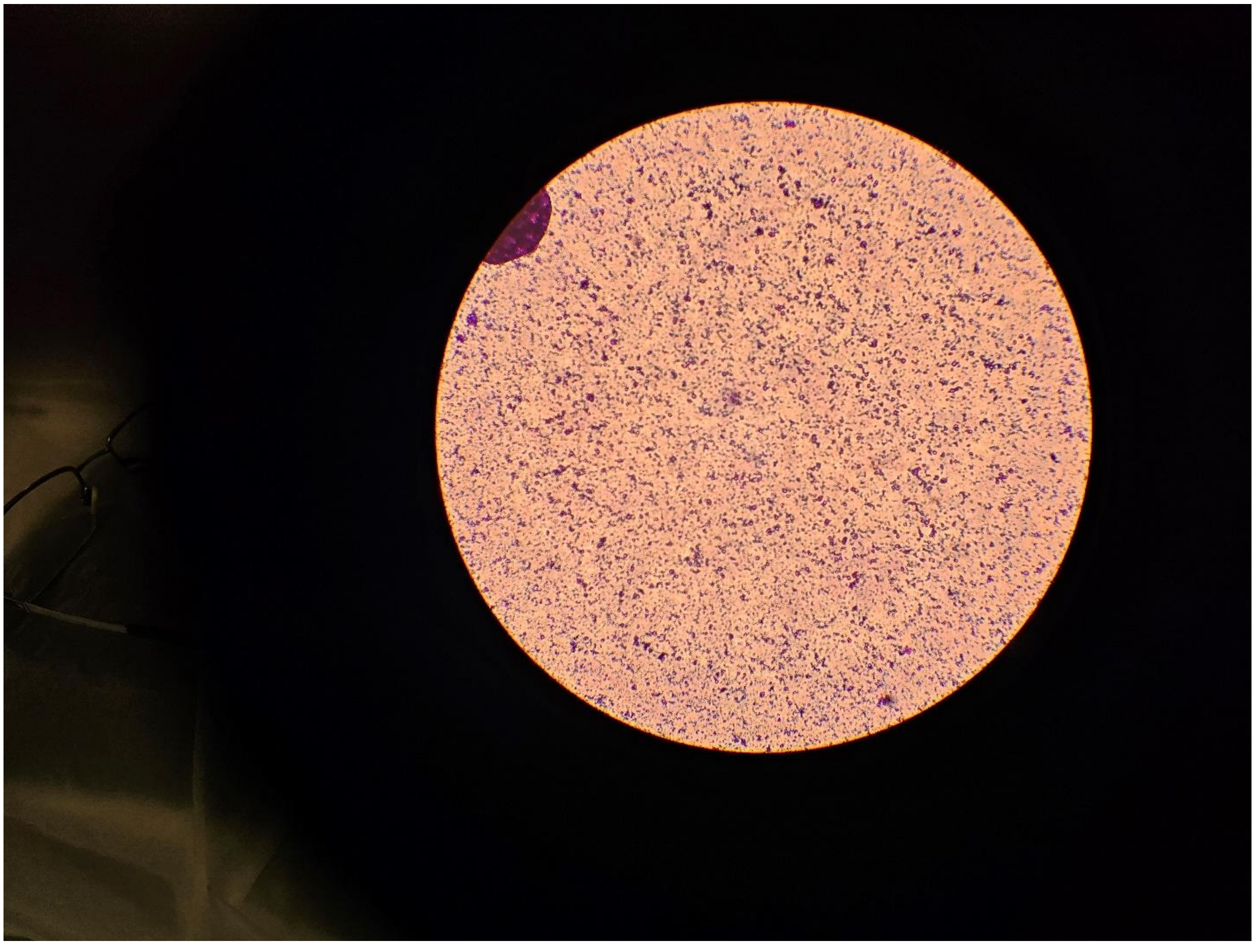

Figure 3C:

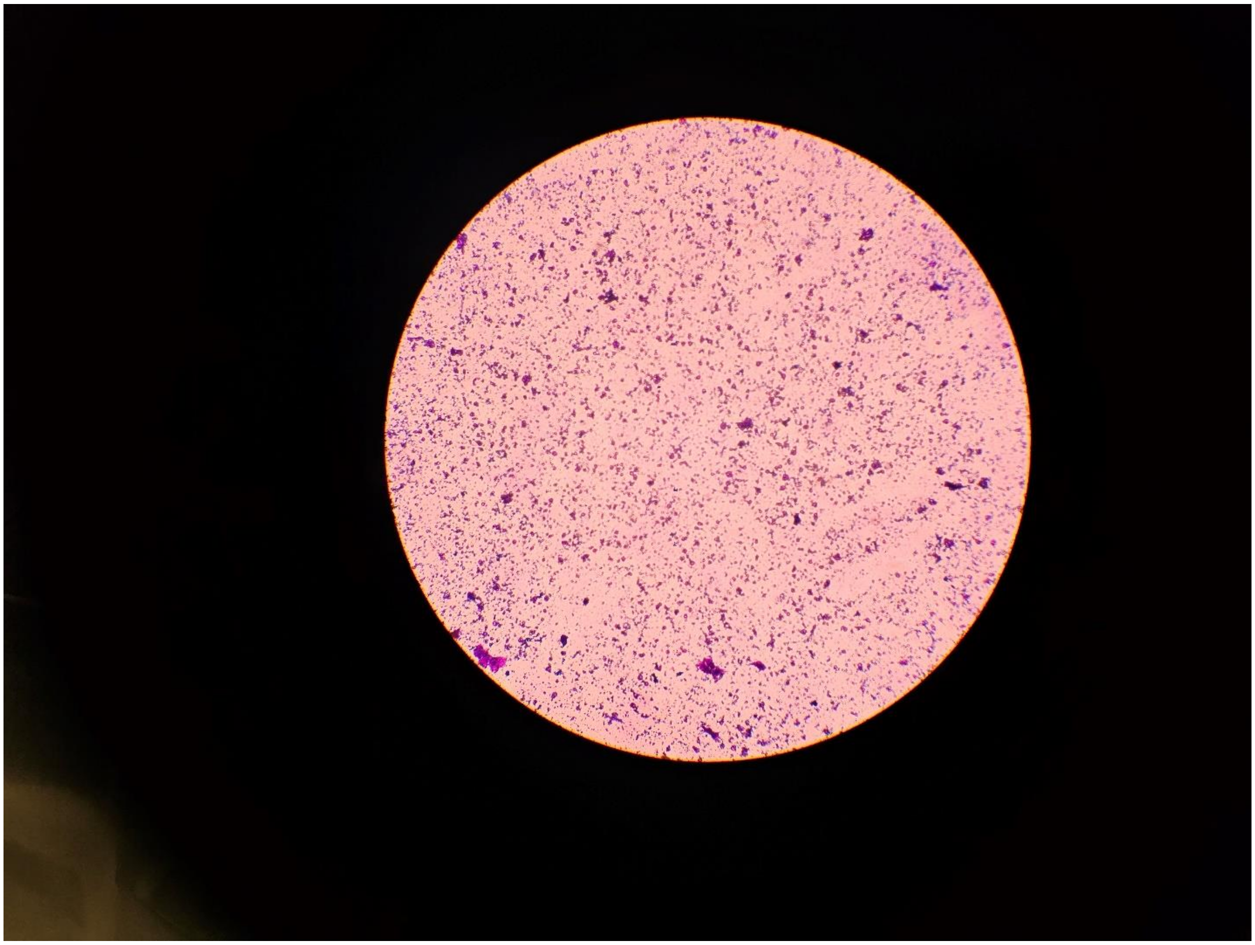

Figure 3D:

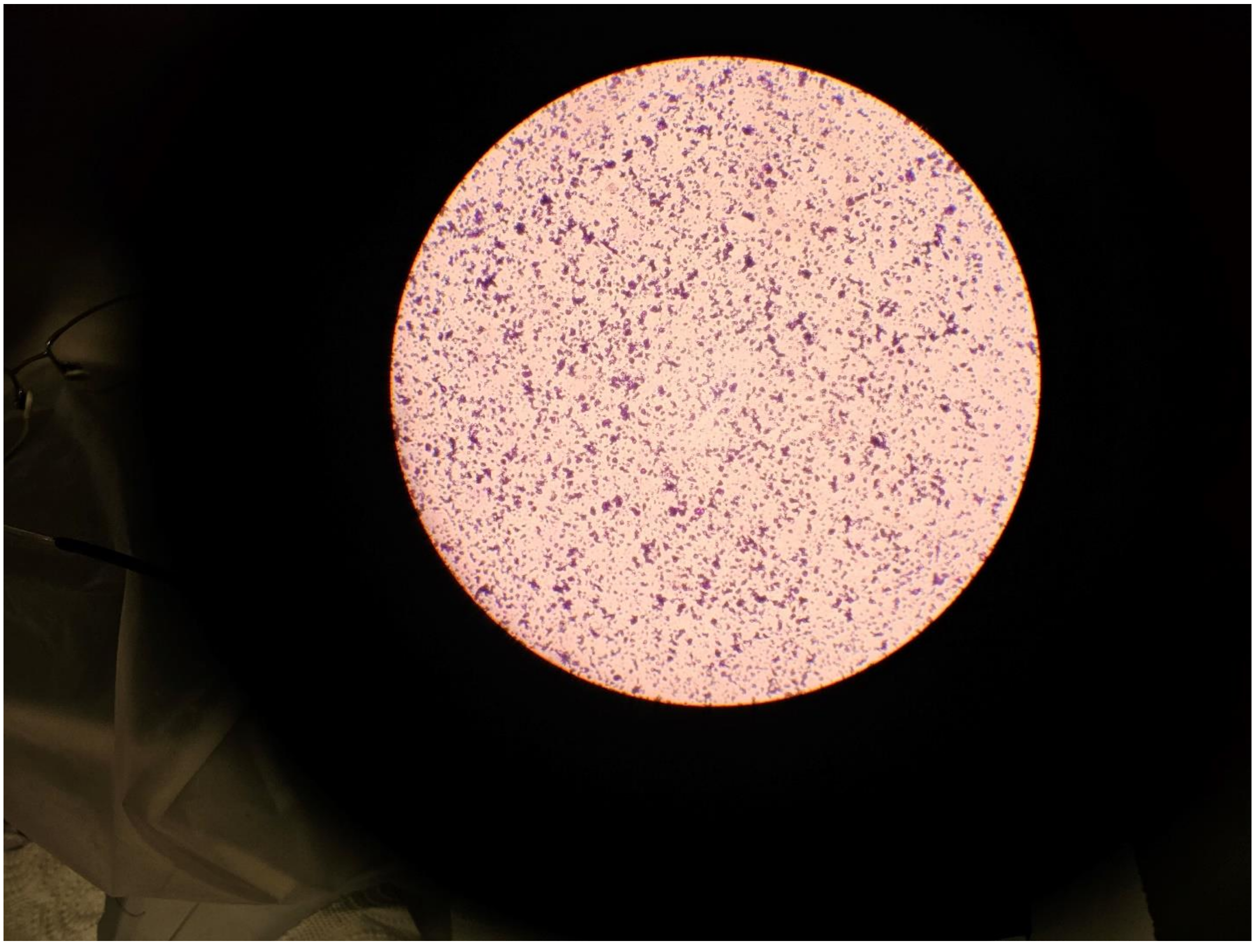

Figure 3E:

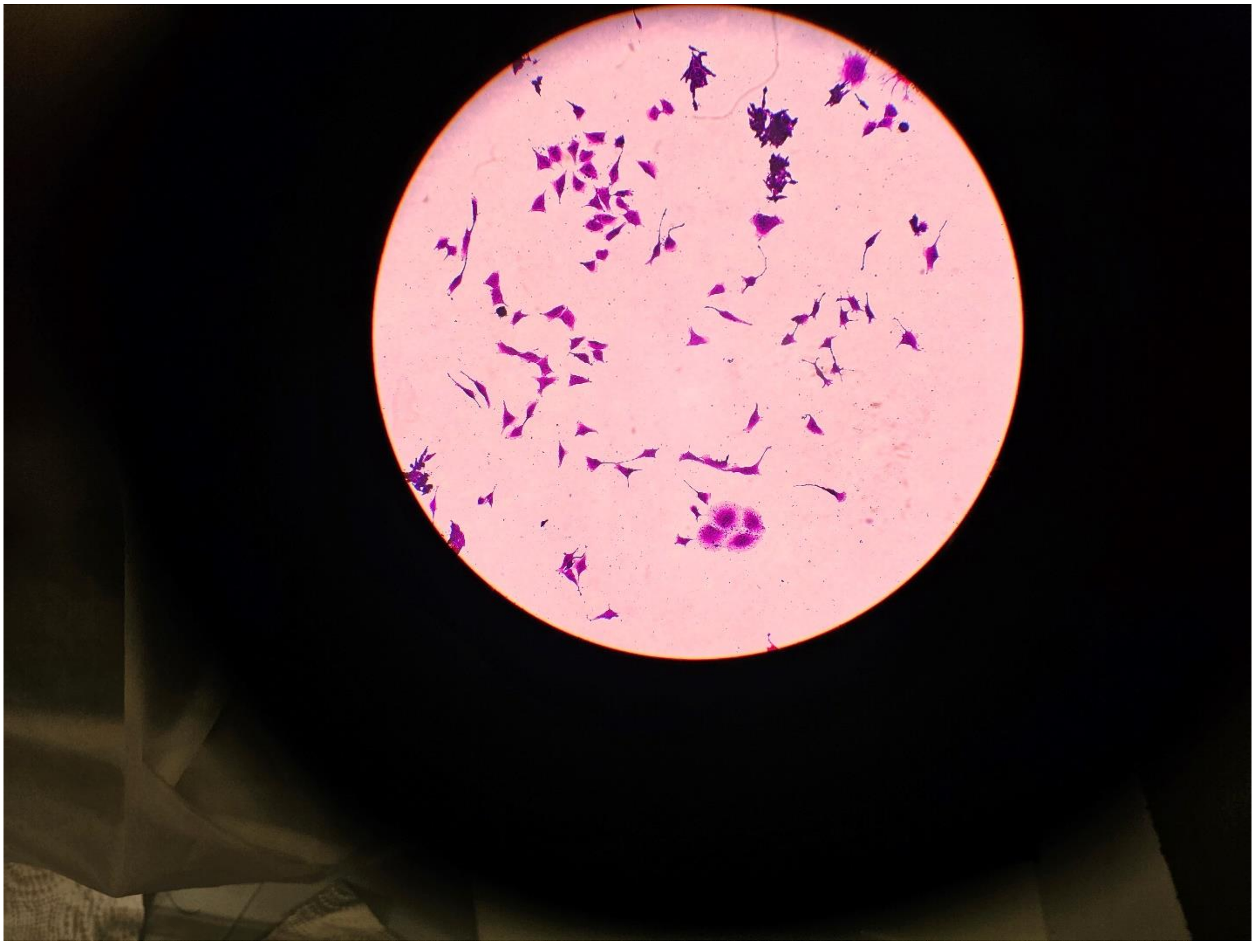

Figure 3F:

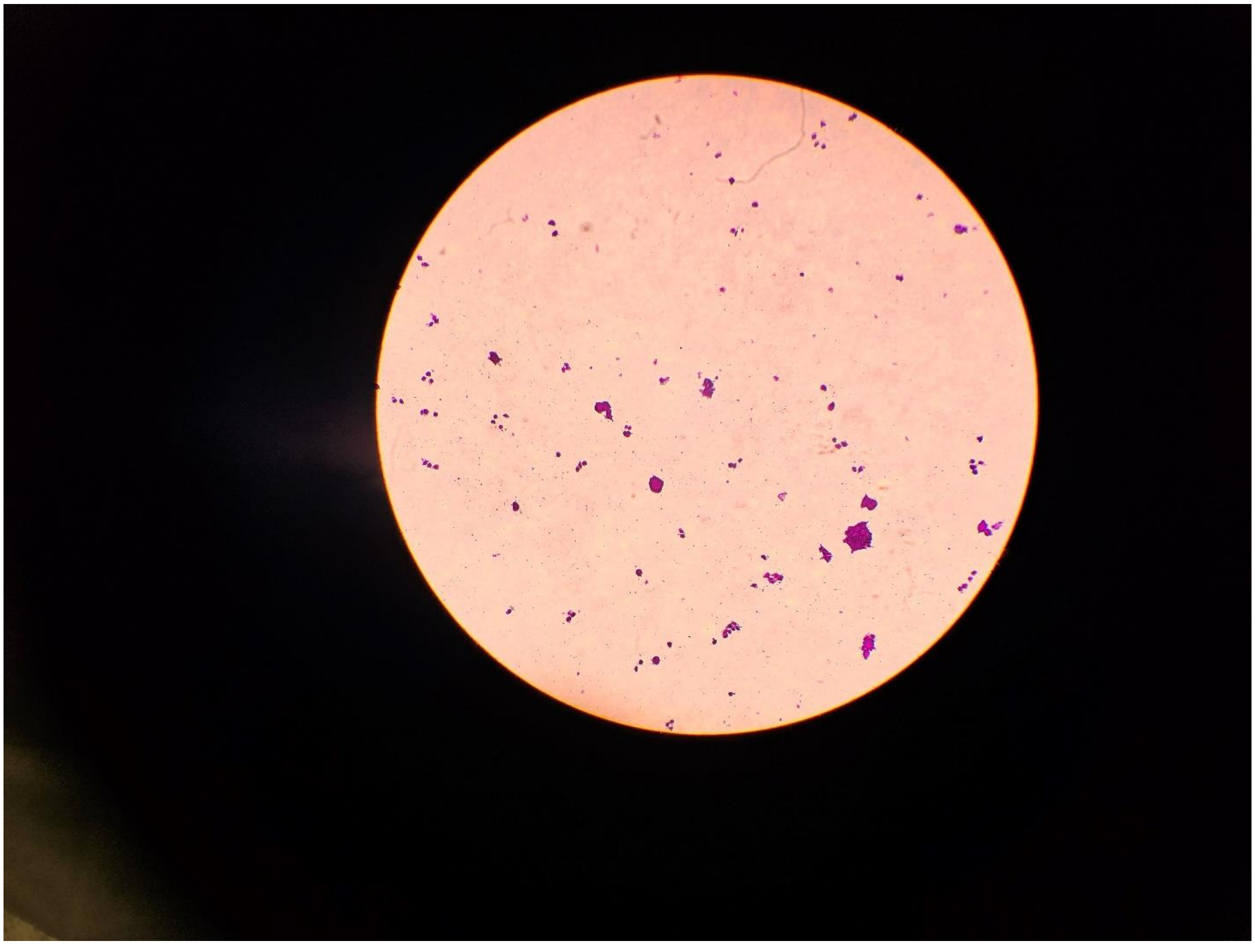

Figure 3G:

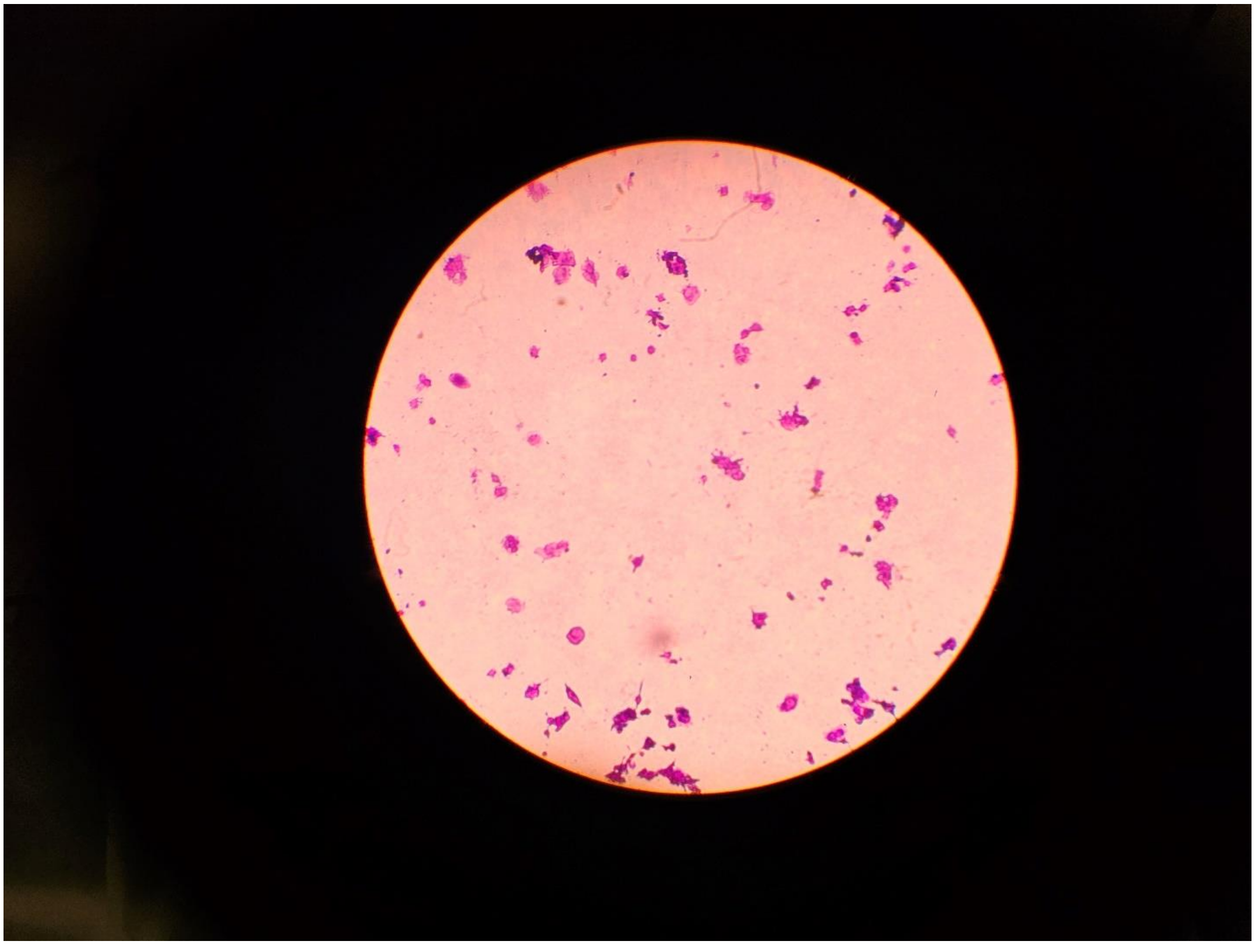

Figure 3H:

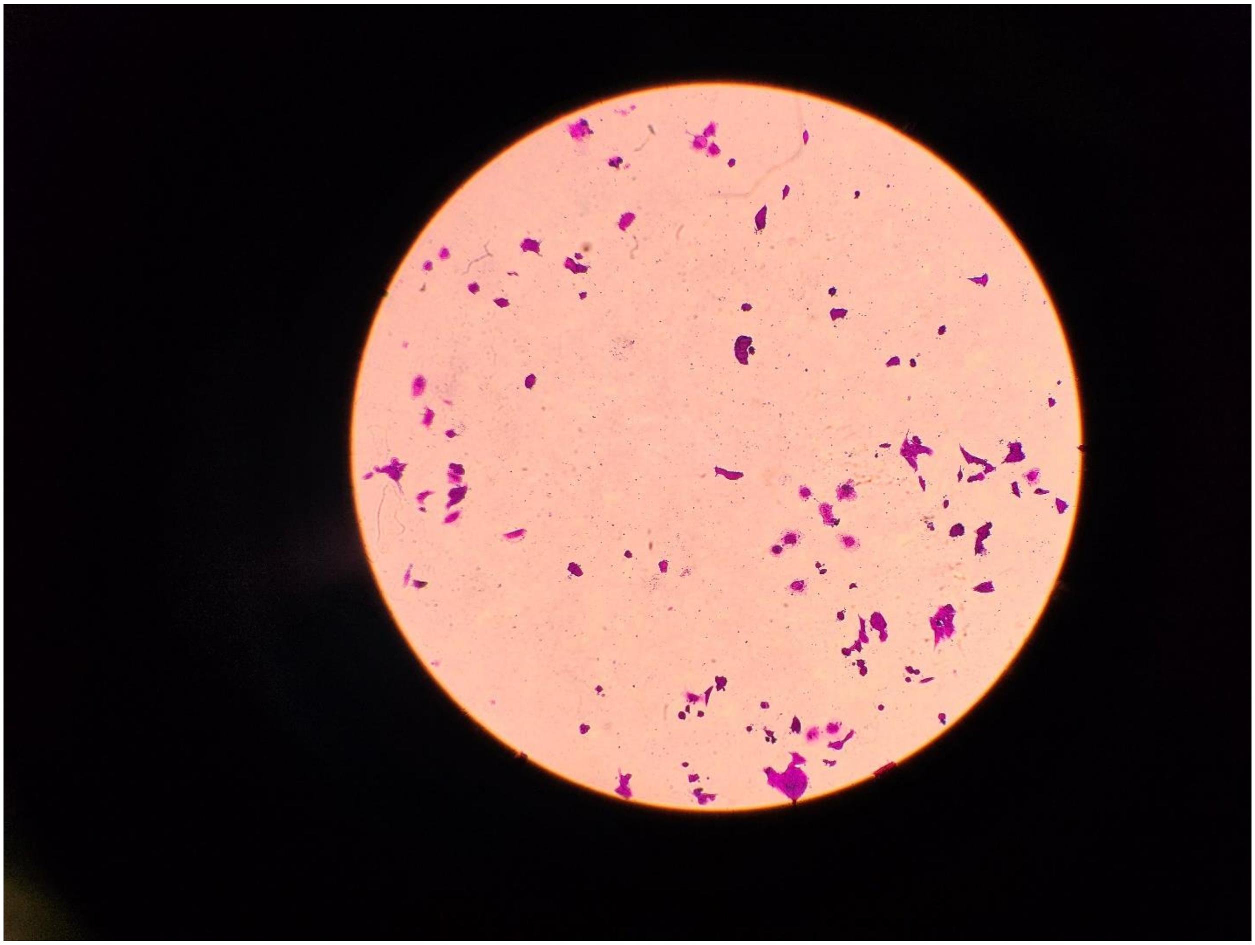

Figure 3I:

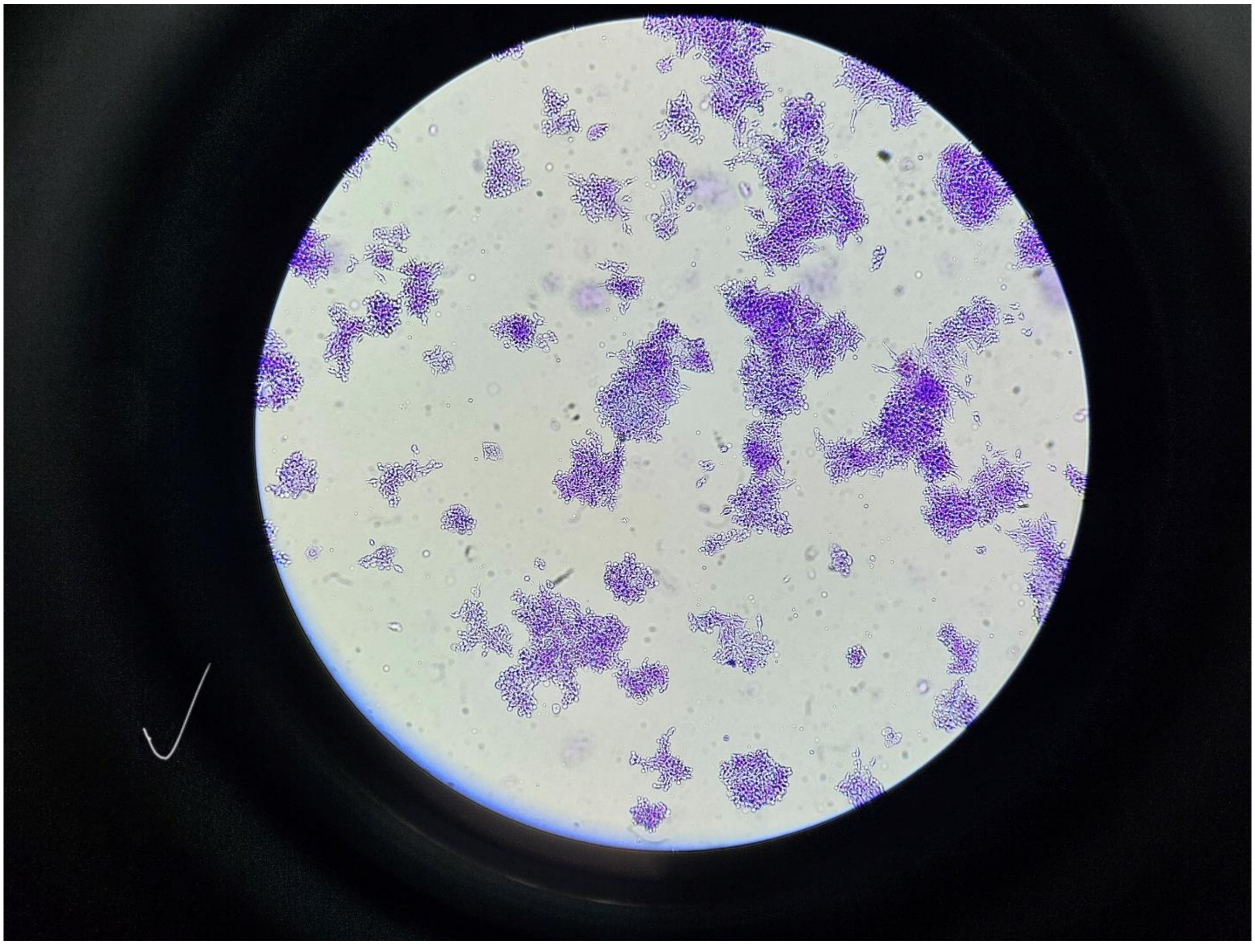

Figure 3J:

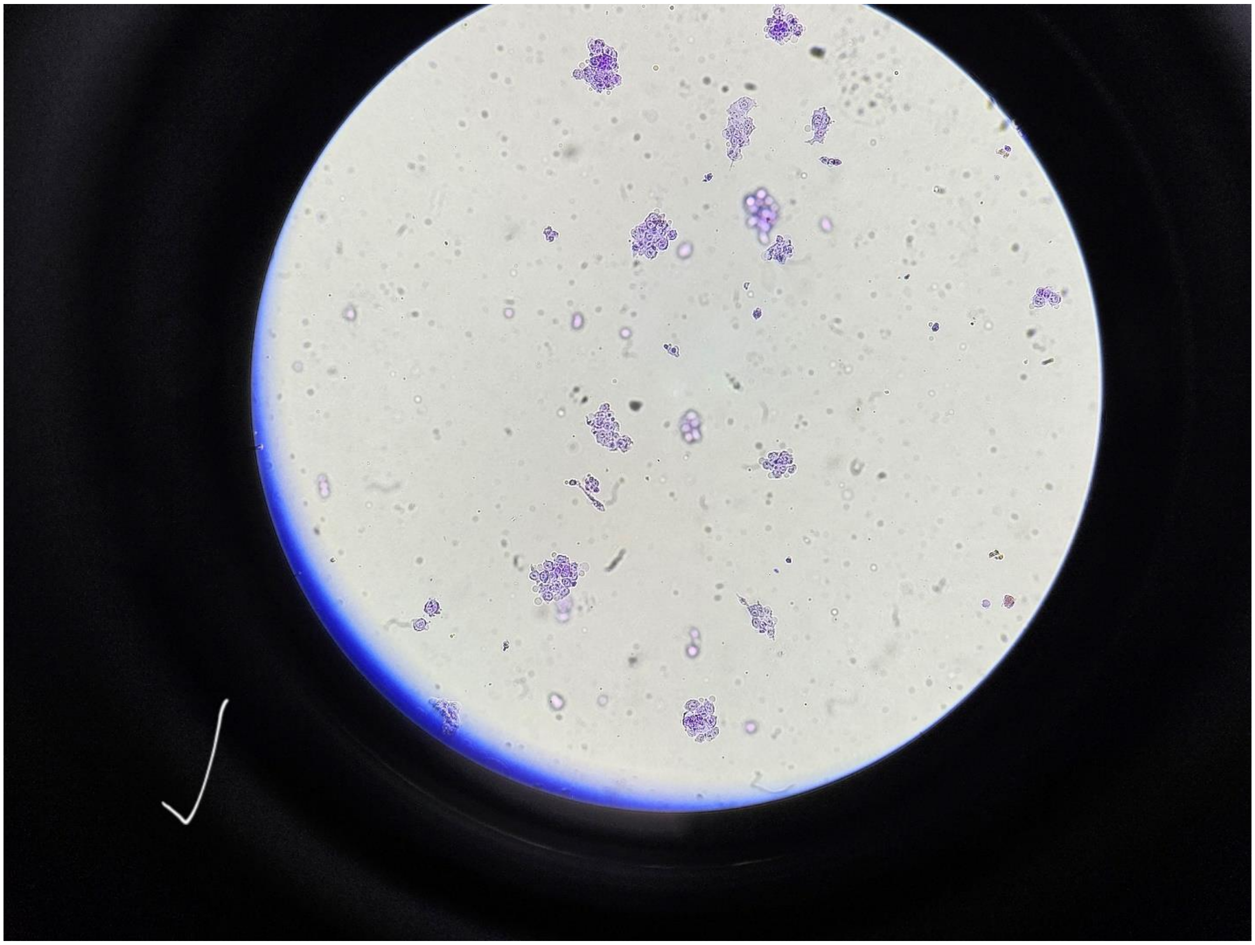

Figure 3K:

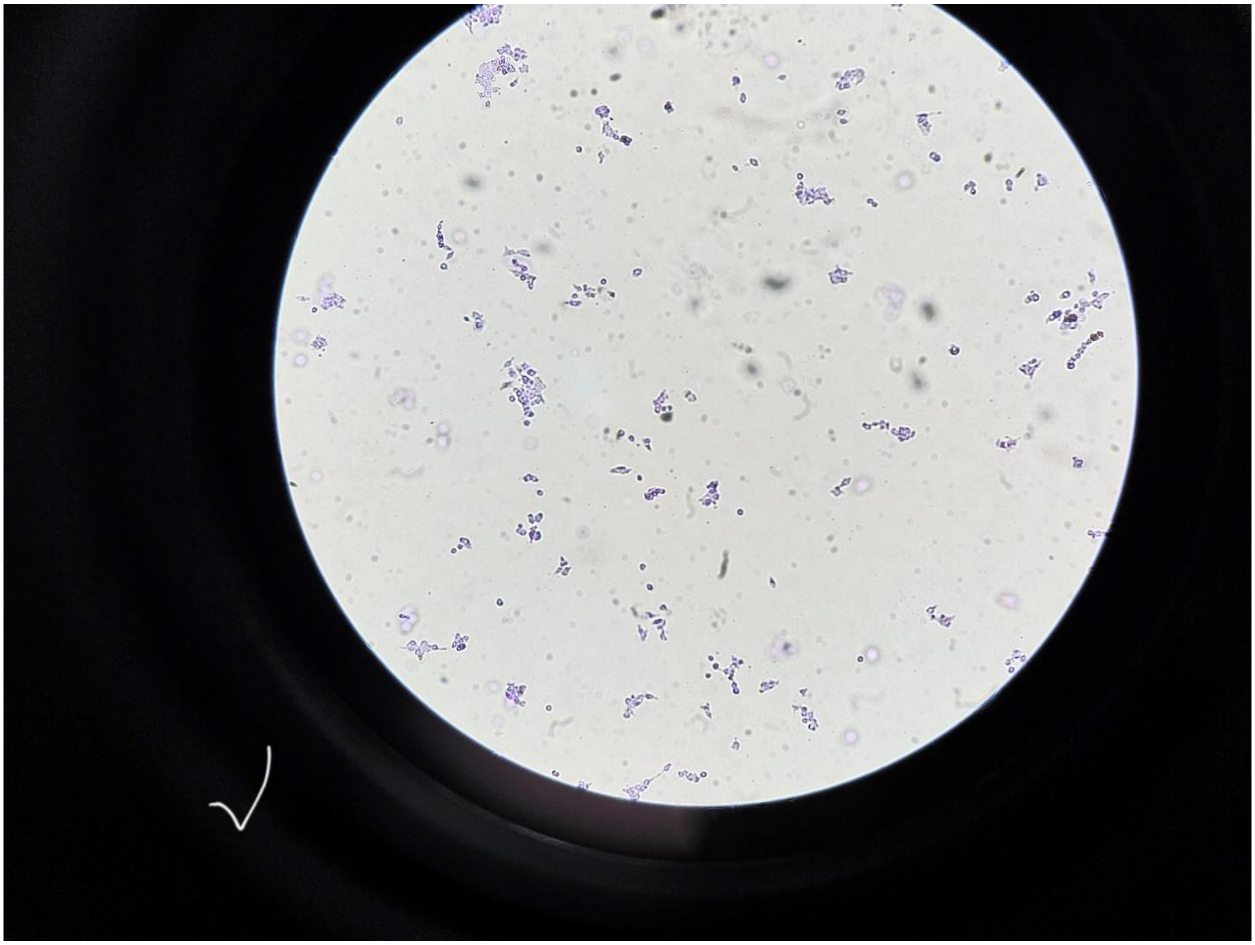

Figure 3L:

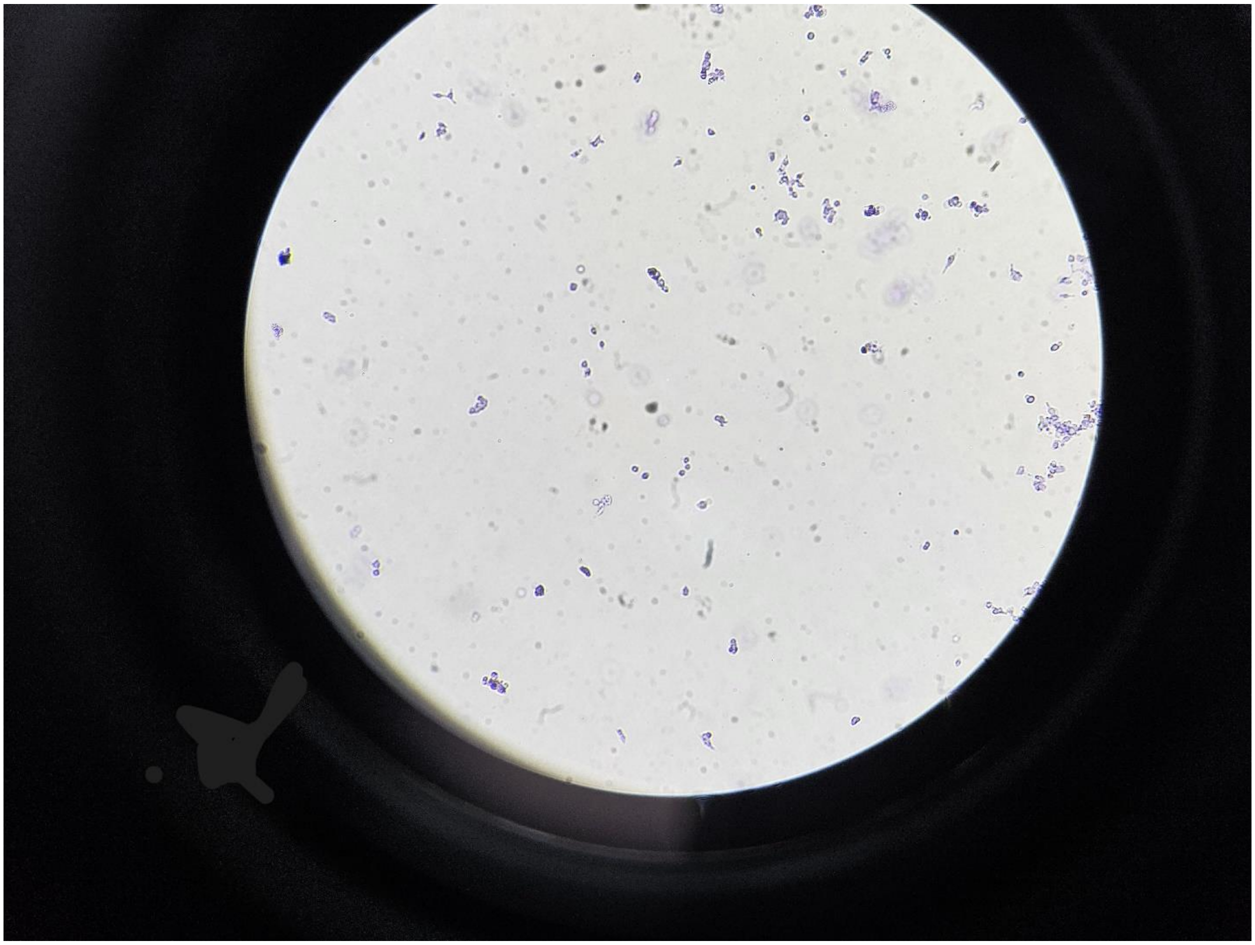

Figure 4A:

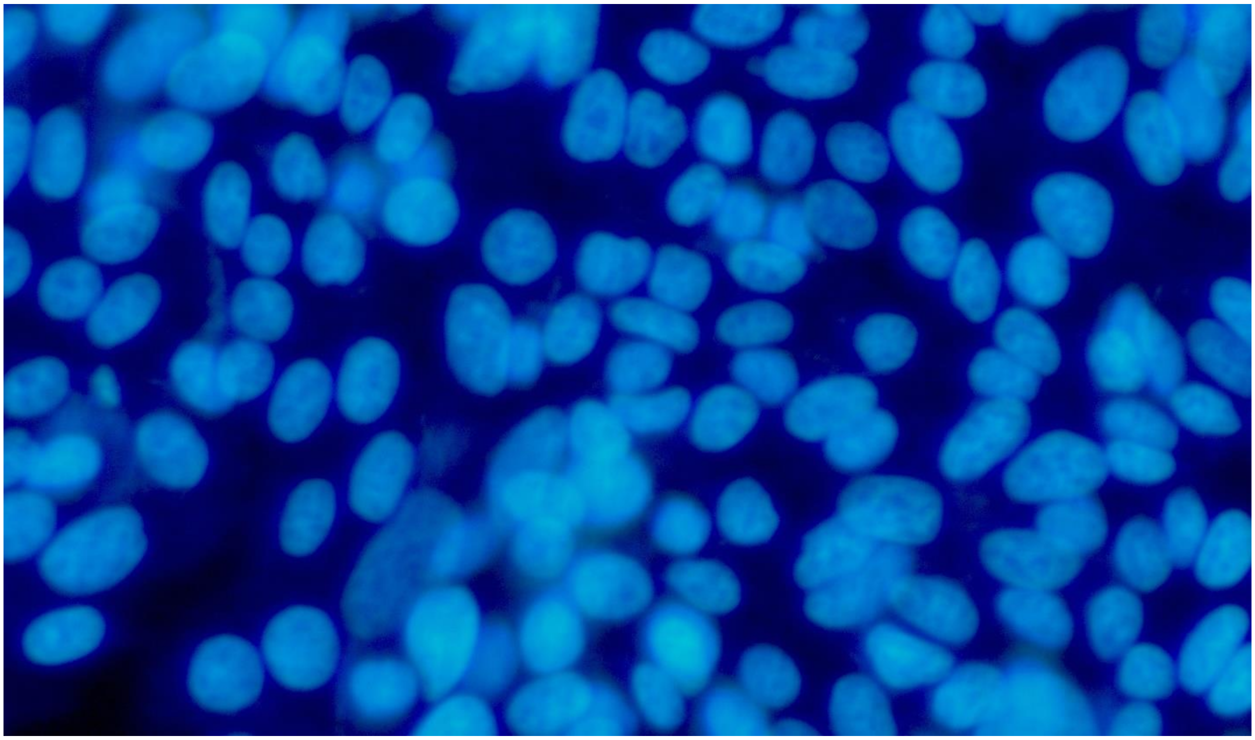

Figure 4B:

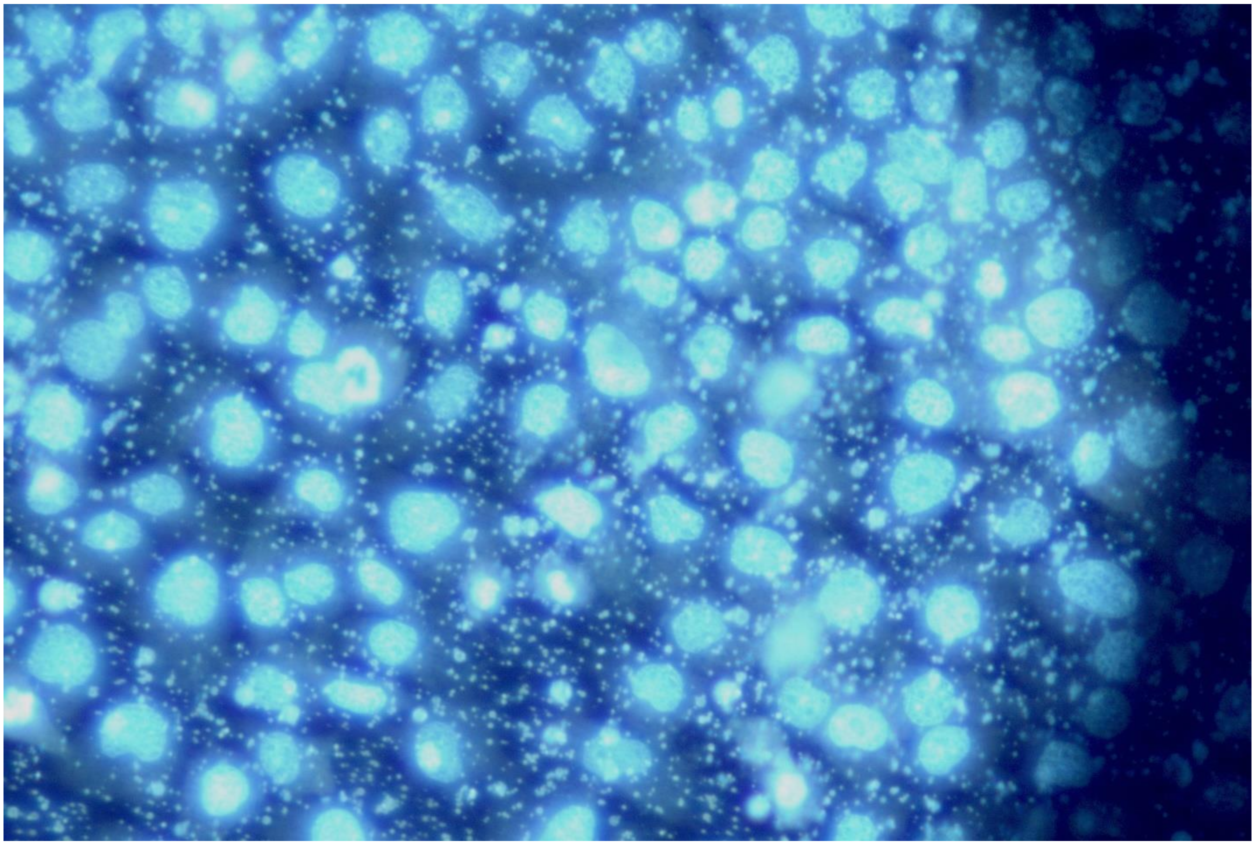

Figure 4C:

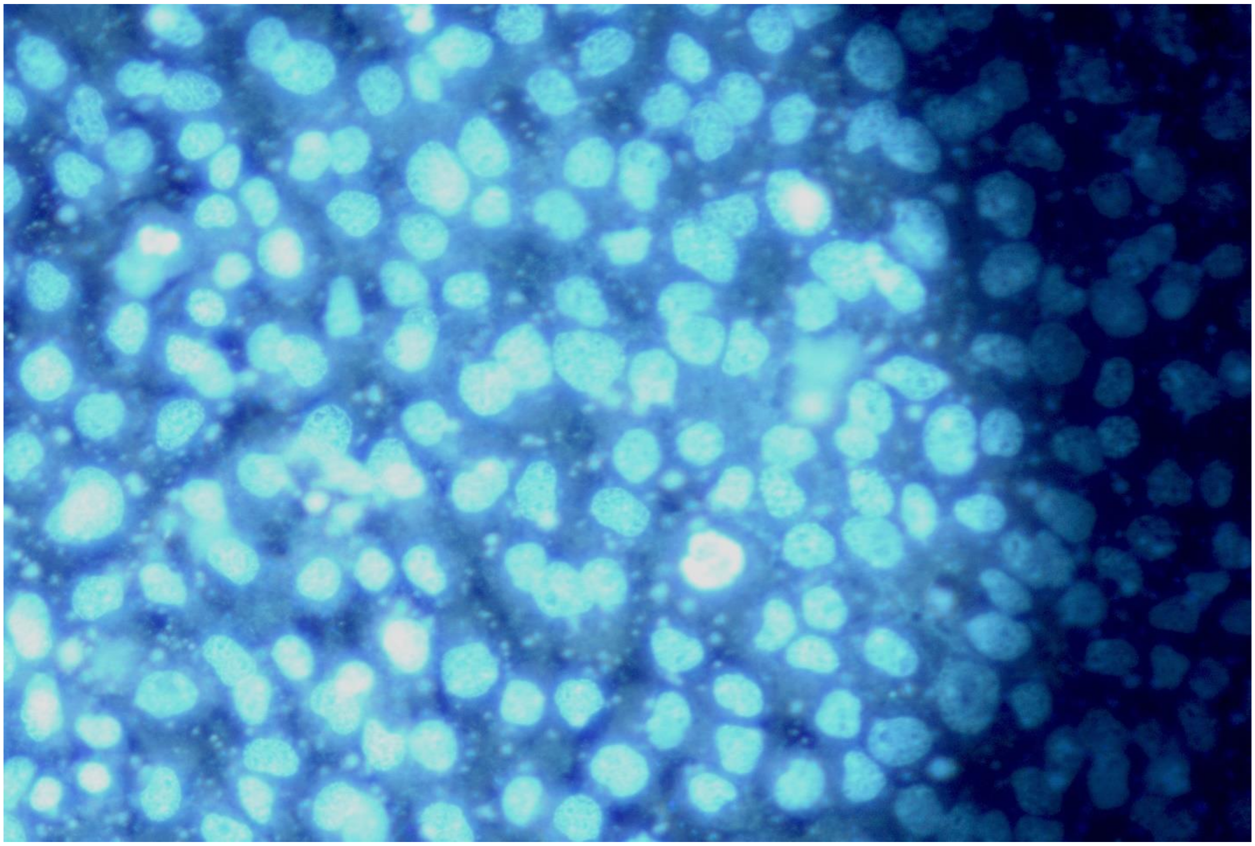

Figure 4D:

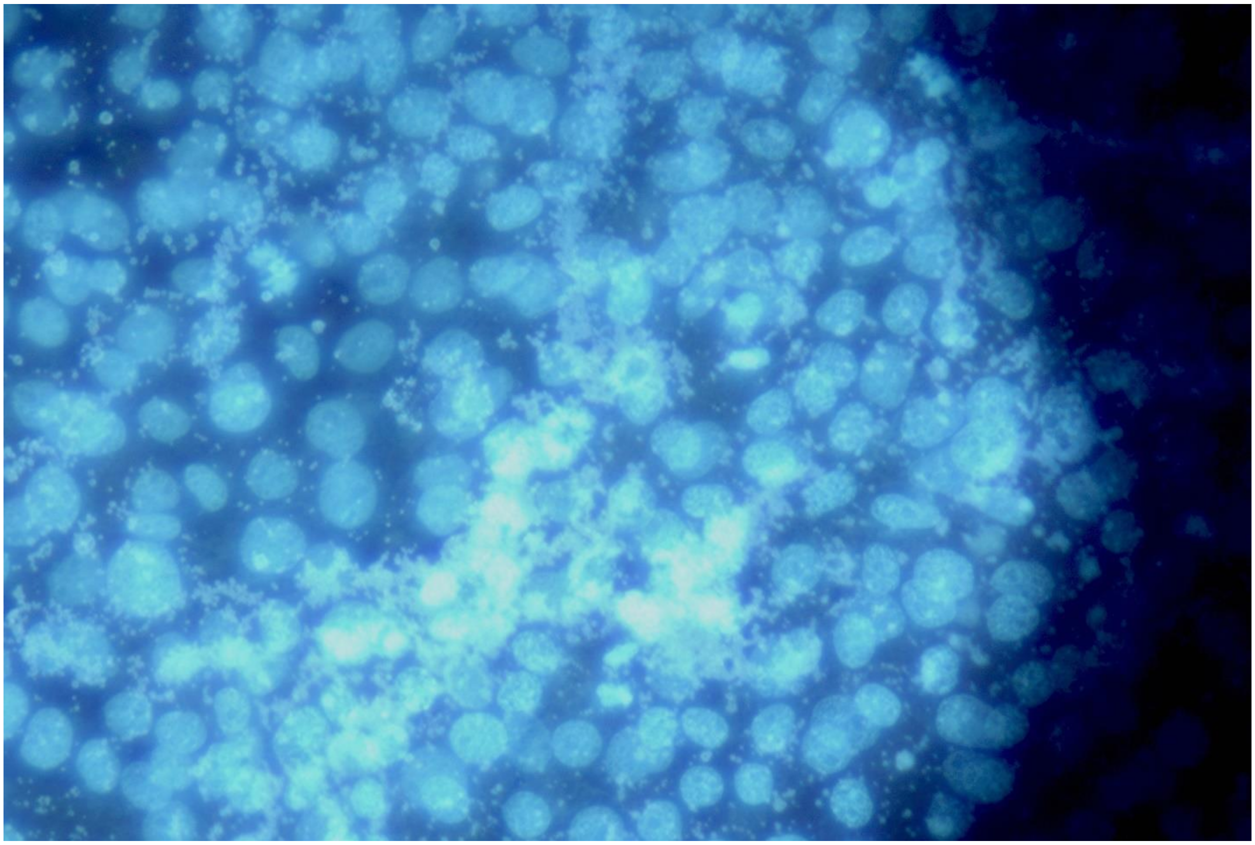

Figure 4E:

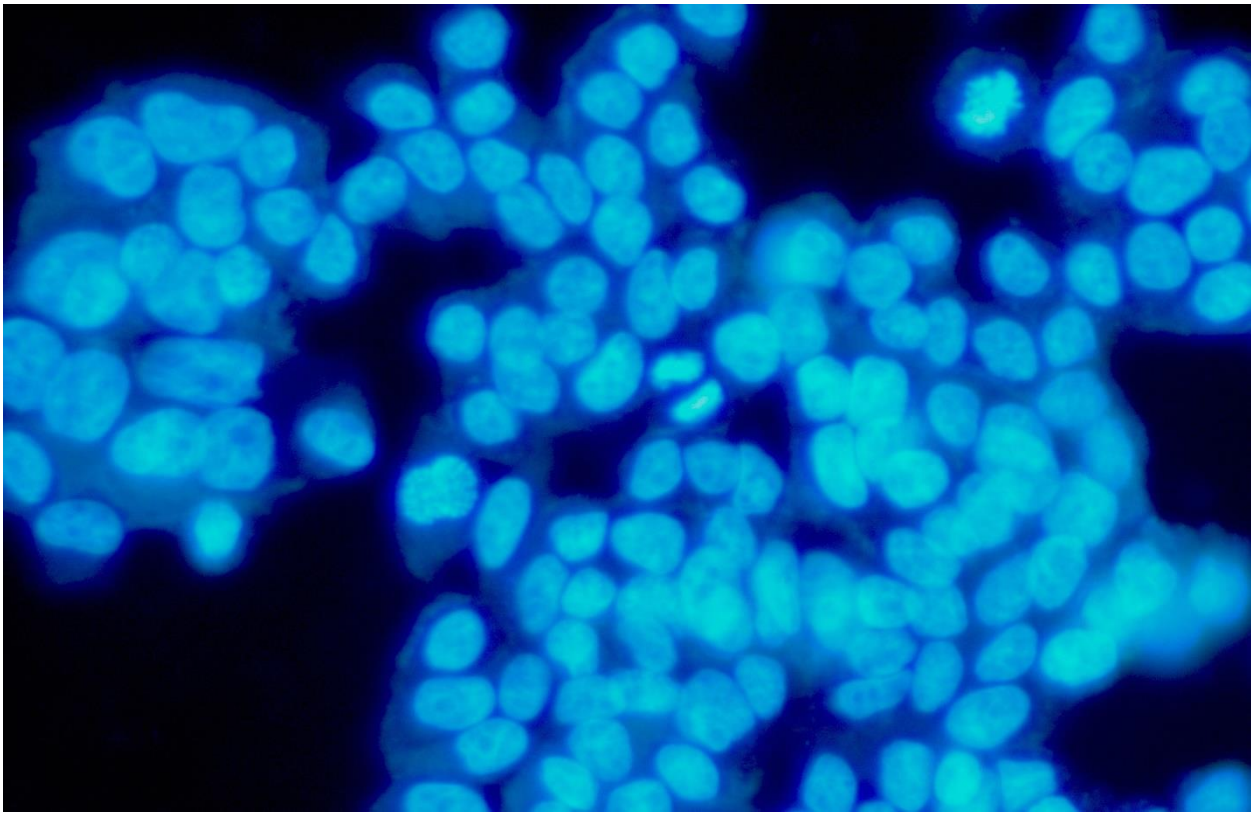

Figure 4F:

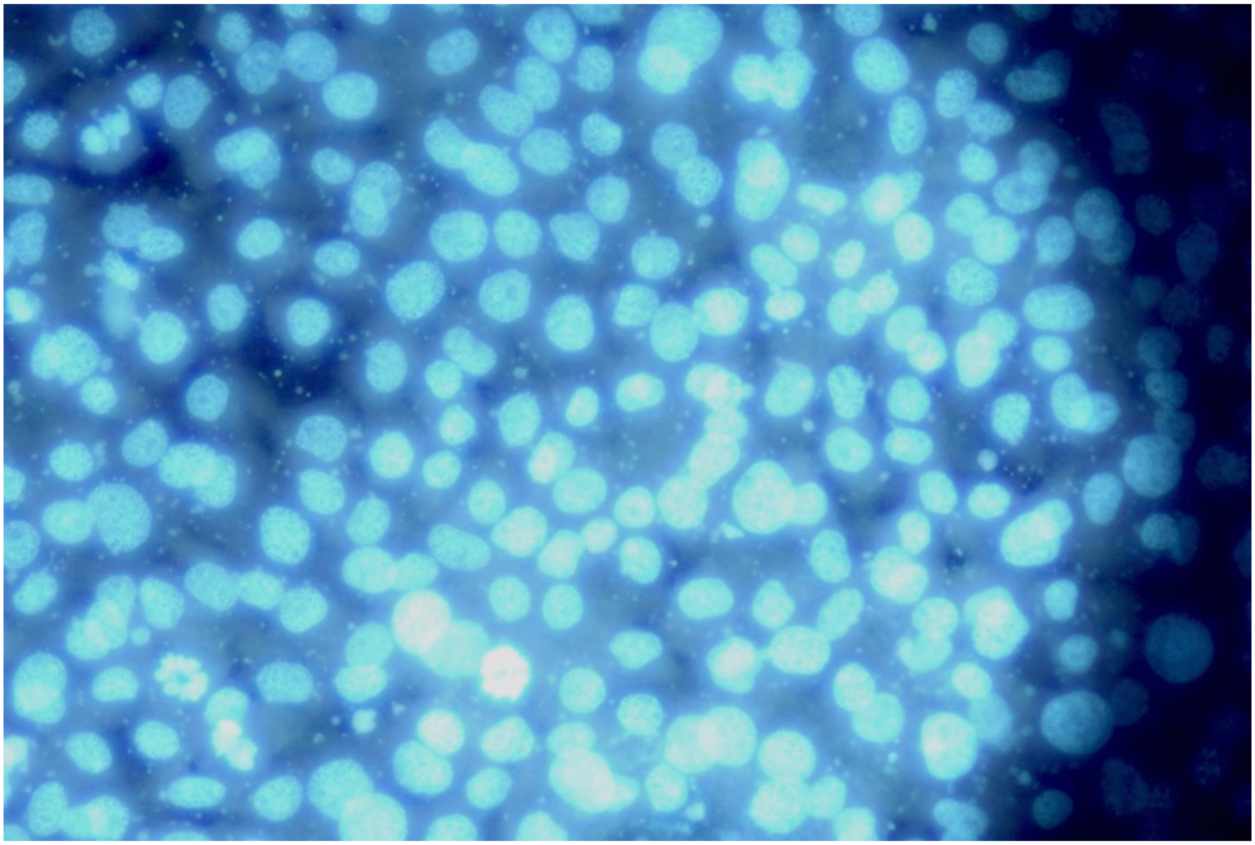

Figure 4G:

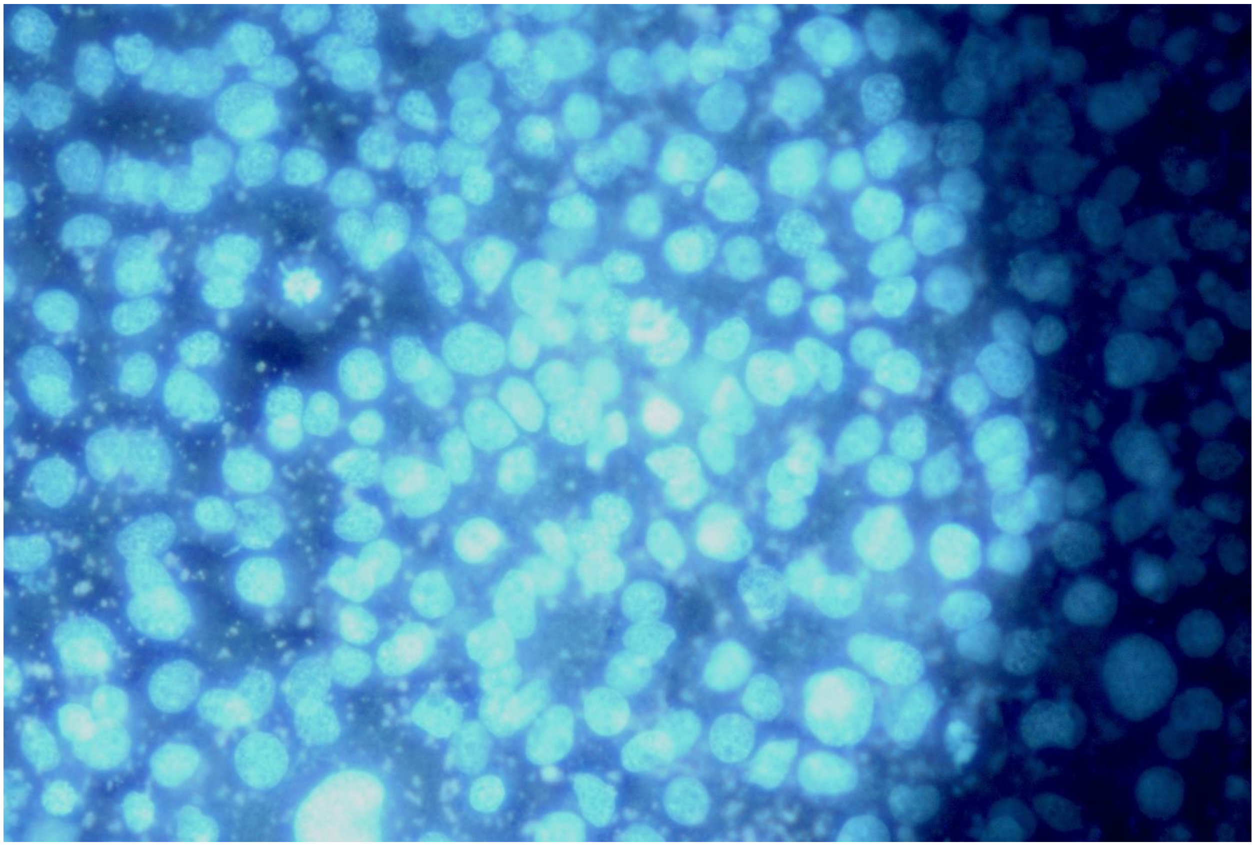

Figure 4H:

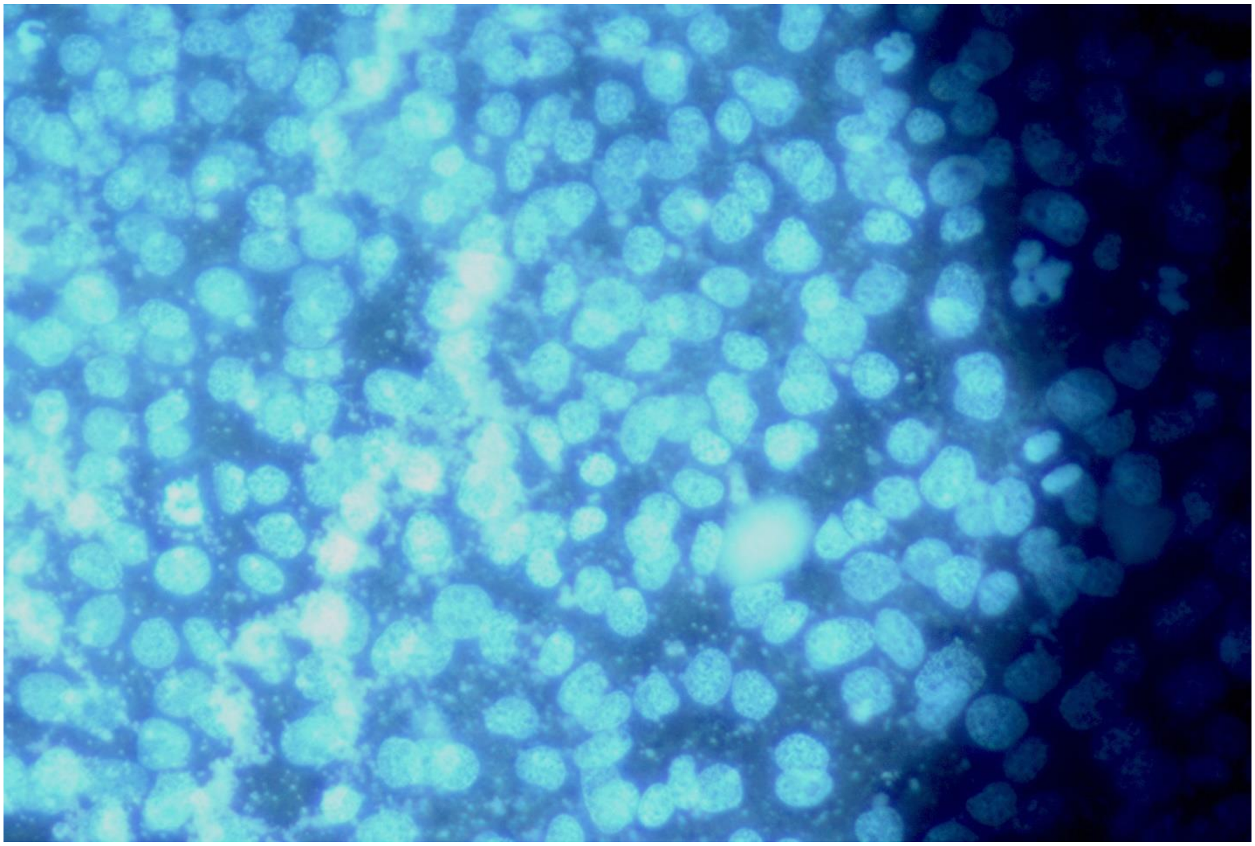

Figure 4I:

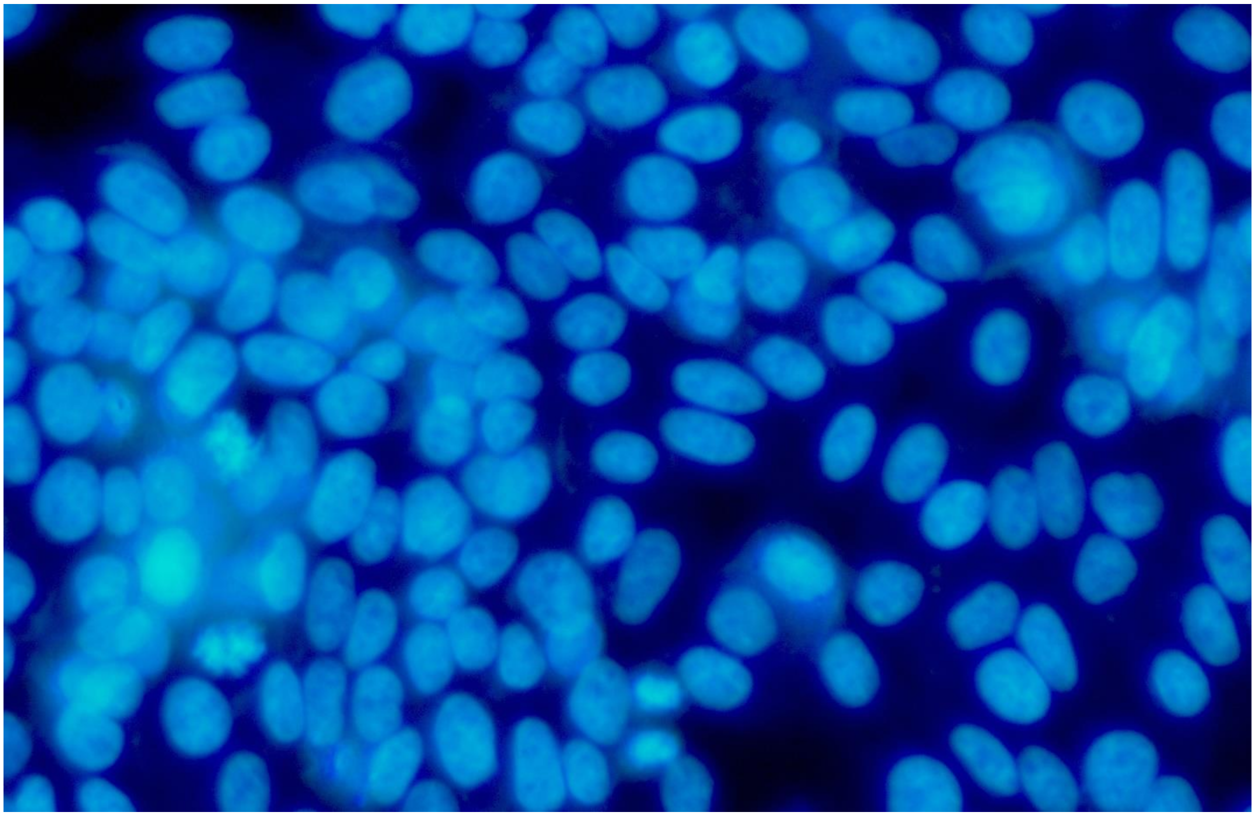

Figure 4J:

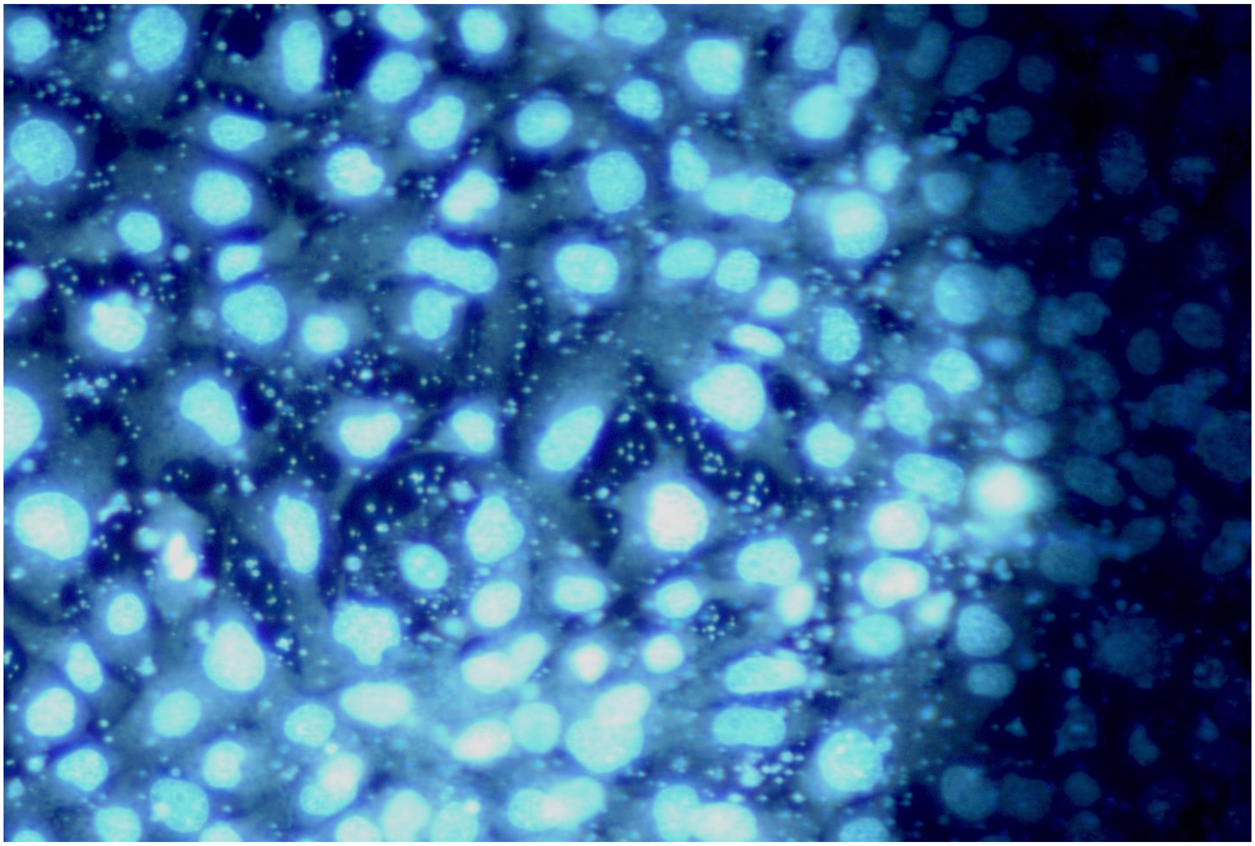

Figure 4K:

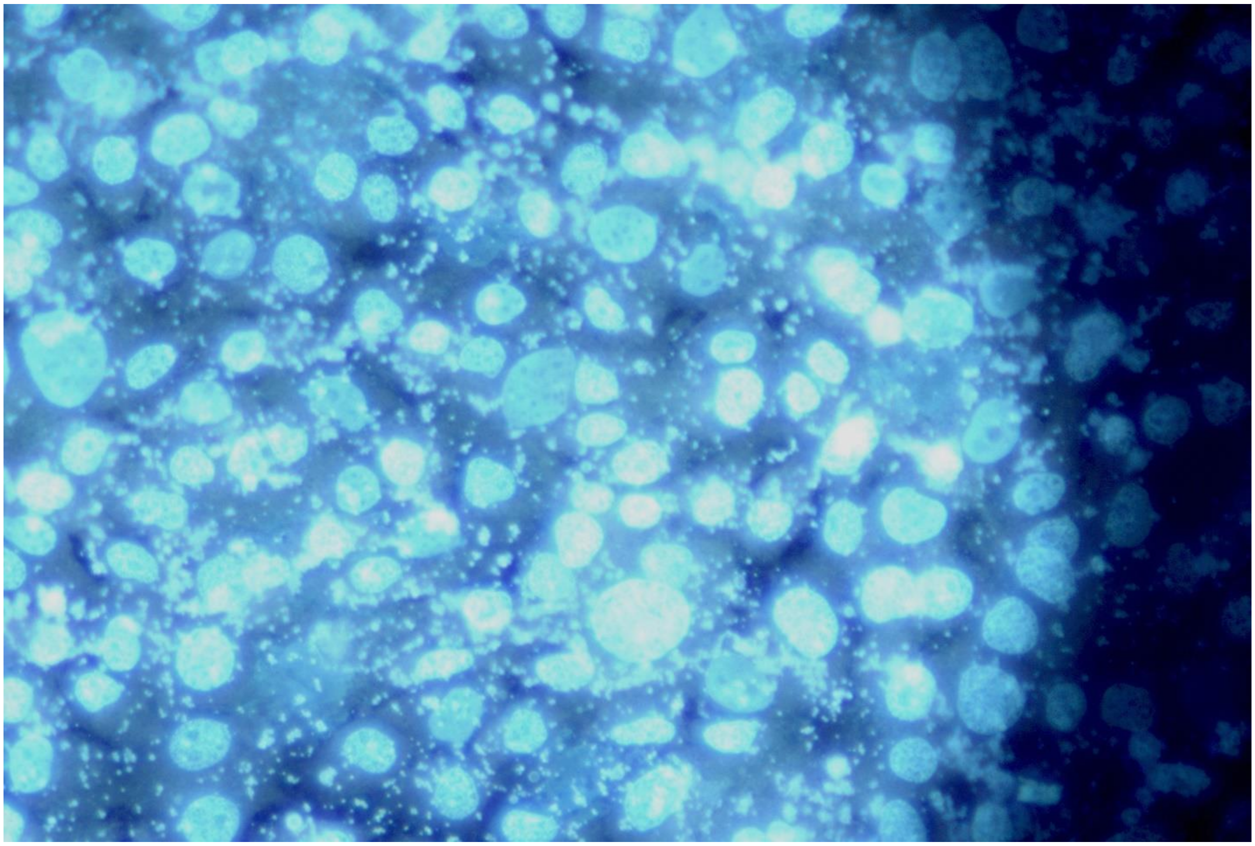

Figure 4L:

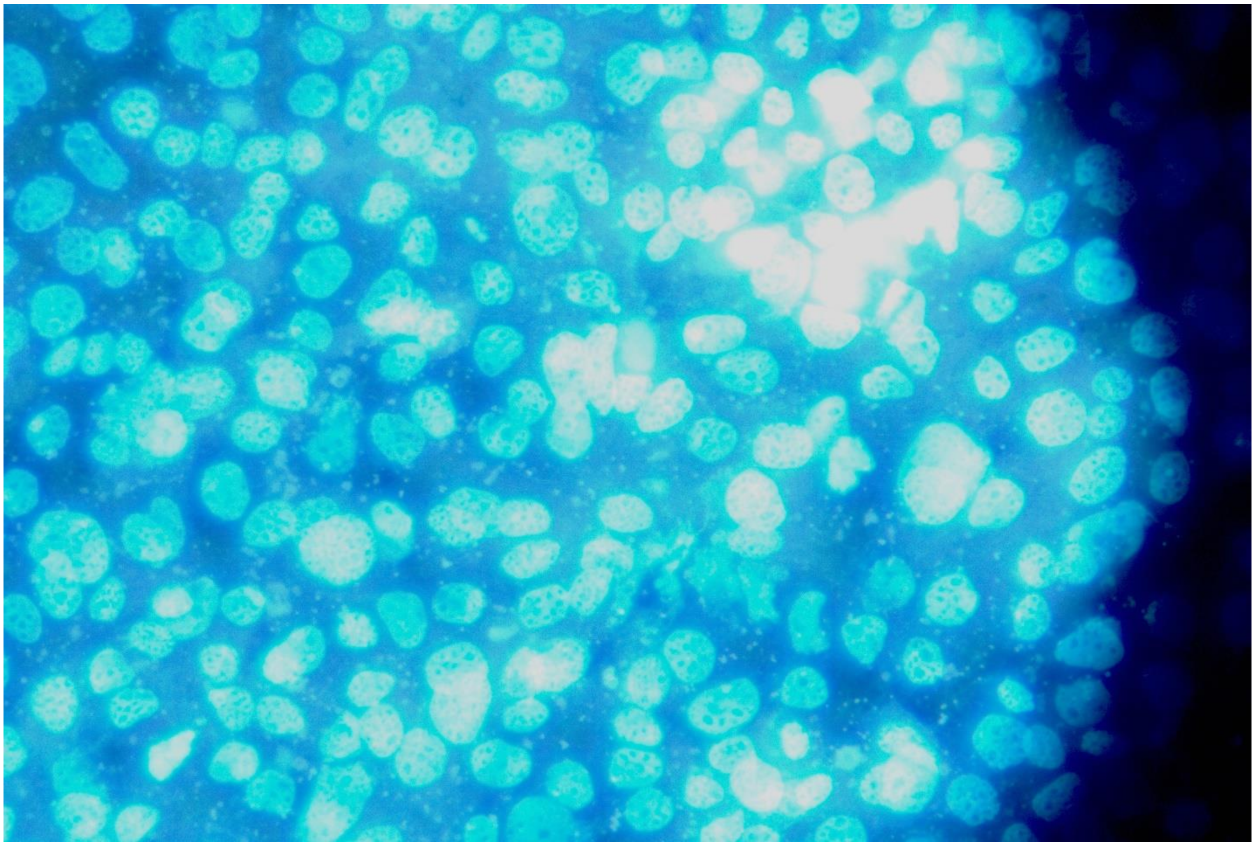

Figure 5A:

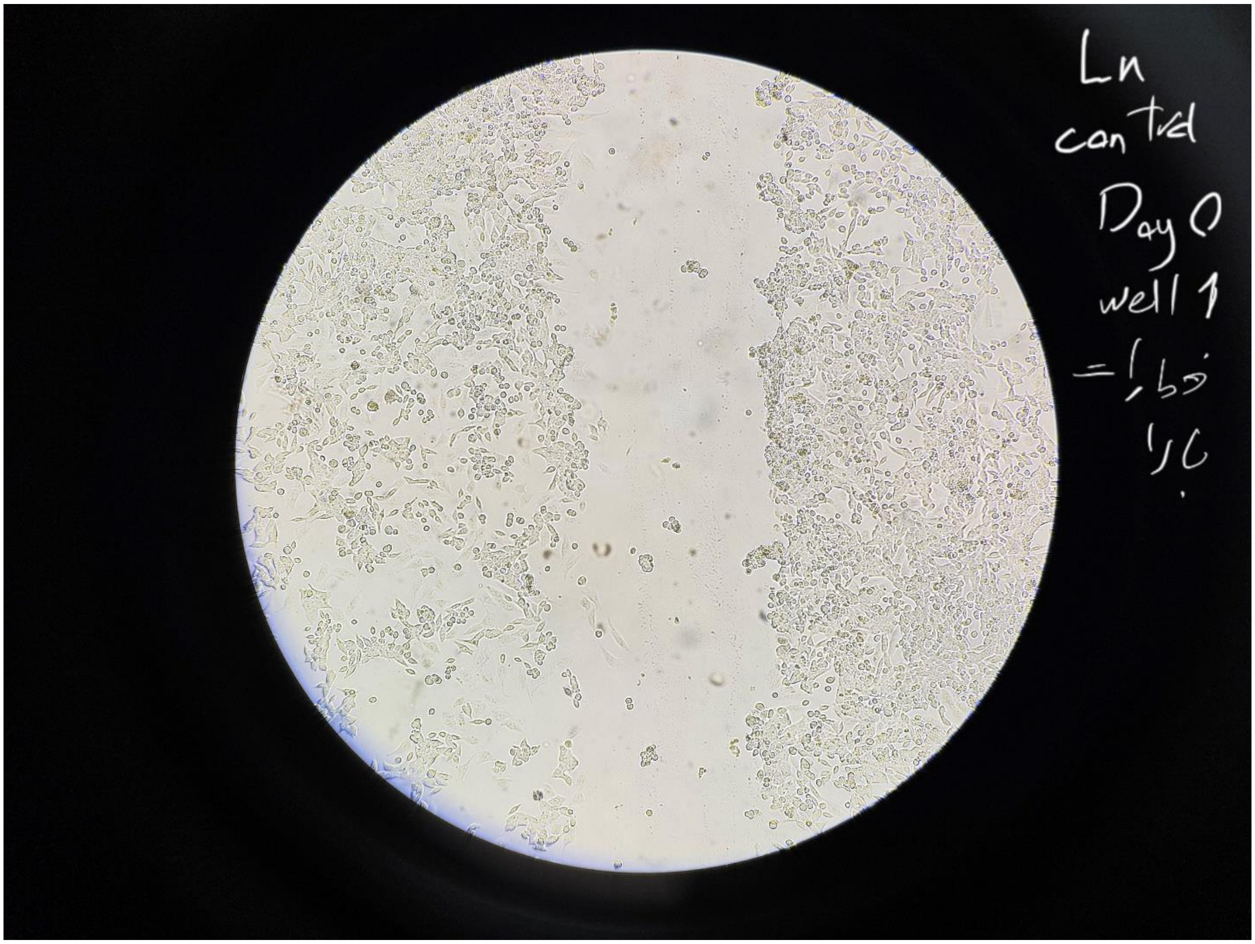

Figure 5B:

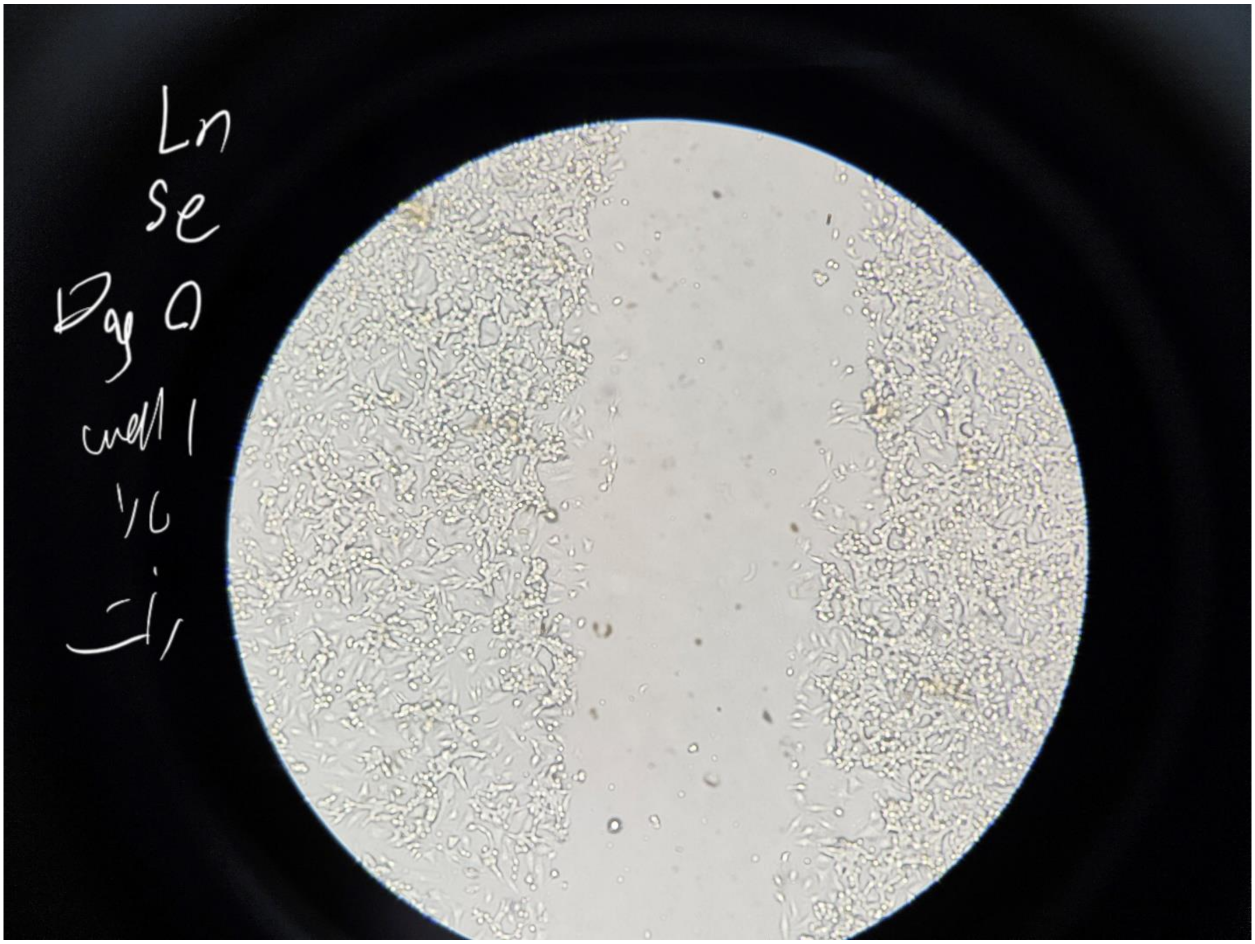

Figure 5C:

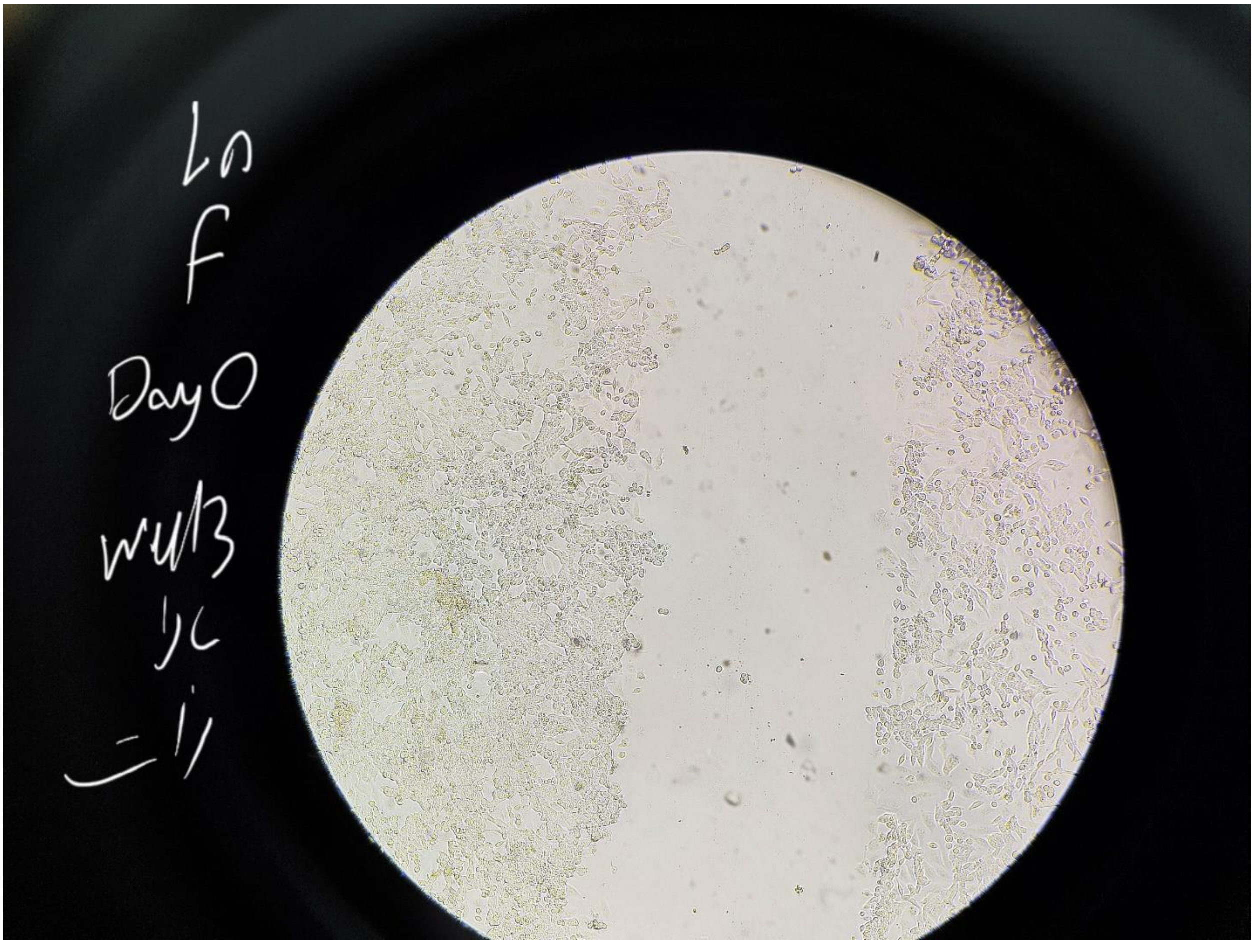

Figure 5D:

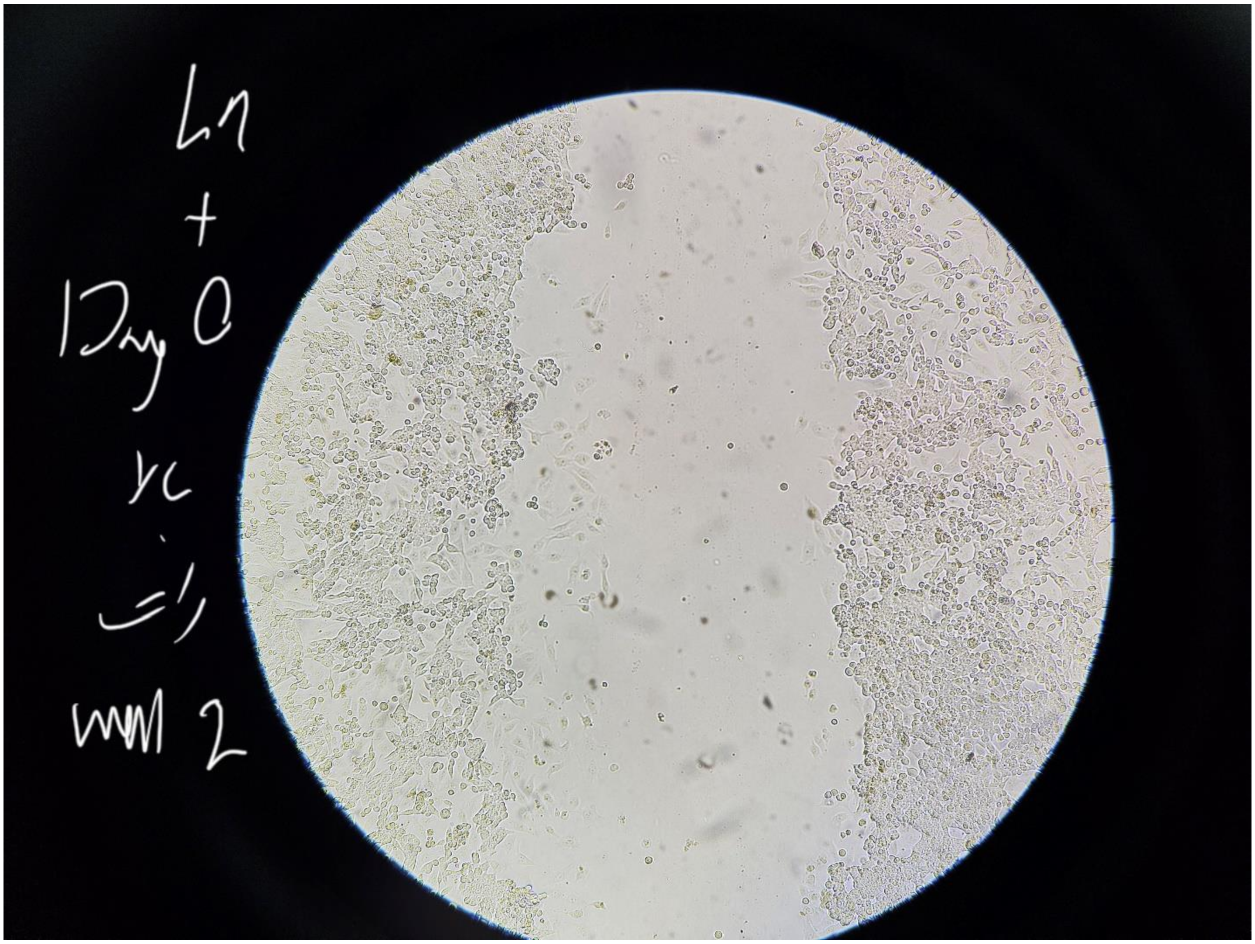

Figure 5E:

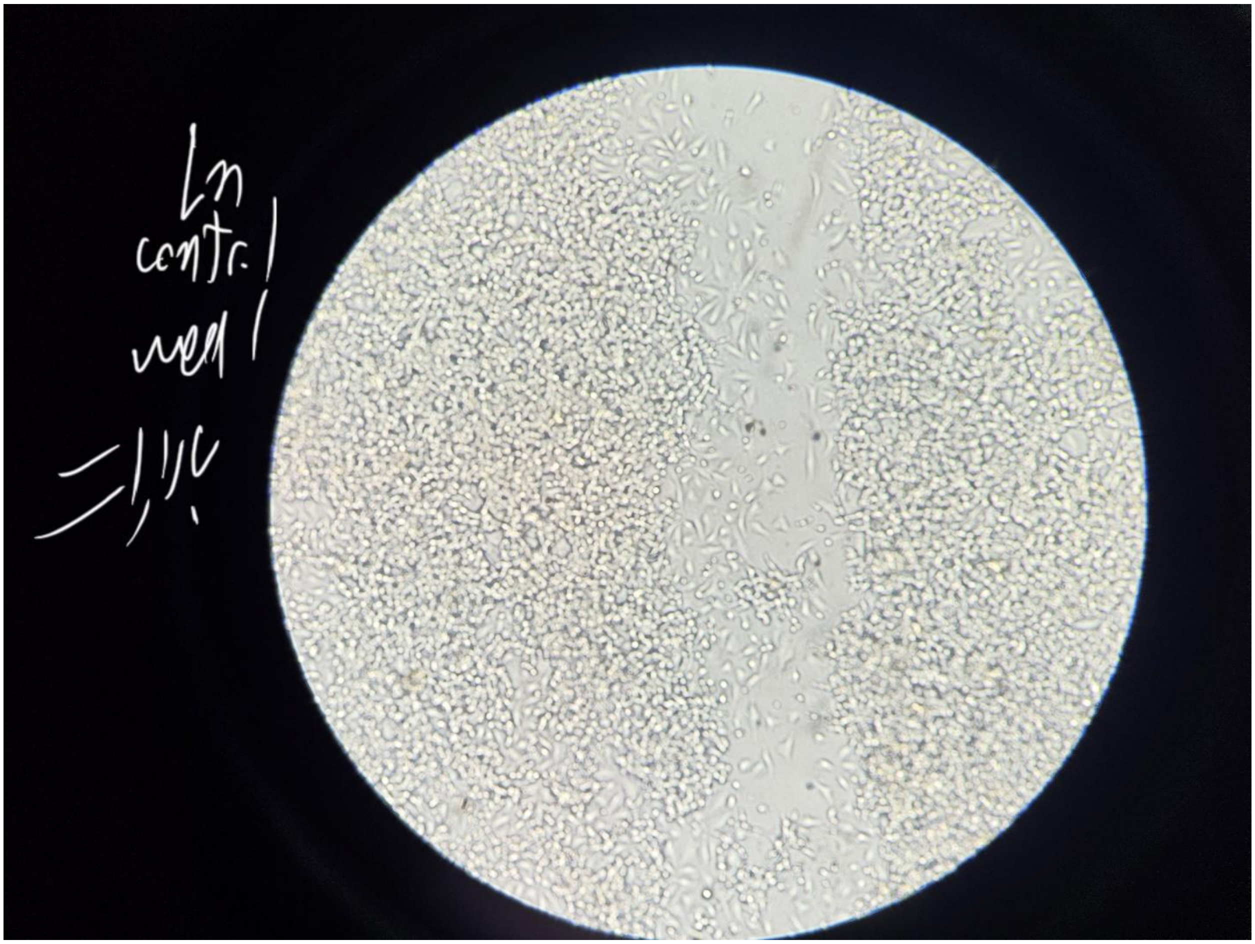

Figure 5F:

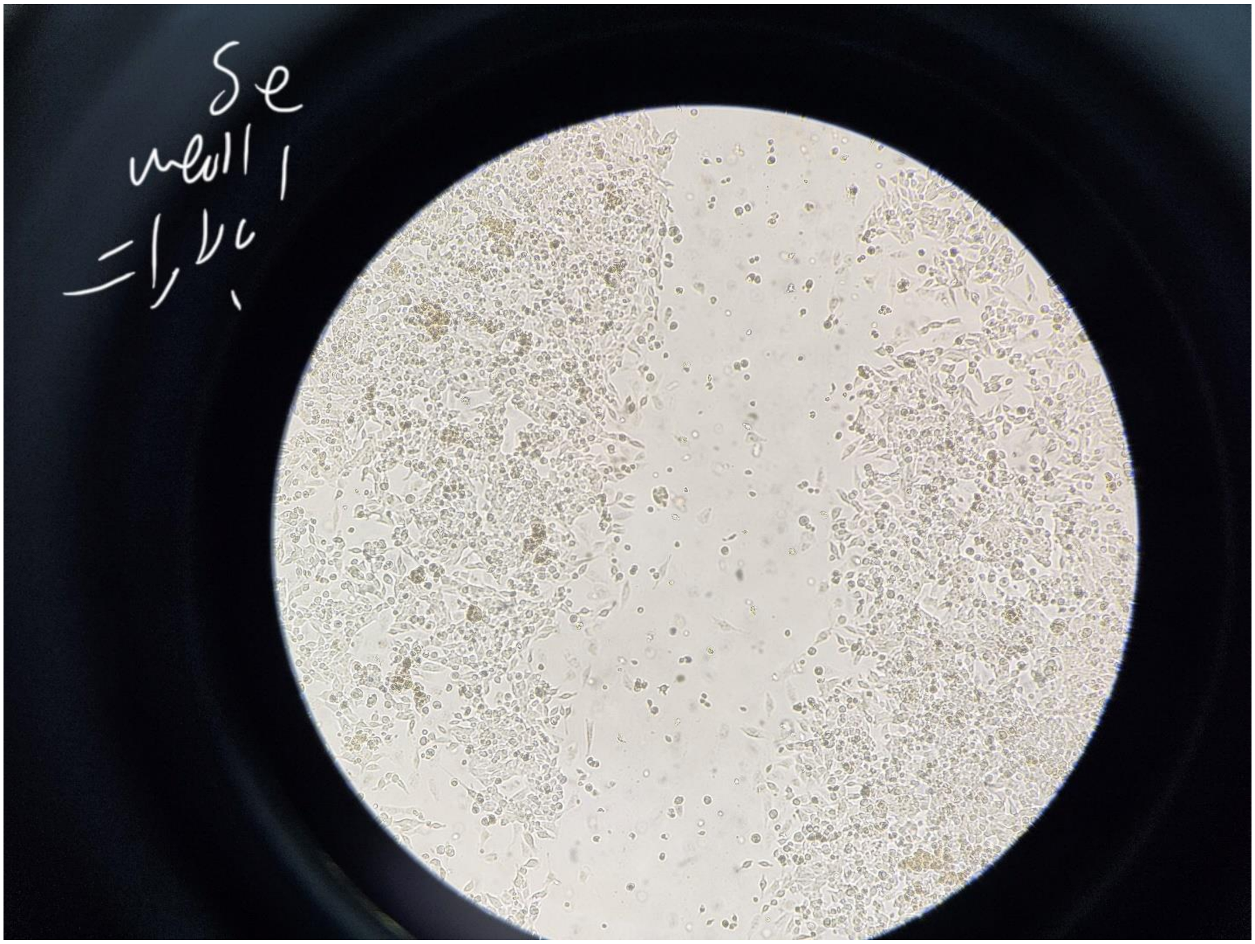

Figure 5G:

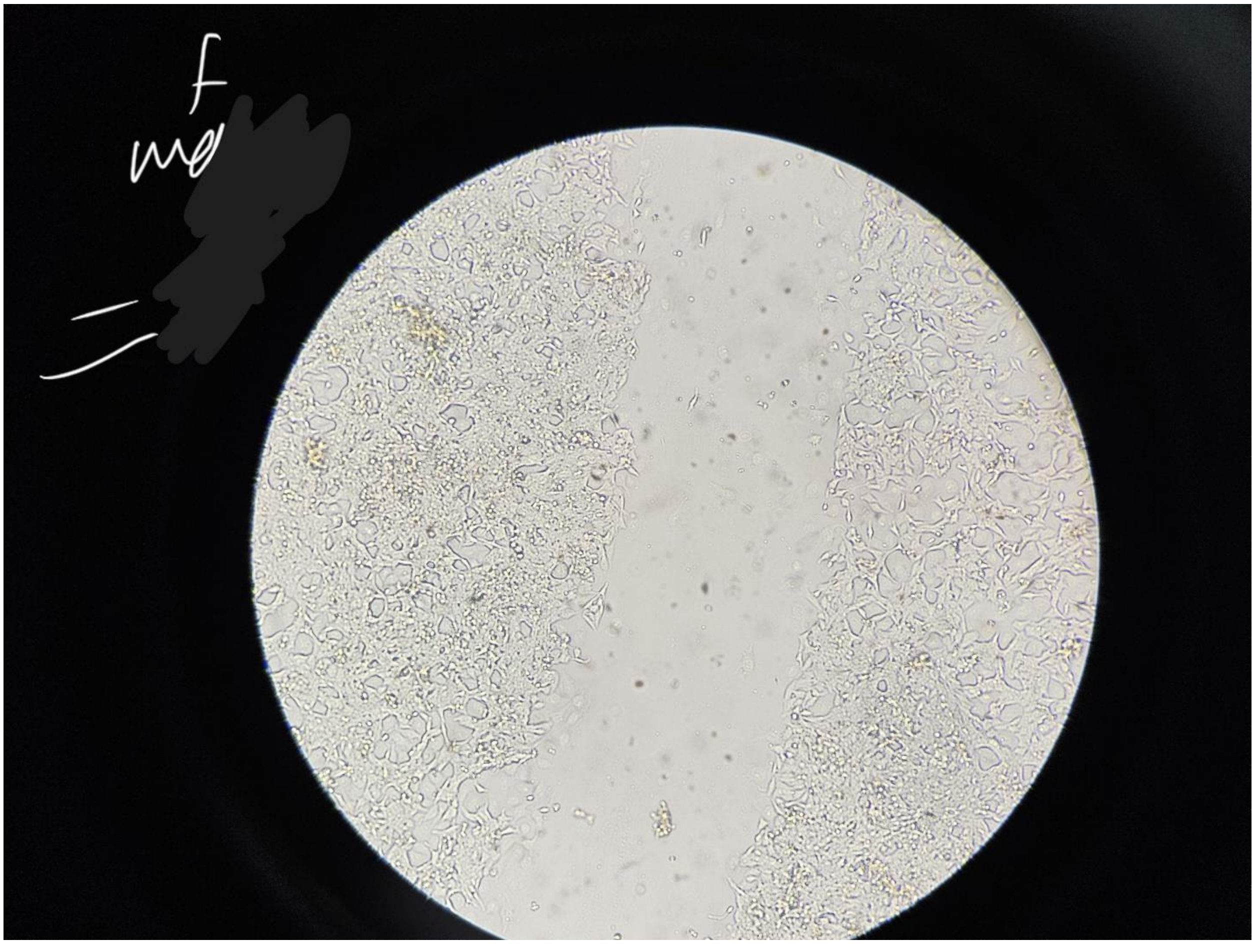

Figure 5H:

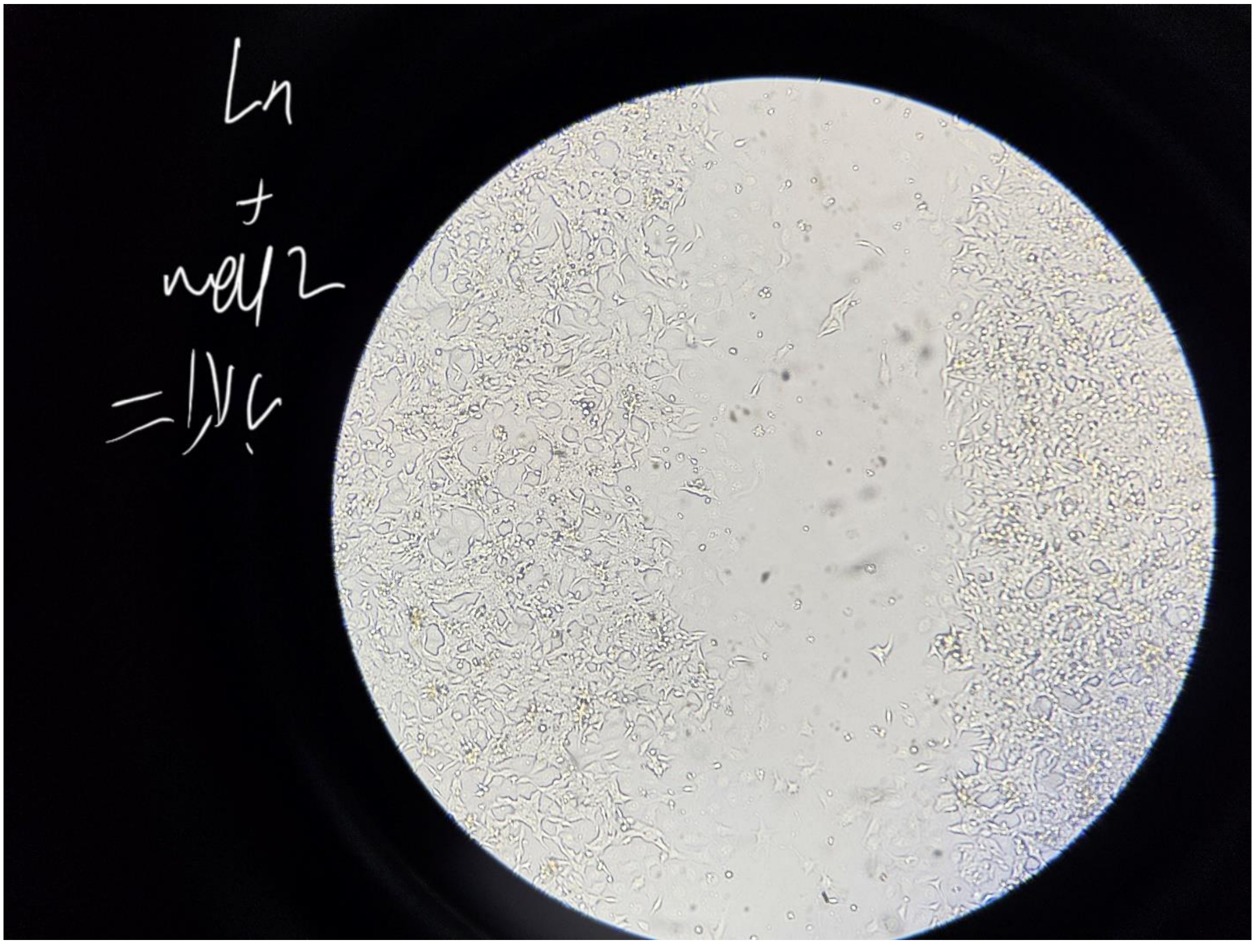

Figure 6A:

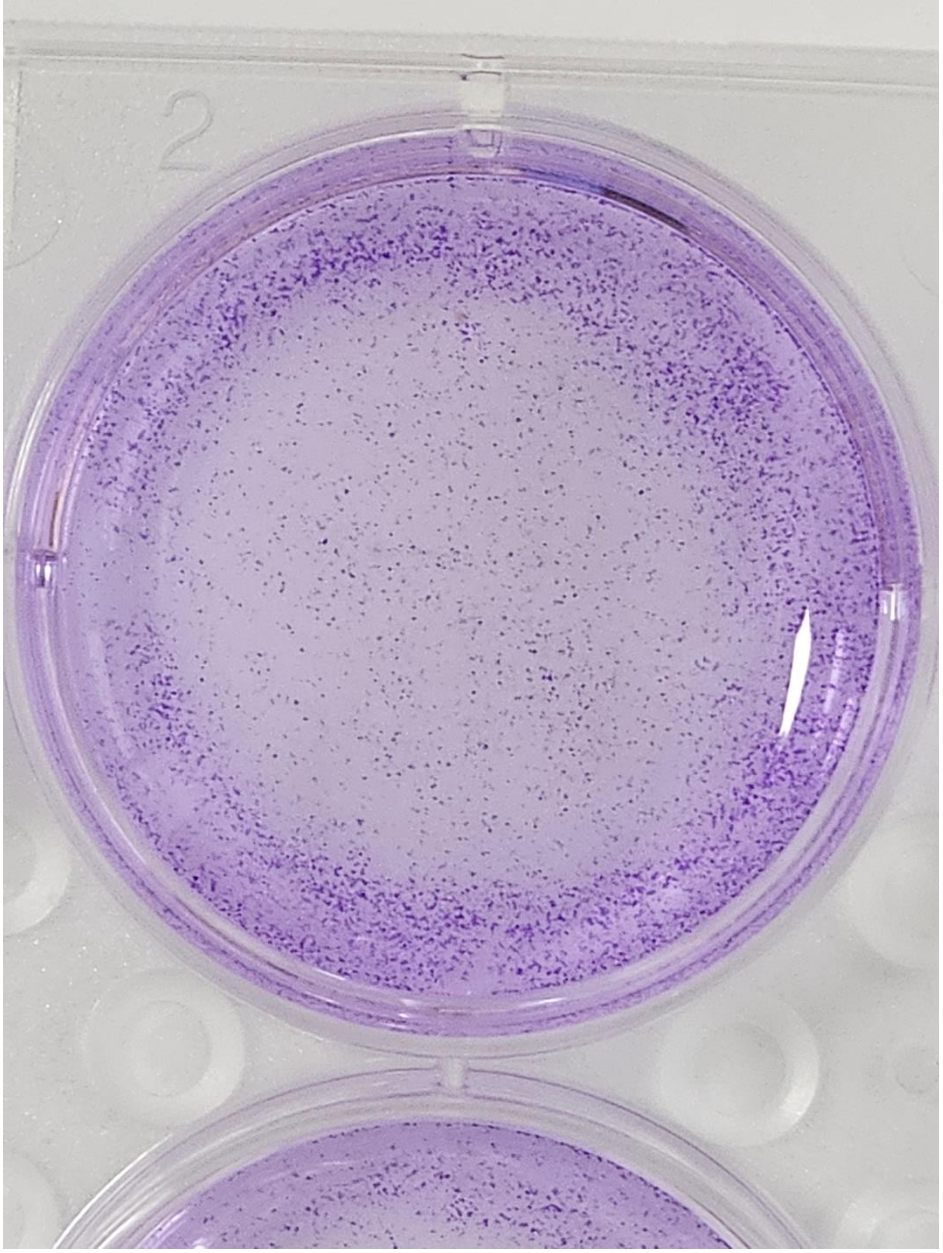

Figure 6B:

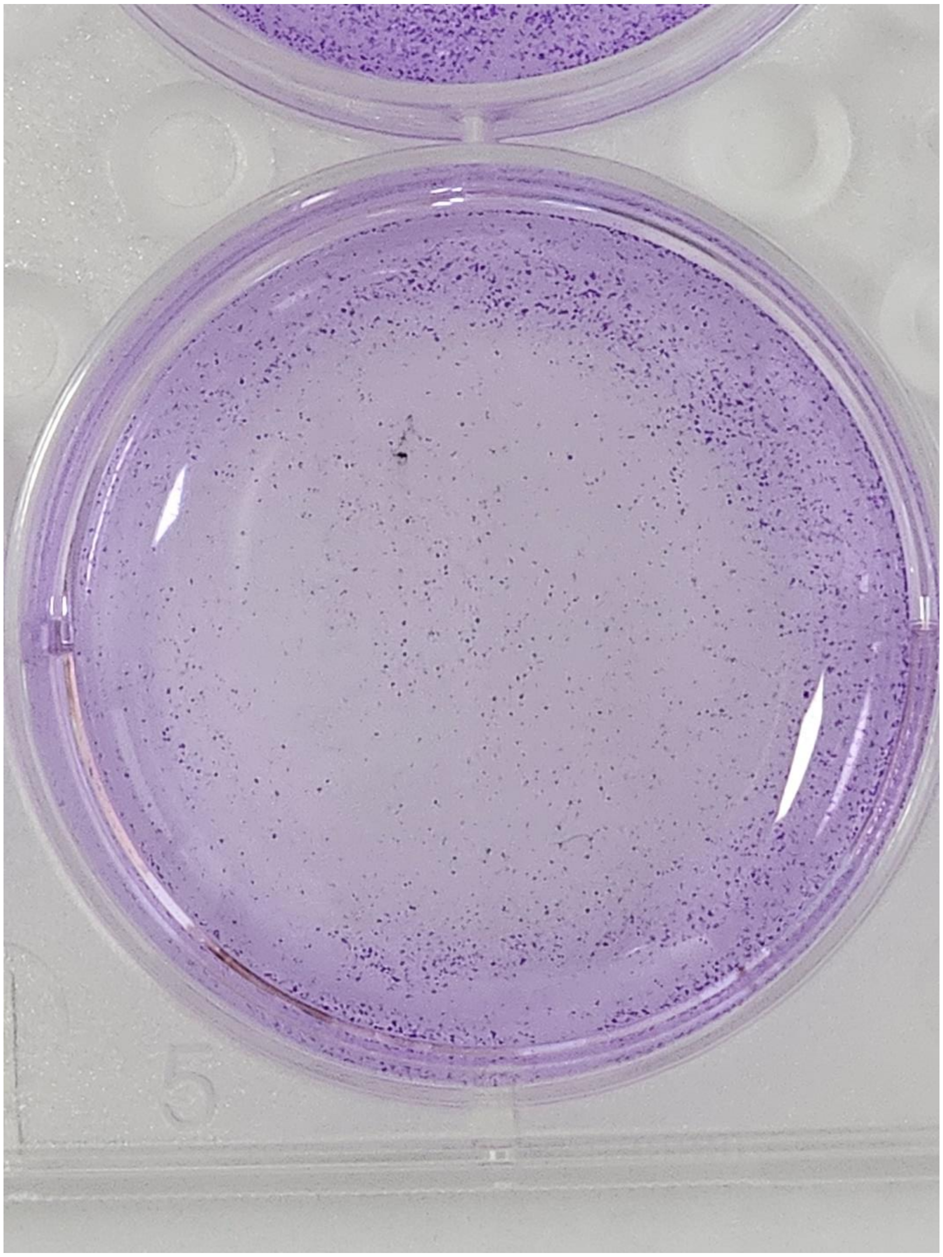

Figure 6C:

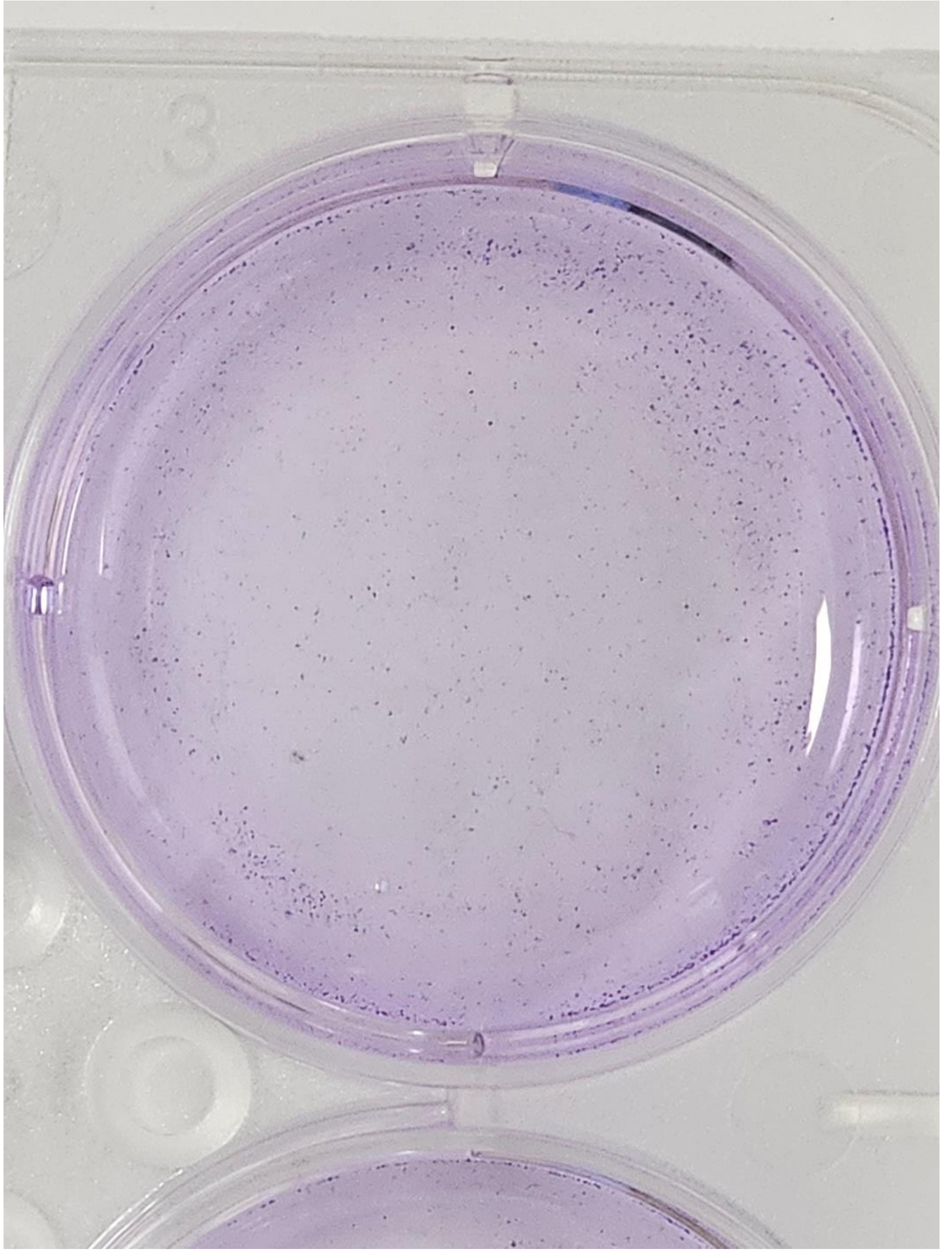

Figure 6D:

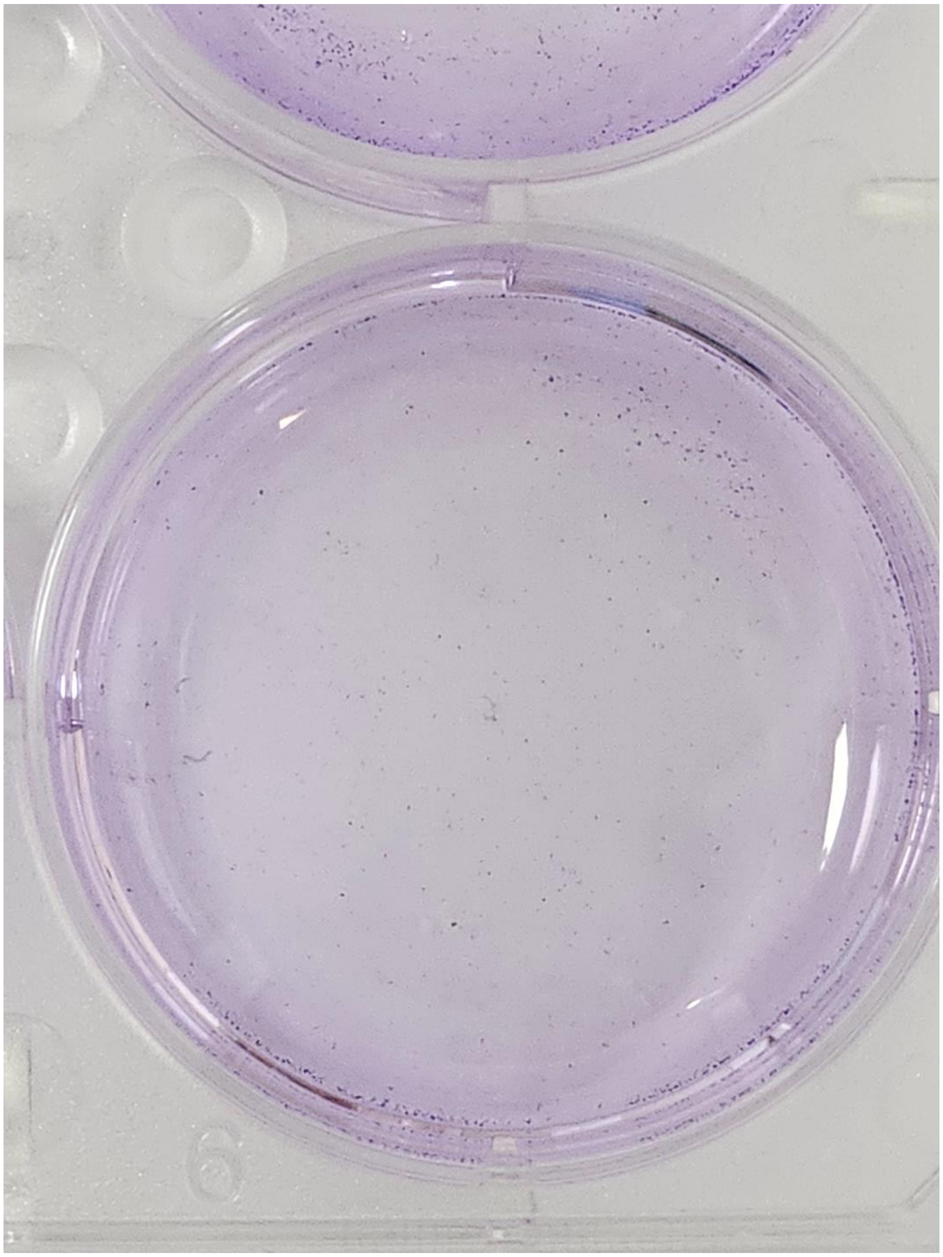

Figure 7A:

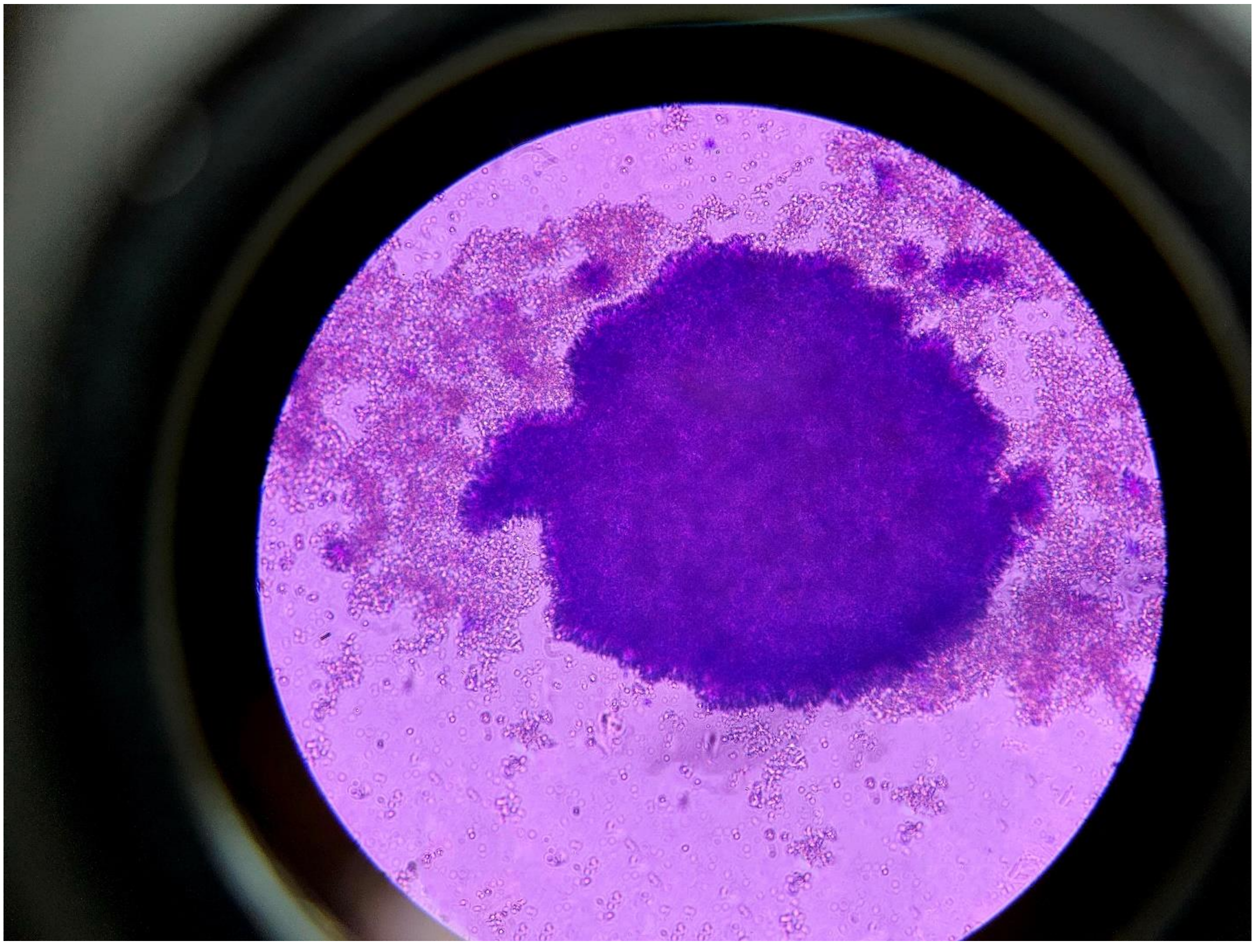

Figure 7B:

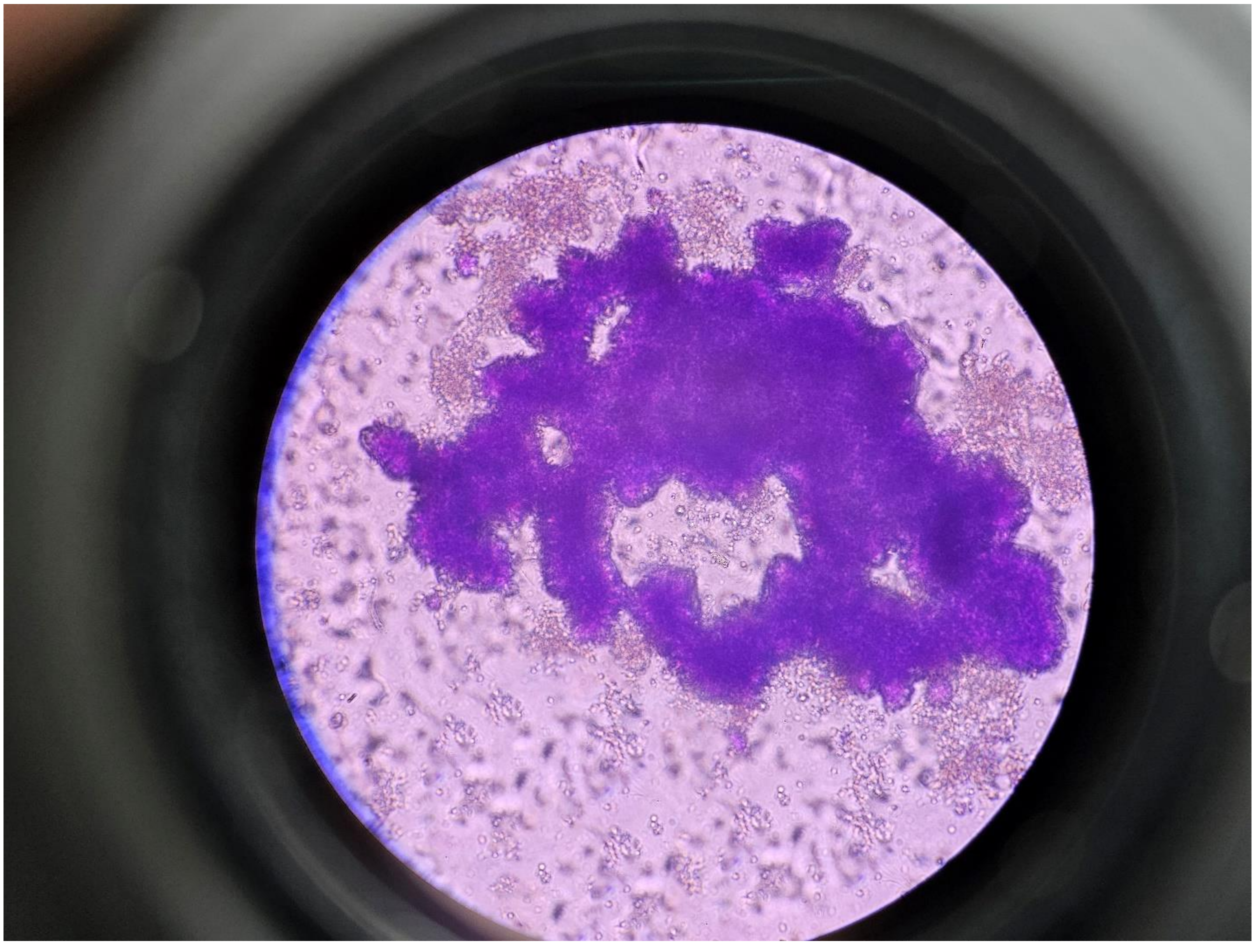

Figure 7C:

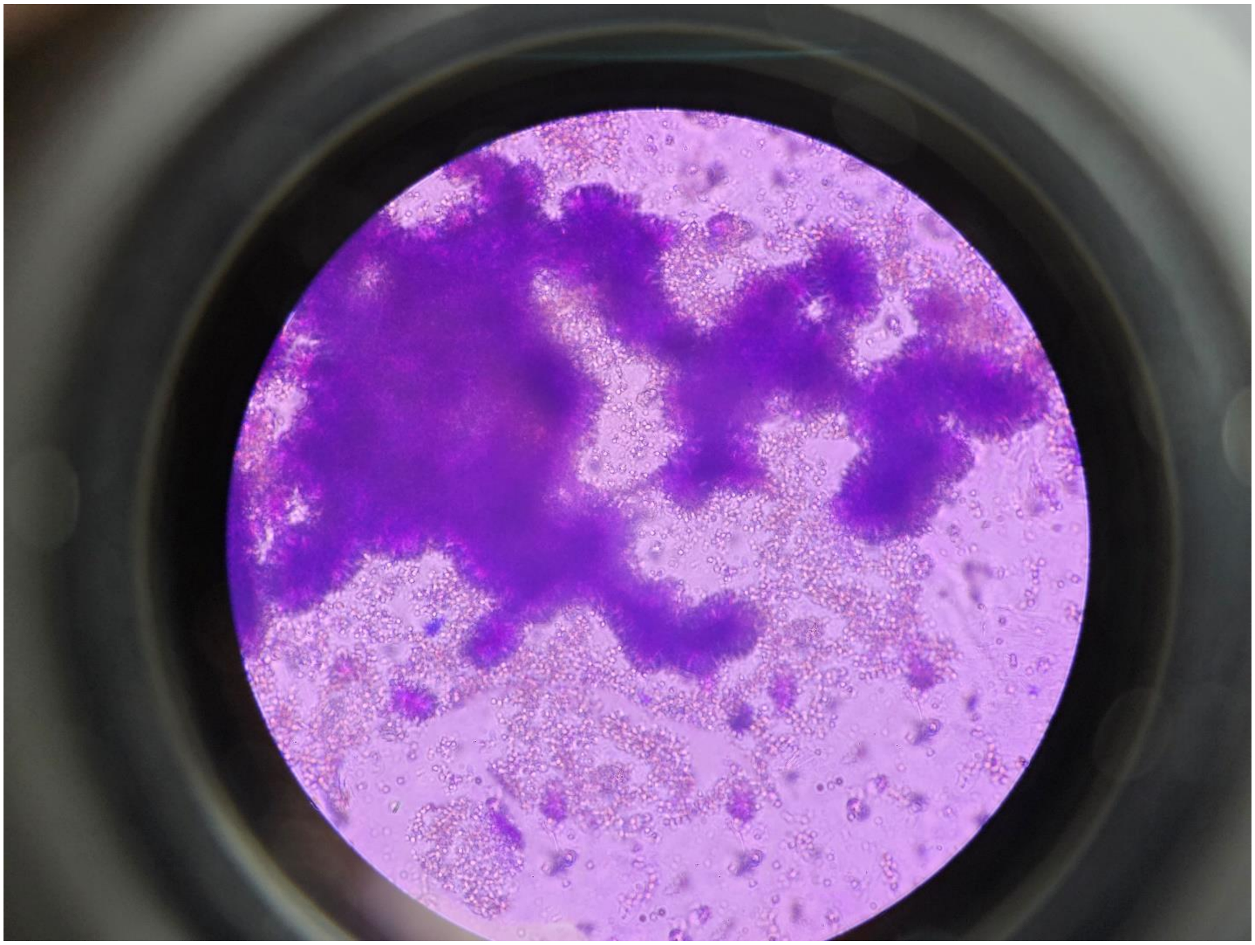

Figure 7D:

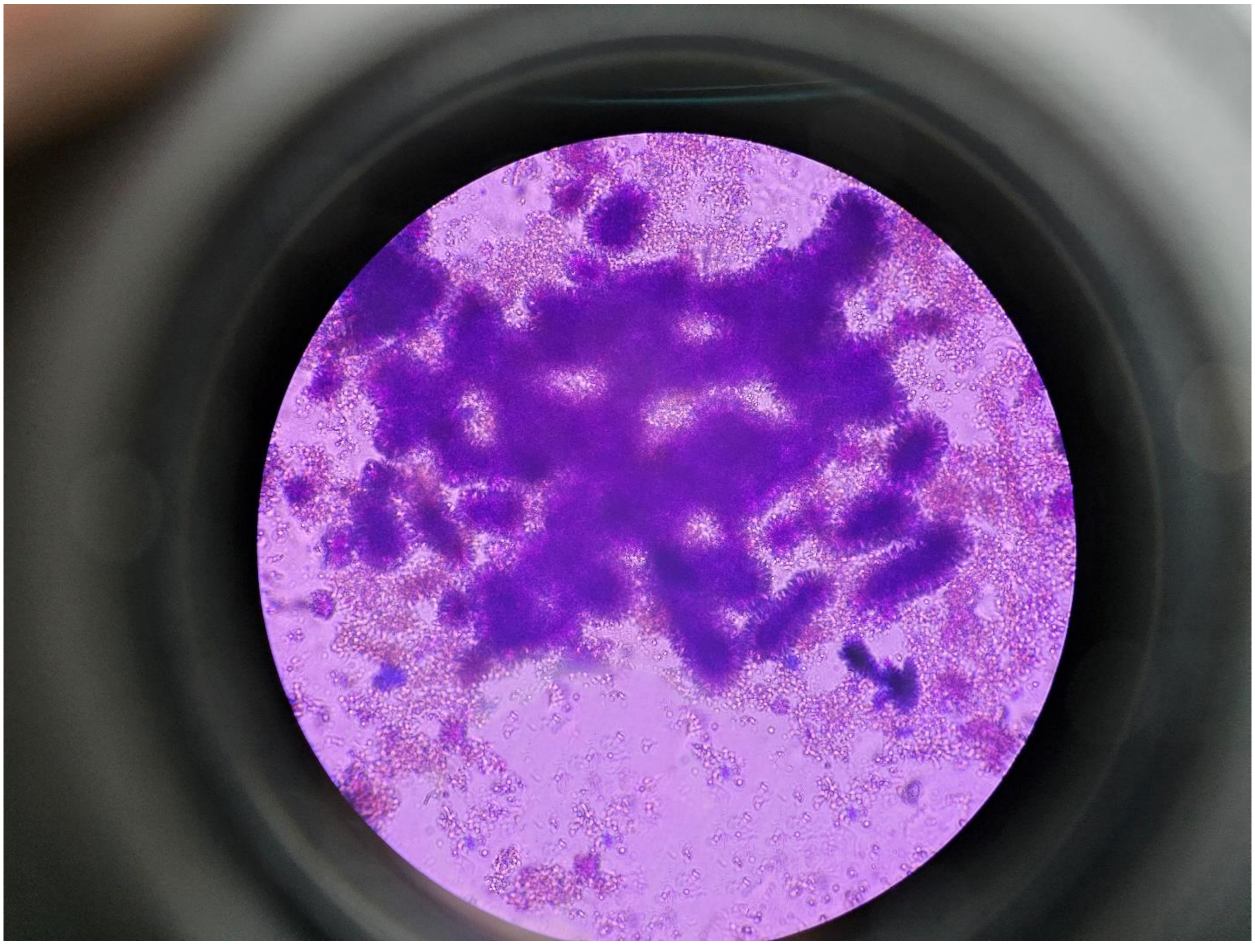

Supplement: S1 Raw Image — The original unprocessed images captured during the experiments, presented without any modifications or enhancements. These images serve as direct visual representations of the experimental results. (PDF) [file pone.0318483.s003.pdf]
