# Supplementary material for: Reducing the effective dosage of flutamide on prostate cancer cell lines through combination with selenium nanoparticles: An in-vitro study
Source: PLoS One. 2025 May 19;20(5):e0318483. doi: 10.1371/journal.pone.0318483 (PMC12088047; doi:10.1371/journal.pone.0318483)
Supplement: Attachment — Submitted filename: PONE-D-24-41345_reviewer.pdf [file pone.0318483.s004.pdf]

PLOS ONE

# Reducing the Effective Dosage of Flutamide on Prostate Cancer Cell Lines through Combination with Selenium Nanoparticles: An In-Vitro Study

--Manuscript Draft--

| | |
|---|---|
| **Manuscript Number:** | PONE-D-24-41345 |
| **Article Type:** | Research Article |
| **Full Title:** | Reducing the Effective Dosage of Flutamide on Prostate Cancer Cell Lines through Combination with Selenium Nanoparticles: An In-Vitro Study |
| **Short Title:** | Reducing the Effective Dosage of Flutamide on Prostate Cancer |
| **Corresponding Author:** | Seyed Mohammad Kazem kazem aghamir<br>Tehran University of Medical Sciences<br>Tehran, Tehran IRAN, ISLAMIC REPUBLIC OF |
| **Keywords:** | prostate cancer- Selenium nanoparticles- flutamide- hepatotoxicity- combination therapy |
| **Abstract:** | Objective<br><br>Objective of the study was to evaluate the therapeutic potential of selenium nanoparticles (SeNPs) in combination with flutamide for treating prostate cancer (PCa) cell lines. The goal was to reduce the dosage of flutamide to decrease its side effects, especially hepatotoxicity.<br><br>Materials and method<br><br>PC3, LnCAP, and DU145 cell lines were treated with varying concentrations of SeNPs and Flutamide to determine IC50 values using the MTT assay. Subsequently, the IC50 concentration of flutamide was reduced by 50% and different concentrations of SeNPs were added to determine new IC50 concentrations of the combinations. Annexin-V/ PI staining was performed to assess the apoptosis rate. The DNA cell cycle was analyzed using the PI staining technique. Migration, proliferative capability, and nucleus morphology of the cells were evaluated through the scratch-wound assay, colony-forming assay, and Hoechst staining, respectively. The expression of SNAIL, KLK3, E-cadherin, VEGF-C, HIF-1α, Bcl2, and BAX were examined using real-time PCR.<br><br>Results<br><br>All treated groups significantly increased early and late apoptosis rate of the PCa cell lines, and induced SubG1/G1 arrest in the cell cycle assay, compared to the control group. Significant inhibition of migration potential and colony formation was observed in all treated groups. Our results suggest that the combination group (50% decrease of Flutamide dosage) treatment upregulated apoptosis-related genes and KLK3, and downregulated genes involved in angiogenesis and proliferation similar to Flutamide alone ($p>0.05$).<br><br>Conclusion<br><br>It is suggested that simultaneous administration of SeNPs and flutamide could potentially reduce the effective dosage of flutamide and decrease its adverse effects. |
| **Order of Authors:** | Iman Menbari Oskouie |
| | Fatemeh Khatami |
| | Amin Shiralizadeh Dezfuli |
| | Rahil Mashhadi |
| | Akram Mirzaei |
| | Seyedeh Negin Hashemi Dougaheh |
| | |

Powered by Editorial Manager® and ProduXion Manager® from Aries Systems Corporation

| | |
|---|---|
| | Helia Azodian Ghajar |
| | Ramin Heshmat |
| | Seyed Mohammad Kazem Aghamir |
| **Additional Information:** | |
| **Question** | **Response** |
| **Financial Disclosure**<br><br>Enter a financial disclosure statement that describes the sources of funding for the work included in this submission. Review the submission guidelines for detailed requirements. View published research articles from *PLOS ONE* for specific examples.<br><br>This statement is required for submission and **will appear in the published article** if the submission is accepted. Please make sure it is accurate.<br><br>**Funded studies**<br>Enter a statement with the following details:<br>• Initials of the authors who received each award<br>• Grant numbers awarded to each author<br>• The full name of each funder<br>• URL of each funder website<br>• Did the sponsors or funders play any role in the study design, data collection and analysis, decision to publish, or preparation of the manuscript?<br><br>Did you receive funding for this work? | The author(s) received no specific funding for this work. |
| **Competing Interests**<br><br>Use the instructions below to enter a competing interest statement for this submission. On behalf of all authors, disclose any competing interests that could be perceived to bias this work—acknowledging all financial support and any other relevant financial or non-financial competing interests.<br><br>This statement is required for submission | There is no competing interest. |

| | |
|---|---|
| and **will appear in the published article** if the submission is accepted. Please make sure it is accurate and that any funding sources listed in your Funding Information later in the submission form are also declared in your Financial Disclosure statement.<br><br>View published research articles from *PLOS ONE* for specific examples.<br><br>**NO authors have competing interests**<br><br>Enter: *The authors have declared that no competing interests exist.*<br><br>**Authors with competing interests**<br><br>Enter competing interest details beginning with this statement:<br><br>*I have read the journal's policy and the authors of this manuscript have the following competing interests: [insert competing interests here]*<br><br>* typeset | |
| **Ethics Statement**<br><br>Enter an ethics statement for this submission. This statement is required if the study involved:<br><br>• Human participants<br>• Human specimens or tissue<br>• Vertebrate animals or cephalopods<br>• Vertebrate embryos or tissues<br>• Field research<br><br>Write "N/A" if the submission does not require an ethics statement.<br><br>General guidance is provided below. Consult the submission guidelines for detailed instructions. **Make sure that all information entered here is included in the Methods section of the manuscript.** | Tehran University of Medical Sciences |

| | |
|---|---|
| **Format for specific study types**<br><br>**Human Subject Research (involving human participants and/or tissue)**<br>• Give the name of the institutional review board or ethics committee that approved the study<br>• Include the approval number and/or a statement indicating approval of this research<br>• Indicate the form of consent obtained (written/oral) or the reason that consent was not obtained (e.g. the data were analyzed anonymously)<br><br>**Animal Research (involving vertebrate animals, embryos or tissues)**<br>• Provide the name of the Institutional Animal Care and Use Committee (IACUC) or other relevant ethics board that reviewed the study protocol, and indicate whether they approved this research or granted a formal waiver of ethical approval<br>• Include an approval number if one was obtained<br>• If the study involved *non-human primates*, add *additional details* about animal welfare and steps taken to ameliorate suffering<br>• If anesthesia, euthanasia, or any kind of animal sacrifice is part of the study, include briefly which substances and/or methods were applied<br><br>**Field Research**<br><br>Include the following details if this study involves the collection of plant, animal, or other materials from a natural setting:<br>• Field permit number<br>• Name of the institution or relevant body that granted permission | |
| **Data Availability**<br><br>Authors are required to make all data underlying the findings described fully available, without restriction, and from the time of publication. PLOS allows rare exceptions to address legal and ethical concerns. See the PLOS Data Policy and FAQ for detailed information. | Yes - all data are fully available without restriction |

| | |
|---|---|
| A Data Availability Statement describing where the data can be found is required at submission. Your answers to this question constitute the Data Availability Statement and **will be published in the article**, if accepted.<br><br>**Important:** Stating 'data available on request from the author' is not sufficient. If your data are only available upon request, select 'No' for the first question and explain your exceptional situation in the text box.<br><br>Do the authors confirm that all data underlying the findings described in their manuscript are fully available without restriction? | |
| **Describe where the data may be found in full sentences. If you are copying our sample text, replace any instances of XXX with the appropriate details.**<br><br>• If the data are **held or will be held in a public repository**, include URLs, accession numbers or DOIs. If this information will only be available after acceptance, indicate this by ticking the box below. For example: *All XXX files are available from the XXX database (accession number(s) XXX, XXX.)*.<br>• If the data are all contained **within the manuscript and/or Supporting Information files**, enter the following: *All relevant data are within the manuscript and its Supporting Information files.*<br>• If neither of these applies but you are able to provide **details of access elsewhere**, with or without limitations, please do so. For example:<br><br>*Data cannot be shared publicly because of [XXX]. Data are available from the XXX Institutional Data Access / Ethics Committee (contact via XXX) for researchers who meet the criteria for access to confidential data.*<br><br>*The data underlying the results presented in the study are available from (include the name of the third party* | no |

| | |
|---|---|
| *and contact information or URL).*<br>• This text is appropriate if the data are owned by a third party and authors do not have permission to share the data.<br><br>* typeset | |
| Additional data availability information: | |

Click here to access/download;Manuscript;SeNPs+Flu-Manuscript-3.docx

# Reducing the Effective Dosage of Flutamide on Prostate Cancer Cell Lines through Combination with Selenium Nanoparticles: An In-Vitro Study

Iman Menbari Oskouie[1], Fatemeh Khatami[1], Amin Shiralizadeh Dezfuli[2], Rahil Mashhadi[1], Akram Mirzaei[1], Seyedeh Negin Hashemi Dougaheh[1], Helia Azodian Ghajar[1], Ramin Heshmat[3]*, Seyed Mohammad Kazem Aghamir[1]*

[1]*Urology Research Center, Tehran University of Medical Sciences, Tehran, Iran*
[2]*Ronash Technology Pars Company (AMINBIC), Tehran University Science and Technology Park, North Campus of Tehran University*
[3]*Chronic Diseases Research Center, Endocrinology and Metabolism Population Sciences Institute, Tehran University of Medical Sciences, Tehran, Iran*

***Corresponding authors**

**1- Seyed Mohammad Kazem Aghamir**
*Tel: (+9821) 6634 8560*
*Fax: (+9821) 6634 8561*
*Email: mkaghamir@tums.ac.ir*
***Mailing Address:*** *Urology Research Center, Sina Hospital, Hassan Abad Sq., Imam Khomeini Ave., Tehran, Iran*

**2- Ramin Heshmat**
*Email:* rheshmat@tums.ac.ir
***Mailing Address:*** Chronic Diseases Research Center, Endocrinology and Metabolism Population Sciences Institute, Tehran University of Medical Sciences, NO 10, Jalale-Al-Ahmad Ave, Chamran Highway, Tehran 1411713137, Iran

## Abstract

**Objective:** Objective of the study was to evaluate the therapeutic potential of selenium nanoparticles (SeNPs) in combination with flutamide for treating prostate cancer (PCa) cell lines. The goal was to reduce the dosage of flutamide to decrease its side effects, especially hepatotoxicity.

**Materials and method:** PC3, LnCAP, and DU145 cell lines were treated with varying concentrations of SeNPs and Flutamide to determine IC50 values using the MTT assay. Subsequently, the IC50 concentration of flutamide was reduced by 50% and different concentrations of SeNPs were added to determine new IC50 concentrations of the combinations. Annexin-V/ PI staining was performed to assess the apoptosis rate. The DNA cell cycle was analyzed using the PI staining technique. Migration, proliferative capability,

and nucleus morphology of the cells were evaluated through the scratch-wound assay, colony-forming assay, and Hoechst staining, respectively. The expression of SNAIL, KLK3, E-cadherin, VEGF-C, HIF-1α, Bcl2, and BAX were examined using real-time PCR.

**Results:** All treated groups significantly increased early and late apoptosis rate of the PCa cell lines, and induced SubG1/G1 arrest in the cell cycle assay, compared to the control group. Significant inhibition of migration potential and colony formation was observed in all treated groups. Our results suggest that the combination group (50% decrease of Flutamide dosage) treatment upregulated apoptosis-related genes and KLK3, and downregulated genes involved in angiogenesis and proliferation similar to Flutamide alone ($p>0.05$).

**Conclusion:** It is suggested that simultaneous administration of SeNPs and flutamide could potentially reduce the effective dosage of flutamide and decrease its adverse effects.

**Keywords:** prostate cancer- Selenium nanoparticles- flutamide- hepatotoxicity- combination therapy

## 1. Introduction

Men between the ages of 45 and 60 are commonly affected by prostate cancer (PCa), which accounts for the majority of cancer-related deaths in Western countries (1). Various methods such as prostate-specific antigen (PSA) testing, digital rectal examination (DRE), magnetic resonance imaging (MRI), or health screening are used to detect PCa in many males (2). Therapeutic modalities for PCa include active surveillance, chemotherapy, radiation therapy, hormone therapy, surgery, and cryotherapy (3).

Androgen-deprivation therapy (ADT), commonly known as hormonal treatment, is suggested for the treatment of recurrent malignancies that have metastasized outside the prostate (1). Flutamide is a nonsteroidal medication that specifically inhibits androgen receptors without any hormonal agonist effects. The combination of flutamide and an LHRH agonist has yielded encouraging outcomes in the treatment of PCa. In in-vivo experiments, flutamide has been demonstrated to act as an antagonist specifically in the ventral prostate and androgen-dependent seminal vesicles (4, 5).

Each derivative of selenium has been documented to possess distinct mechanisms of action that exhibit anticancer properties, with a majority of them specifically noted for their ability to suppress PCa. For example, it has been observed that Selenite induces caspase-mediated death in PC-3 cells, accompanied by DNA breakage, activation of JNK1/2 and p38 MAPK/SAPK2, as well as the formation of mitochondrial superoxide (6, 7). Additionally, it has been documented that it leads to the interruption of the G2/M cell cycle and triggers programmed cell death in HCT116 and SW620 colorectal cancer cells via the Bax-dependent mitochondrial pathway(7). Nevertheless, selenium nanoparticles (SeNPs) have garnered significant interest due to their distinctive optical, magnetic, and structural characteristics that surpass those of molecules or bulk solids (8, 9). Also, SeNPs can serve as carriers for drugs or medicinal substances

in cancer treatment. These nanomaterials have excellent biocompatibility, low toxicity, rapid absorption, and exhibit significant effects (10-14). However, studies have showed that chemically produced nanosized selenium can induce cell cycle arrest, specifically at the S phase, in HeLa cells (15). It also hinders the development of LNCaP cells by reducing the production of androgen receptors in both the gene transcription and protein synthesis sites. furthermore, it activates the phosphorylation and androgen receptors degradation via the Akt/Mdm2 route (16).

Hepatotoxicity, leukopenia, and the growth of medication tolerance are negative impacts that are linked to every PCa therapy (17). While it has become widely recognized that flutamide causes hepatic impairment, an examination on the drug's combination with antiandrogen medication suggested that flutamide would be effective if frequent liver functionality tests were conducted while the patient was receiving treatment (18). Therefore, it is crucial to discover new, affordable chemotherapeutic drugs with little to no side effects and improved effectiveness.

Combined therapy is particularly intriguing for cancer medical care, mainly in males with progressed PCa. We hypothesized that the combination of Flutamide with SeNPs would augment the anticancer potency by concurrently targeting multiple molecular sites. Additionally, it has the potential to result in reduced therapeutic dosages of Flutamide and decrease the hepatotoxicity side effects associated with Flutamide.

Hence, we aimed to assess the combined impact of SeNPs and Flutamide on the growth and programmed cell death, as well as the expression of various genes Such as Snail, KLK3, E-cadherin, and apoptotic genes in three types of prostate cancer cells: LNCaP, PC3, and DU145.

## 2. Materials and method

The study was conducted at the Urology Research Center, Tehran University of Medical Science. subsequent to obtaining REB authorization from the Ethics Review Board of Sina Hospital.

### *2.1. Preparation and characterization of SeNPs*

The compounds, including Sodium selenite ($Na_2SeO_3$, CAS number: 10102-18-8), Polyvinylpyrrolidone (PVP, CAS number: 9003-39-8), and Sodium hydroxide (NaOH, CAS number: 1310-73-2) were acquired from Sigma Aldrich USA. These substances were utilized as obtained, without any further modification. Distilled water was used in this experimental study.

The SeNPs were produced using the subsequent method. 1 g of PVP was blended with a 60 ml solution of distilled water. This mixture was then mixed with 40 ml of a solution containing 100 mM concentration of $Na_2SeO_3$. The pH of the mixture was brought to 7.1 by adding 1.0 M NaOH. The resulting red solution underwent dialysis in double-distilled water for a duration of 72 hours, with water changes every 24 hours. The SeNPs-containing solution was kept at a temperature of 4°C for further investigations.

Transmission electron microscopy (TEM; Zeiss-EM10C) was employed to analyze the size and shape of the SeNPs at an 80 kV accelerating voltage. To obtain absorption spectra in the 200–1000 nm wavelength range, an ultraviolet-visible (UV-vis) spectrophotometer with a 2 nm resolution was used. The SeNPs were also studied through Dynamic Light Scattering (DLS, Particle sizing systems, USA) using a NICOMP 380/ZLS (PSS) system, with the data processed by ZPW388 software.

### *2.2. Cell lines, and cell culture*

The LNCaP (ATCC Number: CRL-10995, NCBI Code: C439), PC3 (ATCC Number: CRL-1435, NCBI Code: C427), and DU145 (ATCC Number: HTB-81, NCBI Code: C428) prostate cancer cell lines were obtained from the National Cell Bank of Pasteur Institute in Tehran, Iran. These cells were cultured in DMEM medium (Gibco, Carlsbad, CA) supplemented with 10% heat-inactivated fetal bovine serum (FBS, Gibco, Carlsbad, CA), 100 units/ml Penicillin, 2 mM L-glutamine, and 100 µg/ml Streptomycin (Gibco BRL, Grand Island, NY). The culturing conditions included a 37°C temperature and a humidified atmosphere containing 5% $CO_2$. A control group was set up using DMEM media with 0.1% dimethyl sulfoxide (DMSO) (Sigma-Aldrich, St. Louis, MO, USA). For the treatment of these cell lines, Flutamide was dissolved in DMSO and sterile phosphate-buffered saline (PBS) to prepare a stock solution.

### *2.3. Analysis of cell survival*

The MTT test was employed to quantify the suppressive impact of Flutamide and SeNPs on the metabolic activity of PC3, LNCaP, and DU145 cell lines. All cell lines were seeded at a density of $5 \times 10^3$ cells per well in 96-well plates and incubated for 24, 48, and 72 hours. Plates were treated with different concentrations of Flutamide (ranging from 3 to 30 µM, with each dosage increasing by 25%) and SeNPs (ranging from 300 to 3000 µM, with each dosage increasing by 25%), both individually. Incubation was carried out at 37 °C with 5% CO2 saturation. To prepare the MTT solution, 5 milligrams of 3-[4,5-dimethylthiazol-2-yl]-2,5 diphenyl tetrazolium bromide powder (Sigma-Aldrich, St. Louis, MO, USA) fully dissolved in 1 milliliter of sterile PBS to obtain a concentration of 5 milligrams per milliliter. Next, dilute with the sterile PBS to a final volume of 10 ml, resulting in a sample solution with a concentration of 0.5 mg/ml. This solution should be freshly prepared for each usage. Subsequently, during a 24, 48, and 72-hour period, 100 µL of the MTT solution (0.5 mg/mL) is introduced to the cells. The cells will then be subjected to a 4-hour incubation at 37 °C to facilitate the metabolization of this solution. Following the disintegration of the formazan crystals in 100 ml of DMSO, a mixture with a purple color will result. An ELISA microplate reader (MPR4+, Hiperion, Medizintechnik GmbH & Co.KG, Germany) was used for measuring the optical density at a range of 570 nm.

For ascertaining the half maximum inhibitory concentration (IC50) of flutamide and SeNPs for the PC3, LNCaP, and DU145 cell lines, the test was carried out triple times. Graphs depicting dose and time responses were created, and the IC50 value, indicative of the concentration that reduces the growth of the control cells by 50%, was calculated using GraphPad PRISM software version 9 (San Diego, CA). The formula for calculating the percentage of cells using the MTT technique is as follows:

$$\frac{\text{(Mean absorbance measured from three replicate drug wells)}}{\text{Mean absorbance obtained from the control wells}} \times 100\% = \%\, cell\ viability$$

After calculating the IC50 concentration of SeNPs and flutamide following 24, 48, and 72 hours of exposure to drugs in various PCa cell lines, the IC50 concentration of flutamide was decreased by 50%, and 75%. Different concentrations of SeNPs were then added to determine the IC50 concentration of flutamide when combined with SeNPs at different times and in different cell lines. This approach, allowed for the identification of these combinations that had a similar inhibitory effect compared to flutamide alone.

### *2.4. Assessing cell Morphology and structure using crystal violet dye.*

$5 \times 10^4$ prostate cancer cells (LNCaP, PC3, and DU145) were seeded into six-well plates following a forty-eight-hour treatment with IC50 concentrations of Flutamide, SeNPs, and their combinations. The cell lines

were rinsed two times with PBS and subsequently treated with Paraformaldehyde for fixation. Afterwards, the cells were stained using a 0.5% w/v solution containing violet crystal. Cell morphology was assessed employing an inverted microscope.

### *2.5. In vitro 2D colony formation assay*

To determine whether prostate cancer cells in a cell medium used for cultivation were invasive, a Colony Forming Test was conducted. In a six-well growing medium, LNCaP, PC3, and DU145 prostate cancer cell lines were grown at a density of 1000 cells per well. Following a 48-hour incubation period, the cells were exposed to IC50 doses of Flutamide, SeNPs, and their combinations. The plate was then placed in an incubator at a temperature of 37°C until cells developed into colonies that could be seen. After a period of 14 days, the culture medium was rinsed with PBS solution two times. Subsequently, the colony cells were exposed to a 0.5% w/v crystal violet solution for a duration of 30 minutes at a temperature of 25°C. A colony was defined as consisting of 50 or more cells, while a cluster consisted of 3-50 cells. The experiments were repeated three times. Photographs were taken of each well, and the quantity of colonies in every image was counted and assessed utilizing ImageJ software.

### *2.6. Staining of cells with Hoechst dye (33342)*

An assessment of apoptosis in cancer cells was conducted utilizing a Hoechst dye test. Prostate cancer cells were seeded into a 96-well plate at a density of $2.5\times10^3$ cells per well. They were then treated with Flutamide, SeNPs, and a combination of both. After a 48-hour incubation period, the cells were fixed with 100 µL of cold Paraformaldehyde (4%) and subsequently rinsed two times with 100 µL of PBS. Next, a volume of 2 µL of Hoechst dye was introduced to the cell pellet and incubated at a temperature of 25 °C, while maintaining darkness. After 30 minutes, the sample was examined using a fluorescence microscope at 100X magnification to detect nuclear condensation and count the number of broken nuclei in intact cells. Apoptosis was identified by the presence of fragmented nuclei in certain cells.

### *2.7. Measurement of cell migration*

In a six-well culture plate, LNCaP, PC3, and DU145 prostate cancer cell lines were grown at a density of $5\times10^5$ cells per well. Once the cells attained a density of 85%, a sampler tip was used to construct a vertical path across the diameter of the six cells. In order to eliminate the individual cells, the lower part of the incubation plate was delicately rinsed with PBS. Afterward, certain cells were subjected to PBS in order to serve as a control group, while others were treated with drugs. All cell lines received IC50 doses of flutamide, SeNPs, and a combination of both. Subsequently, imaging was conducted at 0 and 48 hours intervals. The images were analyzed using ImageJ software and wound closure was calculated using the following formula:

$$1-\frac{\text{area of wound at day 2}}{\text{area of wound at day 0}}\times 100\% = \%\, wound\ closure$$

### *2.8. tumor spheroid model*

Plates covered with agar were utilized to produce tumor spheroids. According to Friedrich et al. (19), 50 µl/well of agar (2% (wt/vol) dissolved in RPMI) was applied to 96 well plate. PC3, LnCap, and DU145 cells were seeded in wells with a seeding density of 2000 cells per well and left to cultivate at a temperature of 37 °C in an environment with 5% carbon dioxide. Following a duration of 96 hours, a solitary spheroidal

object with a diameter ranging from 300µm to 400µm had formed within every individual well. The spheroids from all cell lines were then treated with IC50 doses of flutamide, SeNPs, and their combinations. After 48 hours, the colonies were stained with 0.5% w/v crystal violet solution for 30 minutes at 25 °C, followed by two washes with PBS.

### *2.9. Evaluating cell apoptosis through flow cytometry*

The cell apoptosis assessment was conducted utilizing the manufacturer's protocol for the Fluorescein-conjugated annexin V (annexin V-FITC) staining assay. The LNCaP, PC3, and DU145 cell lines were placed in six-well dishes with a population density of 3 × $10^5$ cells per well. Cells were subsequently cultured for 48 hours with and without IC50 doses of SeNPs, Flutamide, and their mixtures. After two rinses with PBS, 100 µl of staining solution comprising PI and annexin-V was added to the medicated and control prostate cancer cell populations. The cells were then incubated for 15 minutes at 25°C in dark conditions. Afterward, flow cytometry was used to evaluate the fluorescent signals. The scatter maps were divided into four sections. The area representing viable cells (annexin-V-negative and PI-negative) was labeled as Q4 and displayed in the lower left corner. The area representing early apoptotic cells (annexin-V-positive and PI-negative) was labeled as Q3 and demonstrated in the lower right quadrant. The region representing late apoptotic cells (annexin-V-positive and PI-positive) was labeled as Q2 and presented in the upper right quadrant. The zone representing necrotic cells (annexin-V-negative and PI-positive) was labeled as Q1 and exhibited in the upper left quadrant. The apoptotic rate was quantified as the proportion of annexin V+/PI- cells using a BD flow cytometer and analyzed with Flowjo software (Tree Star Inc., version 9.6.3, USA).

### *2.10. DNA cell cycle analysis*

Propidium Iodide (PI) staining was used to analyze the cell cycle. PC3, LNCaP, and DU145 cell lines were seeded in 6-well plates, at a population density of 5 × $10^5$ cells per well. The cells were then treated with Flutamide, SeNPs, and their combination at IC50 doses for a duration of 48 hours. After treatment, the cells were rinsed two times with PBS, fixed with 70% cold ethanol, and stored at -20°C overnight. Subsequently, the cells were rinsed with PBS two times and subjected to a 30-minute incubation at 37°C with RNase I (100 µg/ml) and stained DNA with 500 µL PI (50 µg/ml in 0.1% Triton X-100/0.1% sodium citrate). A BD flow cytometer was used to separate the cells. A sample of DNA was assessed by flow cytometry, and the findings were evaluated using the Flowjo program (Tree Star Inc., version 9.6.3, USA). The hypodiploid sub-G0/G1 DNA percentage can be used to determine the apoptotic cell fraction.

### *2.11. RNA extraction, cDNA synthesis and gene expression analysis by real-time PCR*

In adherence to the guidelines provided by the manufacturer, total RNA was isolated utilizing the Highly Pure RNA isolation kit (Roche Applied Science, Germany). Initially, the quantification and purity of total RNA were evaluated by means of spectrophotometry at 260 and 280 nanometers with a Nanodrop ND-1000 (Nanodrop Technologies, Wilmington, DE). Following this, complementary DNAs (cDNAs) were reverse transcribed from 1 to 2 µg of total RNA using the cDNA synthesis PrimeScript RT reagent Kit (Takara Bio Inc., Otsu, Japan). According to the manufacturer’s guideline, the cDNA level was adjusted for normalization through a series of PCR employing B2M primers (Table 1). Subsequently, the normalized cDNAs underwent production utilizing the real-time PCR cycler from QIAGEN. The housekeeping gene B2M was employed to establish normalization of gene expression levels, while the relative expression was

computed using the $2^{-\Delta\Delta CT}$ approach. Table 1 presents the primers and their corresponding amplicon lengths.

Table 1. Nucleotide sequences of primers used for real-time PCR

| Gene | Forward primer (5′ -3′ ) | Reverse primer (5′ -3′ ) |
|---|---|---|
| KLK3 | CGTGACGTGGATTGGTGCT | TTCCTGATGCAGTGGGCAGC |
| E-cadherin | TCGTAACGACGTTGCACCAA | TTCGGAACCGCTTCCTTCAT |
| SNAIL | TAGCGAGTGGTTCTTCTGCG | AGGGCTGCTGGAAGGTAAAC |
| VEGF-C | GCTTCTTCTCTGTGGCGTGT | CTTTGCTTGCATAAGCCGTGG |
| Bcl2 | CCCCGCGACTCCTGATTCAT | CAGTCTACTTCCTCTGTGATGTTGT |
| BAX | CGGGTTGTCGCCCTTTTCTAC | AGTCCAATGTCCAGCCCATGA |
| HIF-1α | GTGCCACATCATCACCATATAG | GCTTTCTCTGAGCATTCTGCAA |
| B2M | TGTCTTTCAGCAAGGACTGGT | TGCTTACATGTCTCGATCCCAC |

### *2.12. Statistical analysis*

The means ± SE of the triplicate determinants were used to display all the data. The data were assessed using ANOVA and t-tests. When compared to the matching control, $*P < 0.05$, $**P < 0.01$, $***P < 0.001$, and $****P < 0.0001$ were considered statistically significant.

## 3. Results

### *3.1. Characterization of SeNPs*

The morphology of the SeNPs was examined through TEM. The SeNPs were spherical in shape and had various particle diameters, displayed in Figure 1A. According to the TEM data, the average diameter of the SeNPs is 45.34nm. The distribution of selenium nanoparticles (depicted as dark spots) was monodispersed and exhibited a homogeneous structure.

Particle size measurements were determined using DLS analysis as shown in Figure 1B. The results revealed that the average values of particle size for SeNPs were 66.61 nm.

The dispersion of SeNPs were analyzed using UV-Vis spectrophotometry, revealing morphological alterations such as variations in nanoparticle dimensions and agglomeration (Figure 1C). It was determined that the development of surface plasmon vibration (SPR) on spherical SeNPs was responsible for the maximum absorption bands situated between 200 and 300 nm (20, 21). The diameter of the SeNPs is directly proportional to the wavelength at which the highest intensity of the SPR band occurs. Additionally, the total concentration of SeNPs is directly related to the content of the absorbance peak. A study found that glucose-stabilized Se nanospheres exhibited an absorption maximum at a wavelength of 295 nm, similar to our study.(22)

.

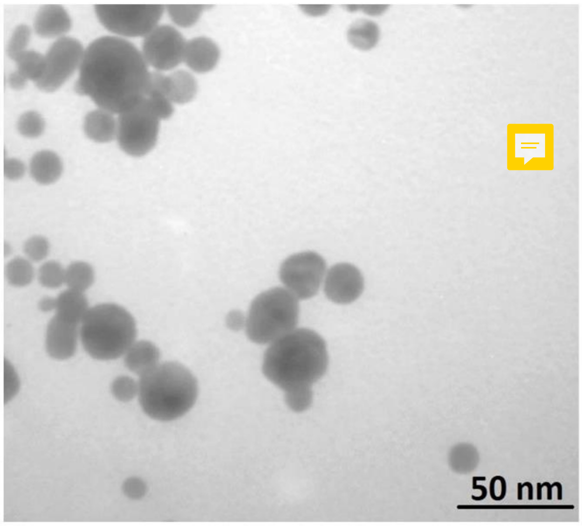


A

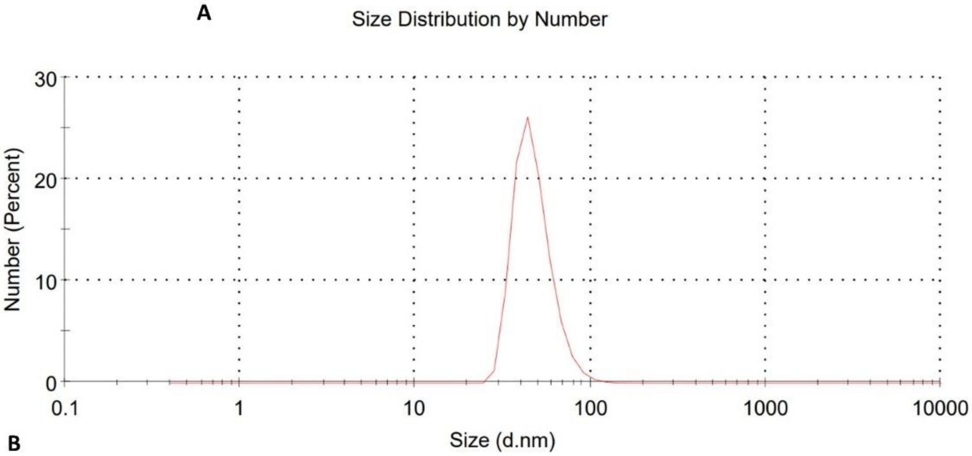


B

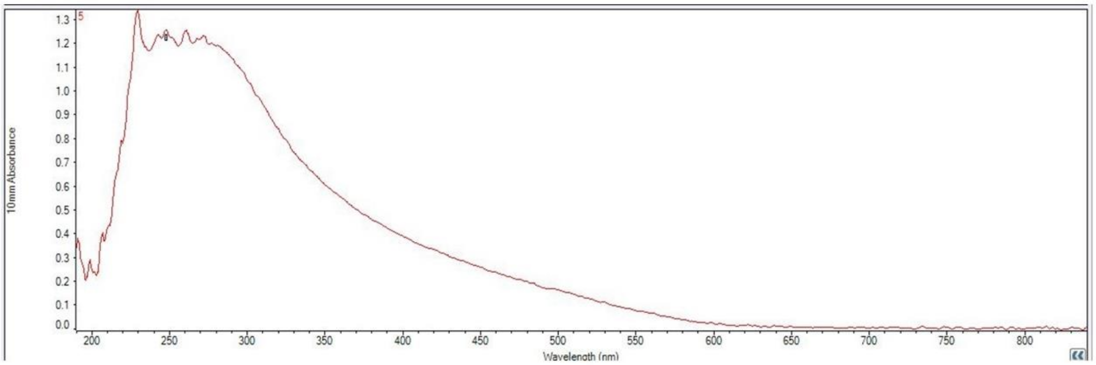


C

Figure 1. (A): TEM image of SeNPs, (B): DLS of SeNPs, (C): UV-Vis spectrum of SeNPs

### *3.2. effect of SeNPs and Flutamide on cell proliferation*

In PC3, LNCap, and DU145 cell lines, the cytotoxic effects of SeNPs and Flutamide were investigated. Based on the results (figure 2.), the IC50 values after 48 hours of treatment for SeNPs were 1788 µM, 1430 µM, and 915 µM for PC3, LNCap, and DU145 cell lines, respectively. The IC50 values after 48 hours of treatment for Flutamide were 17.88 µM, 14.3 µM, and 11.44 µM for PC3, LNCap, and DU145 cell lines, respectively. The results exhibited that SeNPs and Flutamide had an important cytotoxic effect on all three cell lines in a dose-dependent way. To evaluate the synergistic characteristic of SeNPs and Flutamide the viability of treated cells was measured after treating prostate cancer cells with the combination of flutamide and SeNPs as described in the materials and methods section. The new IC50 values for combination therapy (50% decrease in flutamide IC50 concentration) after 48 hours of treatment were 223.5 µM SeNPs + 8.94 µM Flutamide for PC3, 178.8 µM SeNPs + 7.15 µM Flutamide for LNCap, and 114.4 µM SeNPs + 5.72 µM Flutamide for DU145. Additionally, IC50 values for combination therapy (75% decrease in flutamide IC50 concentration) after 48 hours of treatment were 1117 µM SeNPs + 4.47 µM Flutamide for PC3, 894 µM SeNPs + 3.57 µM Flutamide for LNCap, and 715 µM SeNPs + 2.86 µM Flutamide for DU145 (Figure 2). The IC50 concentrations for 48 hours were selected for all subsequent experiments. In the combination treatment group, the synergistic effect of SeNPs and Flutamide was more prominent when a 50% reduction in Flutamide IC50 concentration occurred. Therefore, these concentrations were chosen for combination treatment in the following experiments.

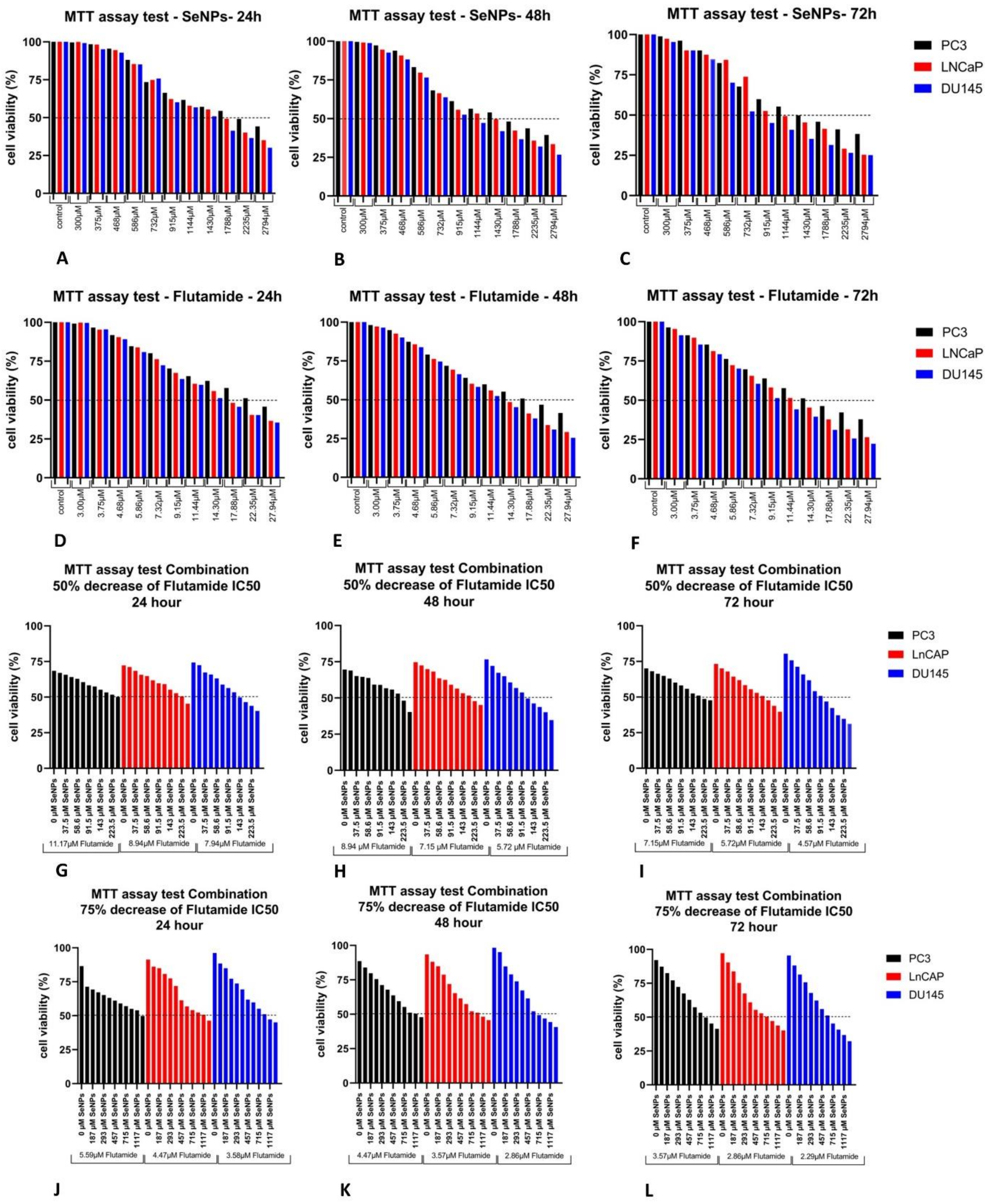


Figure 2. The effect of SeNPs, Flutamide, and their combinations with various concentrations on PC3, LNCap, and DU145 cell proliferation. The anti-growth effect of SeNPs (A-C), Flutamide (D-F), and their combinations (G-L) after 1,2 and 3 days on PC3, LnCAP, and DU145 cell lines was assessed by MTT test.

### *3.3. Morphological changes*

Examining cellular morphology is crucial for comprehending cell attitude. After treating cells with Flutamide, SeNPs, and their combinations for 48 hours, morphological alterations of PC3, LnCAP, and DU145 cell lines were examined using an inverted microscope. As depicted in Figure 3, the cells underwent morphological alterations such as shrinkage, rounding, elongation, membrane protrusion, and a decrease in the number of living cells after treatment with Flutamide, SeNPs, and their combination in comparison to the control group. These structural changes imply that Flutamide and SeNPs have the potential to cause apoptosis in the PC3, LNCap, and DU145 cell lines.

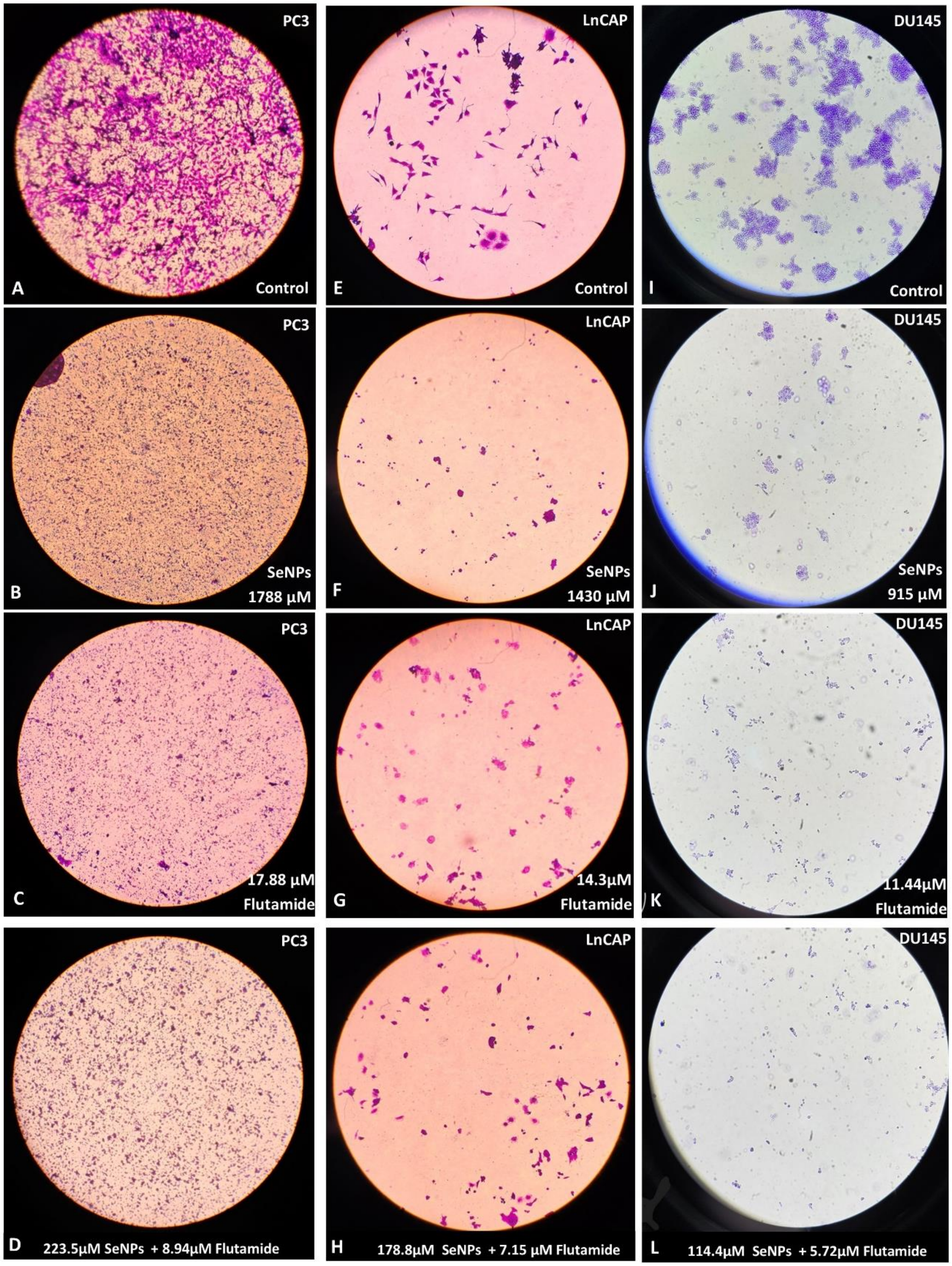


Figure 3. Morphological changes in prostate cell lines after treatment with SeNPs, Flutamide, and their combinations. (A) PC3-control (B) PC3-SeNPs (C) PC3-Flutamide (D) PC3-combination (E) LnCap-control (F) LnCap-SeNPs (G) LnCap-Flutamide (H) LnCap-combination (I) Du145-control (J) Du145-SeNPs (K) Du145-Flutamide (L) Du145-combination

## *3.4. Effects of SeNPs and Flutamide on the nuclei of prostate cancer cell lines*

The PC3, LNCaP, and DU145 cell lines were treated with IC50 doses of Flutamide, SeNPs, and their mixtures for a duration of 48 hours to evaluate apoptosis through fluorescent microscopy and Hochst staining techniques. Outcomes obtained using the Hoechst 33342 fluorescent dye are presented in Figure 4, where the nuclei in the control group showed blue fluorescence. After treatment with SeNPs and Flutamide, significant alterations in the nuclei morphology were seen. Several nuclei split and disintegrated following exposure to their IC50 concentrations of SeNPs, Flutamide, and their combination compared to the control group after 48 hours, resulting in dispersed nuclear contents. Additionally, apoptosis characteristics were seen under a fluorescent microscope (Figure 4).

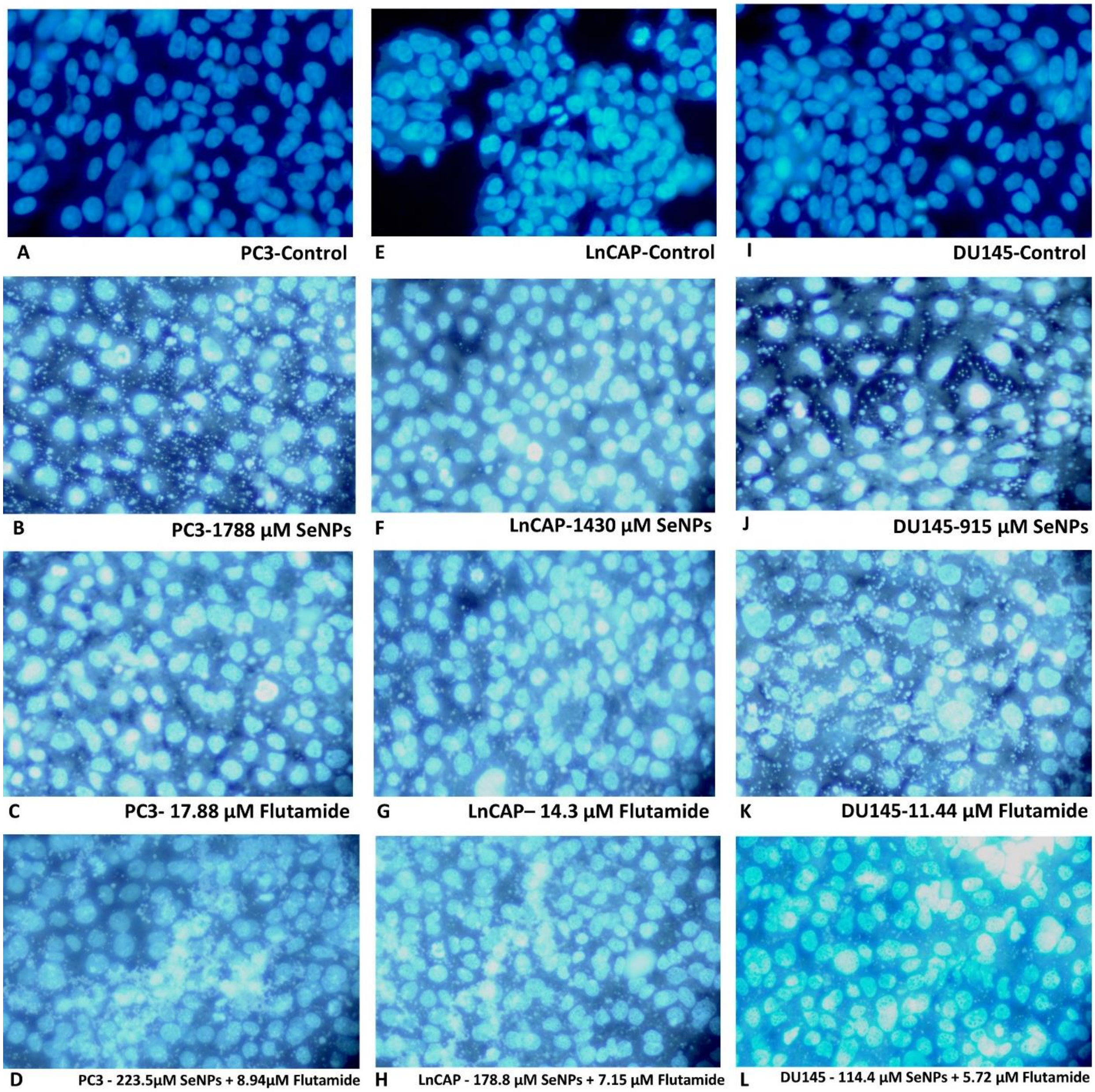

Figure 4. Fluorescent staining with Hoechst dye (33342) of prostate cell line after treating with SeNPs, Flutamide, and their combinations. (A) PC3-control (B) PC3-SeNPs (C) PC3-Flutamide (D) PC3-combination (E) LnCap-control (F) LnCap-SeNPs (G) LnCap-Flutamide (H) LnCap-combination (I) Du145-control (J) Du145-SeNPs (K) Du145-Flutamide (L) Du145-combination. Fragmented nuclei in some cells indicate apoptosis.

### *3.5. Effects of SeNPs and Flutamide on the migration of the prostate cancer cells*

Figure 5 and Supplementary Figures 1 and 2 display the outcomes of the migration assay. As illustrated in Figure 5, the movement of prostate cancer cells was significantly reduced by the treatment with SeNPs, Flutamide, and their mixture after 48 hours, in comparison to the control group. The percentage of wound closure in both the treated and control groups across all cell lines is depicted in Figure 5. Nevertheless, in the control group, the gap between cells has nearly closed.

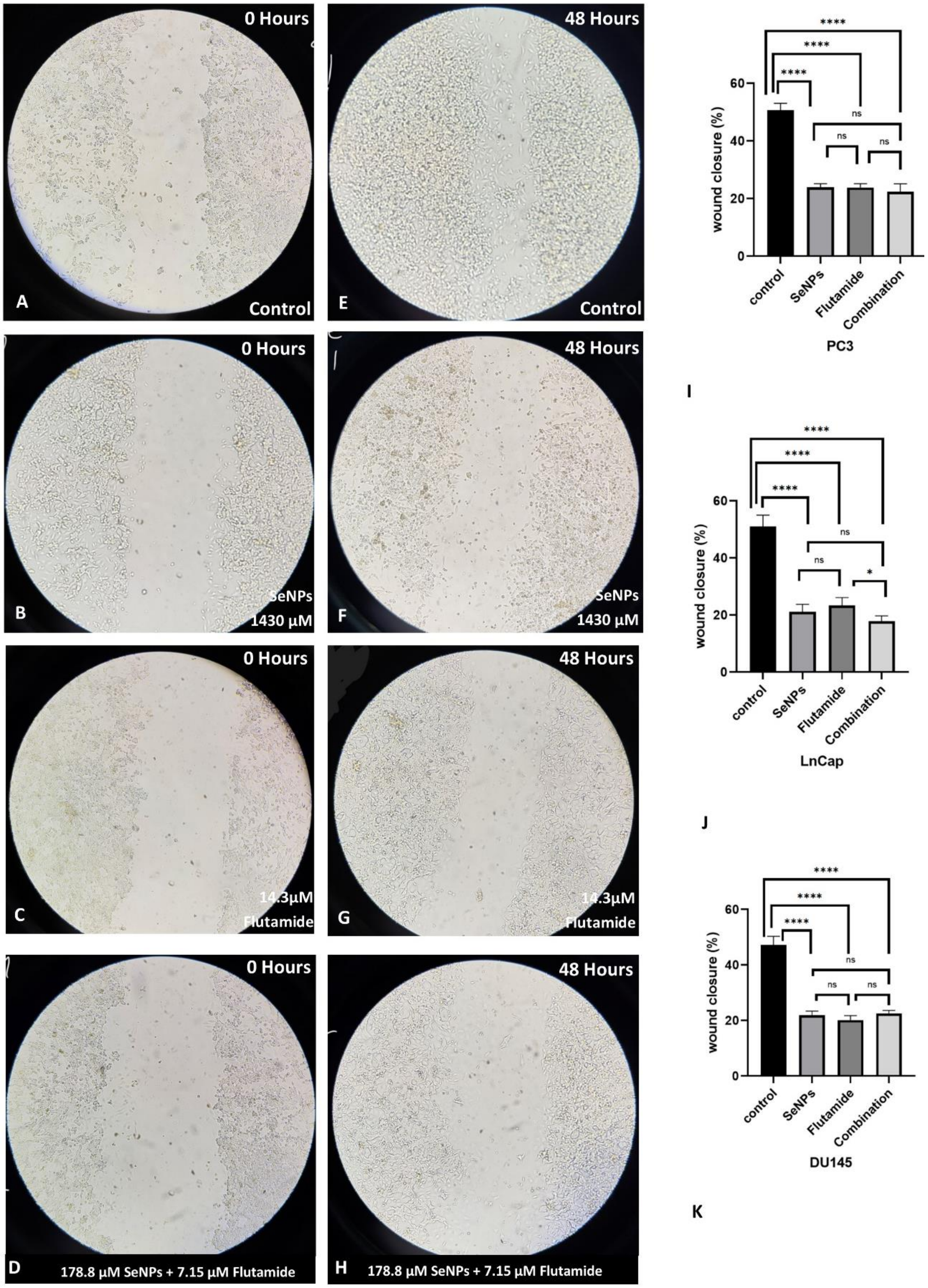


Figure 5. Migration assay for LnCap prostate cancer cells. (A) control-Day 0 (B) SeNPs-Day 0 (C) Flutamide-Day 0 (D) Combination-Day 0 (E) control-Day 2 (F)SeNPs-Day 2 (G)Flutamide-Day 2 (H)combination-Day 2

(I,J,K) all treated group significantly inhibited movements of prostate cancer cell lines into the wound after 48 hours. Statistical significance was defined at *p < 0.05, **p < 0.01, ***p < 0.001, and ****p<0.0001

### *3.6. Decrease in the number of colonies by Flutamide and SeNPs*

There was a clear connection between the metastatic capability of the prostate cell lines and the establishment of colonies by untreated prostate cancer cells. The untreated PC3 cells exhibited a higher number of colonies compared to the LNCap and DU145 cell lines. Both SeNPs and Flutamide, when used at IC50 dosages, effectively suppressed the colony-establishing capability of all the cell lines, in comparison to the unattended cell lines. Additionally, a combination of the two compounds effectively halted the growth of colonies in all administered cell lines. (figure 6)

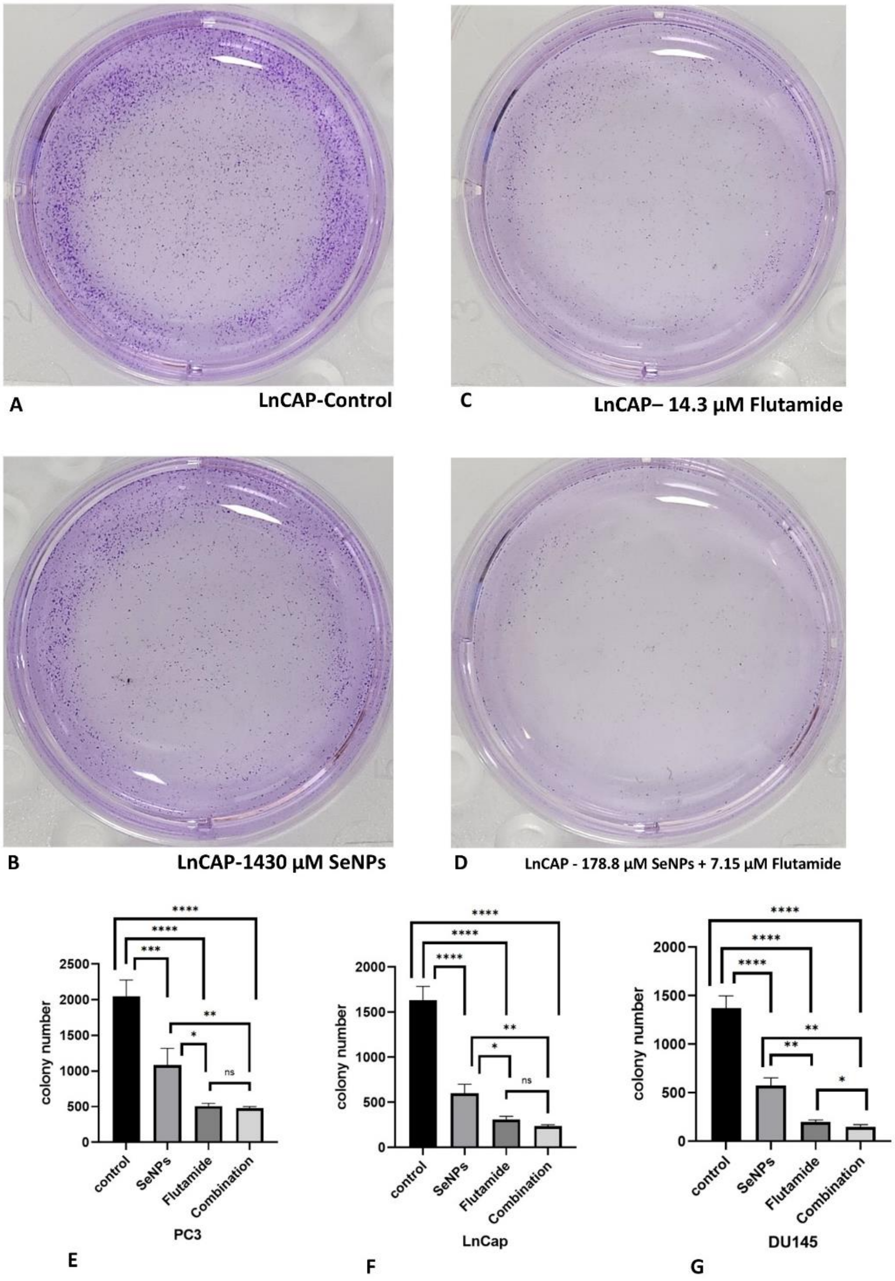

Figure 6. Colony formation assay in LNCaP prostate cancer cells. (A) Control (B) SeNPs (C) Flutamide (D) combination. (E-G) SeNPs, Flutamide, and their combinations in IC50 concentrations prompted a marked reduction in colony count across each one of the stated prostate cancer cell lines. Statistical significance was defined at $^{*}p < 0.05$, $^{**}p < 0.01$, $^{***}p < 0.001$, and $^{****}p<0.0001$.

### *3.7. SeNPs and Flutamide destroy tumor spheroid*

Three-dimensional in vitro simulations exhibit certain aspects of the structure of human tumors and demonstrate resistance to anti-cancer medications (23)(24). Therefore, we assessed the efficacy of SeNPs, Flutamide, and their combinations in a three-dimensional tumor spheroid model. PC3, LNCaP, and DU145 spheroids were cultured and allowed to grow until they reached a diameter of 300-400 µm. Spheroids that received IC50 doses of SeNPs, Flutamide, and their combinations began to shrink and release dead cells after 48 hours of exposure, while the untreated spheroids kept growing. The spheroids that underwent treatment exhibited substantial structural impairment and included a numerous deceased cells. In contrast, the spheroids that received no therapy maintained their normal and undamaged shape (25). (Figure 7)

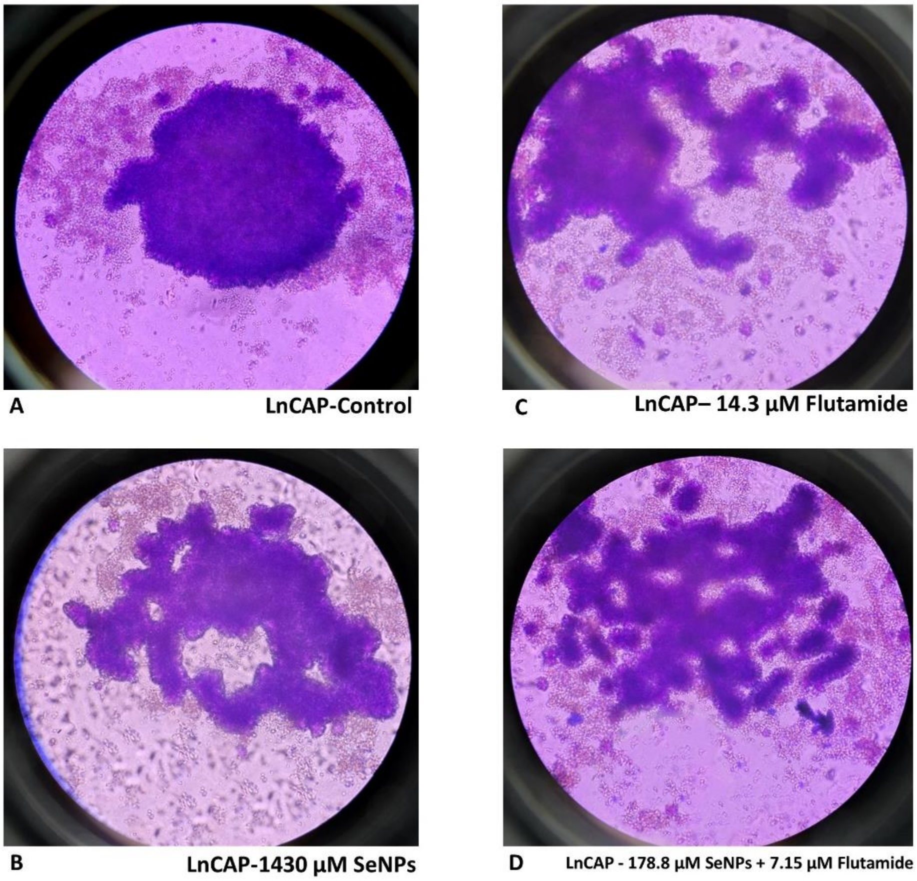


Figure 7. LnCAP tumor spheroid model. Treated spheroids showed notable structural damage and a high presence of dead cells. In contrast, the untreated spheroids maintained a typical intact structure. (A) control (B) SeNPs (C) Flutamide (D) combination

### *3.8. Induction of apoptosis by SeNPs and Flutamide*

In order to ascertain the potential of SeNPs and Flutamide to induce apoptosis in prostate cancer cell lines, Annexin-V/PI staining was performed. Using flow cytometry, the apoptotic effects of SeNPs, Flutamide, and their combinations were analyzed. Without any treatment, the percentage of initial and final apoptotic cells in the PC3 cell line was 2.09% and 9.18%, respectively. For the LnCAP cell line, these values were 2.72% and 13.7%, and for the DU145 cell line, they were 2.03% and 16.4%. However, when treated with SeNPs, these percentages increased to 3.37% and 51.8% for PC3 cells, 3.6% and 36.7% for LnCAP cells, and 2.34% and 45.7% for DU145 cells. The percentage of apoptotic cells after treatment with Flutamide was 6.59% and 33.5% for PC3 cells, 3.18% and 45.5% for LnCAP cells, and 5.28% and 49.3% for DU145 cells. The apoptosis percentages after treatment with the IC50 value of the combination of SeNPs and Flutamide were similar. Specifically, the percentage of apoptotic cells was 3.89% and 40.4% for PC3 cells, 2.49% and 34.7% for LnCAP cells, and 3.28% and 42.7% for DU145 cells. In the PC3 and LnCAP cell lines, the percentage of necrotic cells was greater in the group of cells that had been administered flutamide. Finally, the extremely small percentage of necrotic cells in SeNPs treated cells suggests that this medication is likely safe for use (Figure 8.)

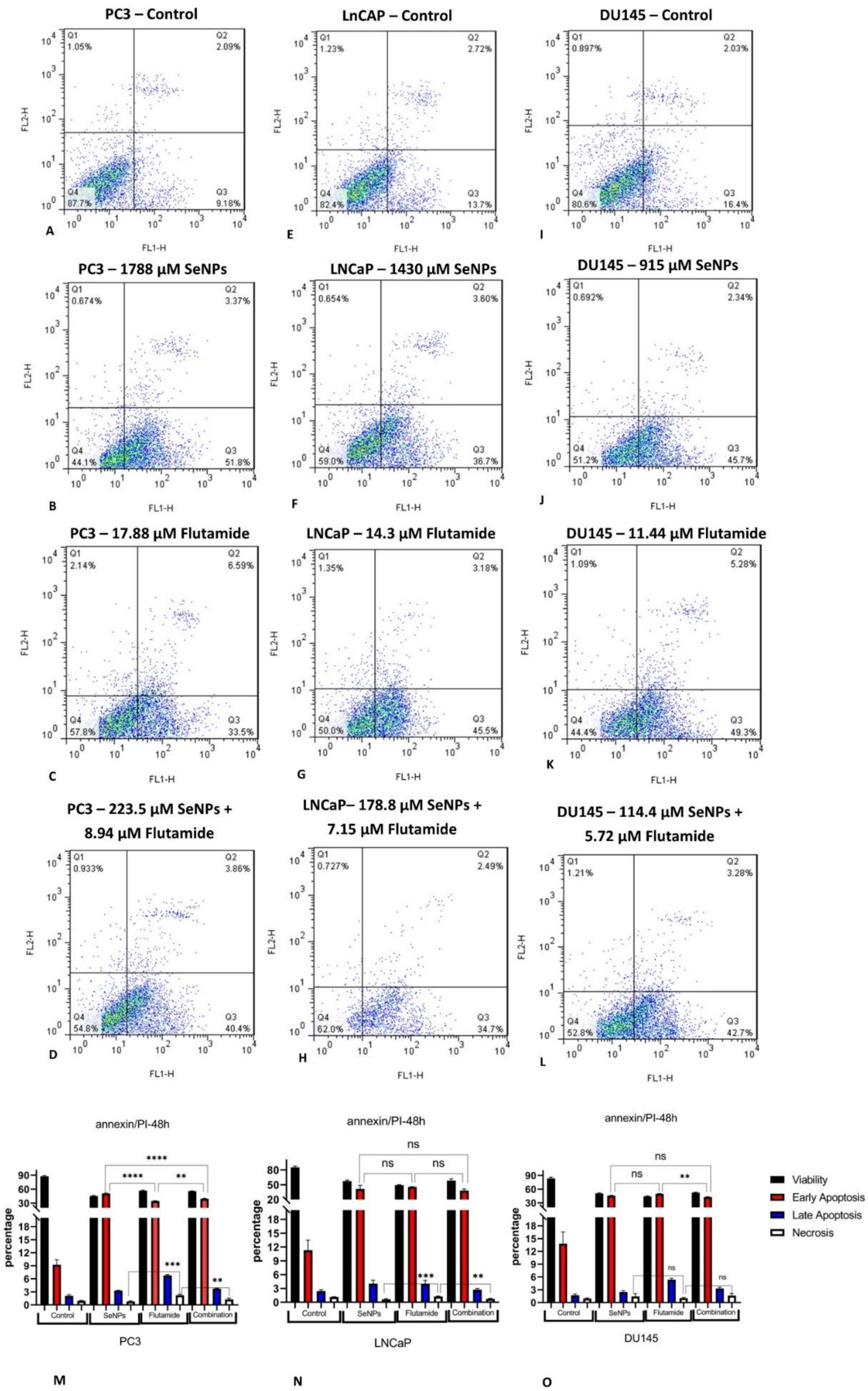

PC3 – Control
Q1 1.05%
Q2 2.09%
Q4 87.7%
Q3 9.18%
A
LnCAP – Control
Q1 1.23%
Q2 2.72%
Q4 82.4%
Q3 13.7%
E
DU145 – Control
Q1 0.897%
Q2 2.03%
Q4 80.6%
Q3 16.4%
I
PC3 – 1788 µM SeNPs
Q1 0.674%
Q2 3.37%
Q4 44.1%
Q3 51.8%
B
LNCaP – 1430 µM SeNPs
Q1 0.654%
Q2 3.60%
Q4 59.0%
Q3 36.7%
F
DU145 – 915 µM SeNPs
Q1 0.692%
Q2 2.34%
Q4 51.2%
Q3 45.7%
J
PC3 – 17.88 µM Flutamide
Q1 2.14%
Q2 6.59%
Q4 57.8%
Q3 33.5%
C
LNCaP – 14.3 µM Flutamide
Q1 1.35%
Q2 3.18%
Q4 50.0%
Q3 45.5%
G
DU145 – 11.44 µM Flutamide
Q1 1.09%
Q2 5.28%
Q4 44.4%
Q3 49.3%
K
PC3 – 223.5 µM SeNPs + 8.94 µM Flutamide
Q1 0.933%
Q2 3.86%
Q4 54.8%
Q3 40.4%
D
LNCaP– 178.8 µM SeNPs + 7.15 µM Flutamide
Q1 0.727%
Q2 2.49%
Q4 62.0%
Q3 34.7%
H
DU145 – 114.4 µM SeNPs + 5.72 µM Flutamide
Q1 1.21%
Q2 3.28%
Q4 52.8%
Q3 42.7%
L
FL1-H
FL2-H
annexin/PI-48h
percentage
Control
SeNPs
Flutamide
Combination
PC3
LNCaP
DU145
Viability
Early Apoptosis
Late Apoptosis
Necrosis
M
N
O

Figure 8. Flow cytometric examination of PC3, LnCAP, and DU145 cell apoptosis using Annexin-V-Flous after 48 hours. (A-D) Induced apoptosis of PC3 cell untreated and treatment with SeNPs, Flutamide, and their combination (In order from up to down). (E-H) Induced apoptosis of LNCaP cell untreated and treatment with SeNPs, Flutamide, and their combination (In order from up to down). (I-L) Induced apoptosis of DU145 cell untreated and treatment with SeNPs, Flutamide, and their combination (In order from up to down). (M-O) there was a significant enhancement in apoptosis of all treated groups compared to the control group in PC3, LnCAP, and DU145 cell lines. Statistical significance was defined at $^{*}p < 0.05$, $^{**}p < 0.01$, $^{***}p < 0.001$, and $^{****}p<0.0001$.

### *3.9. SeNPs and Flutamide induce SubG1-G1 arrest in the prostate cancer cell lines*

The flow cytometric assay of the prostate cancer cell lines PC3, LNCaP, and Du145 subjected to SeNPs, Flutamide, and their combinations in relation to the induction of cell cycle arrest is illustrated in Figure 9. Cells in the phases sub-G1, G1, S, and G2/M phases accounted for 7.31%, 62.9%, 24.5%, and 6.21% of the total cell sample in the control group of PC3 metastatic prostate cancer cells.

When PC3 metastatic prostate cancer cells were treated with SeNPs, there was an increase in sub-G1 cells (7.31% to 80.3%, p<0.0001 )and a decrease in G1 (62.9% to 0.8%, p<0.0001), S (24.5% to 15.6%) and G2/M phase (3.92% to 3.46%) cells. Treatment with Flutamide also caused an increase in sub-G1 cells (7.31% to 69.2%, p<0.0001), and S (24.5% to 27.3%) cells, while decreasing G1 (62.9% to 1.06%, p<0.0001), and G2/M (3.92% to 2.13%) phases. The combination therapy of Flutamide plus SeNPs resulted in a significant increase in sub-G1 cells (7.31% to 91.0%, p<0.0001) for the PC3 cell line. Similar results were observed for LNCaP prostate cancer cells, with an increase in sub-G1 phase cells following treatment with SeNPs (31.1% to 75.9%, p<0.0001), Flutamide (31.1% to 90.9%, p<0.0001), and their combination (31.1% to 46.2%, p<0.01).

In summary, the outcome of the current study indicate that the administration of SeNPs, Flutamide, and their combination effectively halted the progression of the cell cycle in the SubG1/G1 stage, compared to the control and untreated subgroups. Furthermore, the presence of the sub-G1 peak in the flow cytometry graphs suggests apoptosis in the cancer cells (Figure 9.)

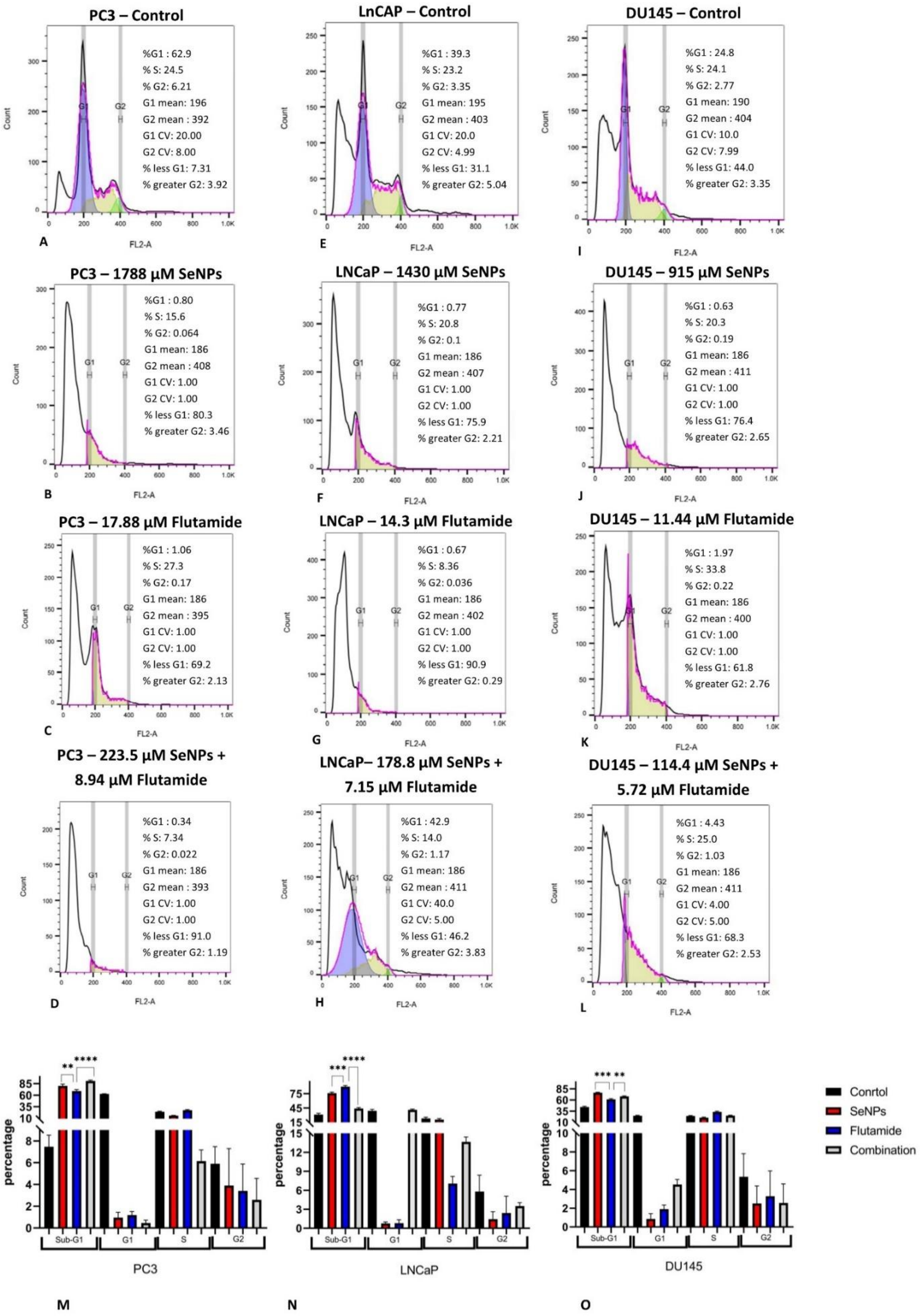
PC3 – Control
%G1 : 62.9
% S: 24.5
% G2: 6.21
G1 mean: 196
G2 mean : 392
G1 CV: 20.00
G2 CV: 8.00
% less G1: 7.31
% greater G2: 3.92
A
LnCAP – Control
%G1 : 39.3
% S: 23.2
% G2: 3.35
G1 mean: 195
G2 mean : 403
G1 CV: 20.0
G2 CV: 4.99
% less G1: 31.1
% greater G2: 5.04
E
DU145 – Control
%G1 : 24.8
% S: 24.1
% G2: 2.77
G1 mean: 190
G2 mean : 404
G1 CV: 10.0
G2 CV: 7.99
% less G1: 44.0
% greater G2: 3.35
I
PC3 – 1788 µM SeNPs
%G1 : 0.80
% S: 15.6
% G2: 0.064
G1 mean: 186
G2 mean : 408
G1 CV: 1.00
G2 CV: 1.00
% less G1: 80.3
% greater G2: 3.46
B
LNCaP – 1430 µM SeNPs
%G1 : 0.77
% S: 20.8
% G2: 0.1
G1 mean: 186
G2 mean : 407
G1 CV: 1.00
G2 CV: 1.00
% less G1: 75.9
% greater G2: 2.21
F
DU145 – 915 µM SeNPs
%G1 : 0.63
% S: 20.3
% G2: 0.19
G1 mean: 186
G2 mean : 411
G1 CV: 1.00
G2 CV: 1.00
% less G1: 76.4
% greater G2: 2.65
J
PC3 – 17.88 µM Flutamide
%G1 : 1.06
% S: 27.3
% G2: 0.17
G1 mean: 186
G2 mean : 395
G1 CV: 1.00
G2 CV: 1.00
% less G1: 69.2
% greater G2: 2.13
C
LNCaP – 14.3 µM Flutamide
%G1 : 0.67
% S: 8.36
% G2: 0.036
G1 mean: 186
G2 mean : 402
G1 CV: 1.00
G2 CV: 1.00
% less G1: 90.9
% greater G2: 0.29
G
DU145 – 11.44 µM Flutamide
%G1 : 1.97
% S: 33.8
% G2: 0.22
G1 mean: 186
G2 mean : 400
G1 CV: 1.00
G2 CV: 1.00
% less G1: 61.8
% greater G2: 2.76
K
PC3 – 223.5 µM SeNPs + 8.94 µM Flutamide
%G1 : 0.34
% S: 7.34
% G2: 0.022
G1 mean: 186
G2 mean : 393
G1 CV: 1.00
G2 CV: 1.00
% less G1: 91.0
% greater G2: 1.19
D
LNCaP– 178.8 µM SeNPs + 7.15 µM Flutamide
%G1 : 42.9
% S: 14.0
% G2: 1.17
G1 mean: 186
G2 mean : 411
G1 CV: 40.0
G2 CV: 5.00
% less G1: 46.2
% greater G2: 3.83
H
DU145 – 114.4 µM SeNPs + 5.72 µM Flutamide
%G1 : 4.43
% S: 25.0
% G2: 1.03
G1 mean: 186
G2 mean : 411
G1 CV: 4.00
G2 CV: 5.00
% less G1: 68.3
% greater G2: 2.53
L
Count
FL2-A
G1
G2
percentage
Sub-G1
G1
S
G2
PC3
LNCaP
DU145
Conrtol
SeNPs
Flutamide
Combination
M
N
O

Figure 9. Cell cycle assessment of PC3, LnCAP, and DU145 cell lines. (A-D) Cell cycle analysis of PC3 cell untreated and treatment with SeNPs, Flutamide, and their combination (In order from up to down). (E-H) Cell cycle analysis of LNCaP cell untreated and treatment with SeNPs, Flutamide, and their combination (In order from up to down). (I-L) Cell cycle analysis of DU145 cell untreated and treatment with SeNPs, Flutamide, and their combination (In order from up to down). (M-O) According to the cell cycle analysis, we detected sub-G1 area increased significantly in PC3, LnCAP, and DU145 cells when treated with SeNPs, Flutamide, and their combination. Statistical significance was defined at $*p < 0.05$, $**p < 0.01$, $***p < 0.001$, and $****p<0.0001$.

### *3.10. Effect of Flutamide and SeNPs on gene expression levels in prostate cancer cell line*

All three prostate cancer cell lines underwent treatment with IC50 doses of SeNPs, Flutamide, and their combination for a duration of 48 hours. Following this, the expression levels of apoptosis-related genes (BAX, Bcl-2), an angiogenesis gene (VEGF-C), a gene associated with progression and development (HIF-1α), genes involved in the EMT pathway (Snail and E-Cadherin), and a prostate cancer marker (KLK3) were evaluated using Real-Time PCR. The resulting data was analyzed with the help of GraphPad Prism version 9 software, as shown in Figure 10.

As depicted in Figure 10, in the LnCAP and DU145 cell lines, all treatment groups, whether individual or in combination, led to a significant decrease in the expression of VEGF-C and HIF-1α genes ($p<0.05$). Regarding the EMT pathway genes, each group that received treatment saw a significant increase in E-cadherin expression and a decrease in Snail gene expression ($p<0.05$). Furthermore, all medication groups significantly reduced the expression of the prostate cancer biomarker known as KLK3 ($p<0.05$). Additionally, every group that underwent treatment up-regulated BAX gene expression significantly and down-regulated Bcl-2 gene expression. Thus, it is suggested that SeNPs could act as a promising anti-cancer treatment for LnCAP and DU145 cells through the induction of apoptosis via the mitochondrial pathway.

In the PC3 cell line, all treatment groups, led to a significant decrease in VEGF-C expression ($p<0.05$). Flutamide and the combination of Flutamide with SeNPs significantly decreased HIF-1α gene expression, while SeNPs alone showed a non-significant decrease. In terms of EMT pathway genes, every group that was treated saw a significant increase in E-cadherin expression ($p<0.05$), but the decrease in Snail gene expression was not significant in any treatment groups. All treatment groups reduced the expression of the prostate cancer biomarker KLK3, with only the Flutamide group showing a significant change ($p<0.05$). Moreover, all treated groups significantly enhanced BAX gene expression and decreased Bcl-2 gene expression. By inducing apoptosis via the mitochondrial route, SeNPs could consequently function as a prospective anti-cancer agent toward PC3 cells.

These results suggest that the IC50 concentration of SeNPs and Flutamide combination (223.5 µM SeNPs + 8.94µM Flutamide for PC3, 178.8 µM SeNPs + 7.15 µM Flutamide for LnCAP, and 114.4 µM SeNPs + 5.72 µM Flutamide for DU145) used in this Real-Time PCR test had similar effects to the IC50 concentration of Flutamide alone (17.88 µM, 14.3 µM, and 11.44 µM for PC3, LnCAP, and DU145 cell lines, respectively). The changes in gene expression mentioned were more significant in LnCAP than in DU145 cell lines, and in DU145 more than in PC3 cell lines.

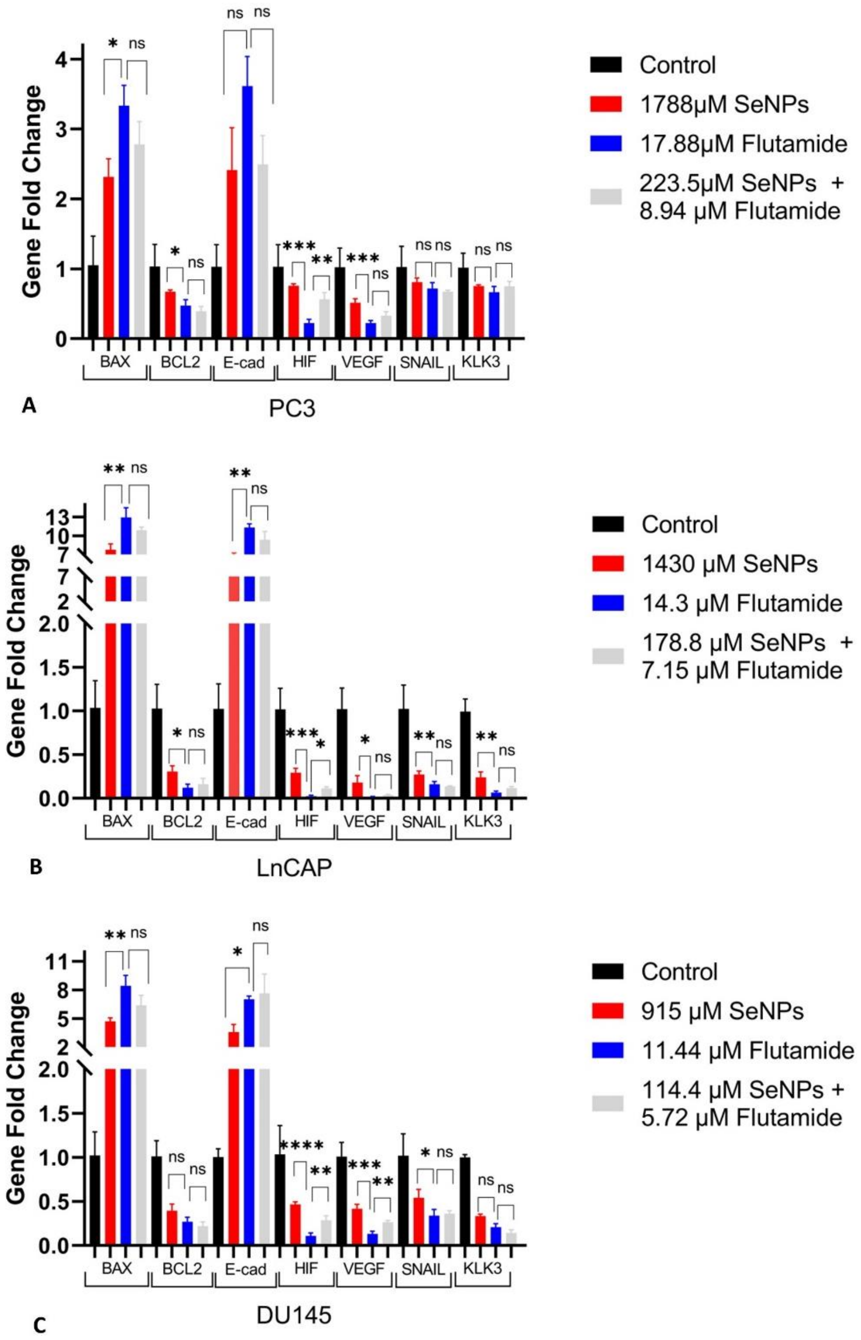


Figure 10. The gene expression rate results of three prostate cancer cell lines (PC3 (A), LnCAP (B), DU145 (C)) treated with SeNPs, Flutamide, their combination on the expression of target genes for 48 h. Values are given as mean ± SD of three independent experiments. Statistical significance was defined at *$p < 0.05$, **$p < 0.01$, ***$p < 0.001$, and ****$p<0.0001$.

## 4. Discussion

When it comes to treating developed and metastatic prostate cancer, hormonal treatment remains the main curative option (26); However, certain individuals develop resistance to ADT and progress to CRPC,

with only a small percentage of them showing a long-term response (27). Drug resistance, expenses, inaccessibility, and serious negative consequences such as hepatotoxicity restrict their use in clinical settings. For instance, flutamide is known to induce liver damage (18). Due to unsatisfactory monotherapy outcomes in CRPC clients, there has been a significant focus on conjunction treatment, which has demonstrated greater efficacy. Numerous investigations have shown the advantages of combining chemotherapy with selenium, among them using Selenocystine with 5-fluorouracil, auranofin with doxorubicin, and Methylseleninic acid with cisplatin. (28-31).

The combination of flutamide and SeNPs for the therapy of metastatic PCa has not been evaluated in any study to date. Our findings show that there is a synergistic reduction in LnCAP, PC3, and DU145 cell survival when flutamide is combined with SeNPs. This combination also promotes apoptosis and inhibits cell growth by inducing cell cycle arrest. The effect of the IC50 concentration of SeNPs and Flutamide combination (178.8 µM SeNPs + 7.15 µM Flutamide ) on early apoptosis of LnCAP cells was similar to the IC50 concentration of flutamide (14.3 µM, $p>0.05$). Additionally, we observed a decrease in the expression of KLK3, angiogenesis and proliferation genes (VEGF-C and HIF-1α), the apoptosis gene (Bcl-2), and Snail in all cell lines treated with SeNPs, Flutamide, or both compared to the control group. On the other hand, the expression of E-cadherin and BAX genes increased. The expression of the mentioned genes (except HIF-1α) in the combination group and flutamide group had no significant difference ($p>0.05$) in the LnCAP cell line. Therefore, SeNPs have the potential to be highly beneficial in treating metastatic PCa and should be considered as a powerful therapy option for this condition, taking into account both its advantages and negative effects. Combining flutamide with SeNPs may reduce the required intake of flutamide and mitigate its negative effects, particularly its hepatotoxicity.

Multiple non-randomized tests have suggested that flutamide could be an additional treatment option in hormone therapy (32-34). However, the precise mechanisms responsible for the anti-tumor effects of flutamide are not yet fully understood. Shen and colleagues discovered that flutamide enhances cell death and inhibits cell growth, possibly through the upregulation of KLF9(35). Consistent with our findings, Canine KLK2 values fell in dogs that have been administered flutamide, as reported by Mitchell G. Lawrence et al(36).

Inorganic, organic, and SeNPs are only a few of the many selenium classes that are now accessible for employing in anti-cancer protocols. Various forms of selenium compounds have distinct biological effects due to their differing physical characteristics, chemical compositions, metabolic pathways in cells and tissues, and the range of molecules they impact within cells. SeNPs are nanoparticles that provide distinct benefits such as minimal toxicity and rapid uptake by cells compared to other forms of selenium (37, 38). SeNPs have gained attention for their superior anti-cancer properties and lower toxicity in contrast to other inorganic and organic selenium compounds, making them a promising candidate for cancer prevention (39-42). The anticancer action of selenium is believed to be attributed to various mechanisms including cell cycle arrest, antioxidation, apoptosis, and disruption of cell signaling processes (6, 7, 15, 43-45).

Sonkusre and colleagues demonstrated that SeNPs at a dosage of 2 µg Se/ml induced necroptosis in PC-3 cells through internalization, driven by reactive oxygen species (ROS). The real-time qPCR analysis indicated an upregulation in the expression of necroptosis-associated tumor necrotic factor (TNF) and interferon regulatory factor 1 (IRF1). SeNPs administration led to higher RIP1 protein expression at the translational phase. Additionally, cell survival was notably enhanced when treated with the necroptosis

inhibitor, Necrostatin-1 (46). Biogenic selenium nanoparticles produced from Bacillus licheniformis may cause necroptosis in LNCaP-FGC cells at a dosage of 2 µg Se/ml, without impacting the integrity of red blood cells. Real-time gene expression estimation showed increased levels of TNF and IRF1, and reduced levels of androgen receptor (AR) and PSA (47). The dosage of SeNPs used in our study was higher than in the studies mentioned. Additionally, levels of the androgen receptor were not evaluated in our study.

Yulin An alongside his team explored the therapeutic effects of lentinan-functionalized selenium nanoparticles (LET-SeNPs) combined with zoledronic acid (ZOL) on metastatic PCa cells. The study demonstrated that this combination could significantly reduce the growth of PCa cells in a concentration-dependent manner, as shown through cytotoxicity assays, flow cytometry, and tests on mitochondrial functions. The enhanced anticancer properties of LET-SeNPs with ZOL were linked to the modulation of BCL2 family proteins, which promotes the release of cytochrome C from the mitochondria into the cytoplasm. This action triggers cell cycle arrest in the S phase, leading to irreversible DNA damage and the eradication of PCa cells. Consequently, the research highlights that SeNPs and ZOL together offer a potent solution for curtailing the expansion of PCa cells(48).

There is a limited number of animal research studies investigating the anticancer effects of SeNPs. Sonkusre's histopathology research demonstrated that ingesting selenium nanoparticles at a concentration of 50 mg Se/kg of body weight resulted in significantly decreased toxicity compared to L-selenomethionine (5 mg Se/kg)(47). Furthermore, Shahverdi et al. discovered that folic acid surface-coated selenium nanoparticles exhibited in vivo benefits for breast cancer. They found that SeNPs could inhibit PCa growth by reducing IKK-ε and SMAD2 mRNA levels in xenograft mice, suggesting that SeNPs could be used for anti-tumor purposes. The effectiveness of SeNPs dropped notably in mice treated with miR-155-5p shRNA, confirming the crucial role of miR-155-5p in facilitating SeNPs' anti-tumor properties in living creatures(49).

The precise biochemical process that enables SeNPs to trigger tumor inhibition remains incompletely understood. It is widely accepted that SeNPs can induce tumor cell death by increasing cellular absorption and inhibiting ROS(50). Huang and colleagues have shown that selenium nanoparticles may induce autophagy in cancer cells, therefore exhibiting an anticancer effect (51). Sonkusre's findings revealed that SeNPs led to an increase in TNF, ultimately resulting in the activation of cancer cell necrosis (46). Vekariya et al. found that SeNPs impeded cell development and the synthesis of DNA, RNA, and proteins, indicating that SeNPs could impact the regulation of various relevant molecules, including non-coding RNA(52).

In order to assess the potential pathway of apoptosis triggered by SeNPs, we examined the expression of the apoptotic gene BAX and the anti-apoptotic gene Bcl-2. The ratio of these genes was considered indicative of the drug's impact on mitochondrial apoptosis. After the administration of SeNPs, Flutamide, or a combination of both, there was a decrease in the expression of the anti-apoptotic Bcl-2 gene and an increase in the expression of the pro-apoptotic BAX gene. This shift in gene expression led to increased apoptosis, which was linked to the expression of the cyclin-related inhibitor p21 (53). Bcl-2 family proteins play a role in regulating programmed cell death by influencing mitochondrial function (54). Indeed, depending on many physiological and pathological conditions, alterations in the electrical potential of mitochondria may result in a discharge of apoptotic proteins, including pro-caspases 2, 3, and 9.(55)

It is hypothesized that the production of ROS is essential for mitochondrial oxidative damage, which triggers apoptosis (56). By reducing cellular ROS, Bcl-2 performs an anti-apoptotic action in this way (55). As a consequence, intracellular ROS buildup that leads to mitochondrial-mediated apoptosis is favored by

downregulating Bcl-2 and overexpressing BAX. Nevertheless, the P53 protein, a tumor suppressor that triggers cell cycle arrest and death in response to damage to the genome, has two well-known transcriptional targets: BAX and Bcl-2 (57). Taken together, The enhanced expression of BAX and diminished expression of Bcl-2 support apoptosis and reduced growth of PCa cells.

We investigated two crucial regulators of angiogenesis, VEGF-C, and HIF-1α, along with genes related to apoptosis. VEGF-C is a powerful pro-angiogenic agent that plays a crucial role in microvascular restructuring, angiogenesis, and the advancement of PCa spread and progression(58). VEGF-C expression has been linked to the advancement of prostate cancer and lymph node metastasis, according to studies done by Yang and Jenbacken (59, 60). Our findings suggest that the androgen communication channel may be involved in the SeNPs-induced changes in the expression of VEGF-C and HIF-1α genes. Treating androgen-dependent LNCaP cells with SeNPs resulted in a significant drop in VEGF-C and HIF-1α expression. In androgen-independent PC-3 cells, SeNPs treatment also reduced the expressions of VEGF-C and HIF-1α, but to a lesser extent compared to LnCAP cells. These results suggest that SeNPs may be beneficial for prostate cancer treatment, particularly in the early stages. Further extensive studies is required to validate and support our findings.

Recent investigations have shown that human kallikrein-related peptidase 3 (KLK3), a protease found exclusively in the prostate, can promote the advancement of prostate cancer and serve as a biomarker (61, 62). Studies have shown that increased expression of the KLK3 gene in prostate cancer is linked to several processes such as cell growth, movement, infiltration, blood vessel formation, and resistance to cell death(63, 64). KLK3 has been utilized in several research studies as a biological indicator for prostate cancer prognosis and as a target for therapy. Treatment with SeNPs, Flutamide, or a combination of them significantly reduced KLK3 mRNA expression in all prostate cancer cell lines, confirming the tumor-inhibiting properties of these chemicals.

We suggest that future studies delve deeper into specific issues. They could focus on studying the impact of SeNPs on cancer stem cells, which play a crucial role in the recurrence of the disease due to their resistant characteristics. Additionally, our findings can be validated through knockdown/overexpression studies employing RNAi and viral-based gene therapies. Subsequent research could involve using xenograft murine models to evaluate the adaptive response of prostate cancer cells to SeNPs in vivo. The Western blot technique may be utilized to examine the protein expression of VEGF-C, KLK2, and the EMT system.

## 5. Conclusion

We have demonstrated for the first time that the combination therapy of flutamide and SeNPs enhances the anti-tumor effect on PCa cell lines, especially in LnCAP cells. This is due to their ability to induce apoptosis and prevent the growth of PCa cells. It is suggested that the concurrent administration of SeNPs and flutamide could potentially reduce the effective dosage of flutamide and mitigate its adverse effects, particularly hepatotoxicity.

## Authors' contributions

**Iman Menbari Oskouie**: writing original draft; **Fatemeh Khatami**: Investigation; **Amin Shiralizadeh Dezfuli**: Validation; **Rahil Mashhadi**: Validation; **Akram Mirzaei**: methodology; **Seyedeh Negin Hashemi Dorgaheh**: methodology; **Helia Azodian Ghajar**: methodology; Ramin **Heshmat**: data analysis **Seyed Mohammad Kazem Aghamir**: conceptualization

## Ethical approval

The study was conducted at the Urology Research Center, Tehran University of Medical Science. subsequent to obtaining REB authorization from the Ethics Review Board of Sina Hospital.

## Consent to participate

Not applicable.

## Consent to publish

Not applicable.


## Funding

No funding.


## Availability and data and materials

Data will be provided on request.

## Conflict of interest

There is no conflict of interest.

## References

1. Chen J, Zhang D, Yan W, Yang D, Shen B. Translational bioinformatics for diagnostic and prognostic prediction of prostate cancer in the next-generation sequencing era. BioMed research international. 2013;2013.
2. Hjelmborg JB, Scheike T, Holst K, Skytthe A, Penney KL, Graff RE, et al. The heritability of prostate cancer in the Nordic Twin Study of Cancer. Cancer Epidemiology, Biomarkers & Prevention. 2014;23(11):2303-10.
3. Bach C, Pisipati S, Daneshwar D, Wright M, Rowe E, Gillatt D, et al. The status of surgery in the management of high-risk prostate cancer. Nature Reviews Urology. 2014;11(6):342-51.
4. Brogden RN, Chrisp P. Flutamide: A review of its pharmacodynamic and pharmacokinetic properties, and therapeutic use in advanced prostatic cancer. Drugs & aging. 1991;1:104-15.
5. Goldspiel BR, Kohler DR. Flutamide: an antiandrogen for advanced prostate cancer. Dicp. 1990;24(6):616-23.
6. Chen P, Wang L, Li N, Liu Q, Ni J. Comparative proteomics analysis of sodium selenite-induced apoptosis in human prostate cancer cells. Metallomics. 2013;5(5):541-50.
7. Xiang N, Zhao R, Zhong W. Sodium selenite induces apoptosis by generation of superoxide via the mitochondrial-dependent pathway in human prostate cancer cells. Cancer chemotherapy and pharmacology. 2009;63(2):351-62.
8. Liu T, Lai L, Song Z, Chen T. A sequentially triggered nanosystem for precise drug delivery and simultaneous inhibition of cancer growth, migration, and invasion. Advanced Functional Materials. 2016;26(43):7775-90.
9. Zhang Z, Du Y, Liu T, Wong K-H, Chen T. Systematic acute and subchronic toxicity evaluation of polysaccharide–protein complex-functionalized selenium nanoparticles with anticancer potency. Biomaterials science. 2019;7(12):5112-23.
10. Yang Y, Xie Q, Zhao Z, He L, Chan L, Liu Y, et al. Functionalized selenium nanosystem as radiation sensitizer of 125I seeds for precise cancer therapy. ACS Applied Materials & Interfaces. 2017;9(31):25857-69.
11. Huang J, Huang W, Zhang Z, Lin X, Lin H, Peng L, et al. Highly uniform synthesis of selenium nanoparticles with EGFR targeting and tumor microenvironment-responsive ability for simultaneous diagnosis and therapy of nasopharyngeal carcinoma. ACS applied materials & interfaces. 2019;11(12):11177-93.
12. Li T, Pan S, Gao S, Xiang W, Sun C, Cao W, et al. Diselenide–Pemetrexed assemblies for combined cancer Immuno-, radio-, and chemotherapies. Angewandte Chemie International Edition. 2020;59(7):2700-4.
13. Liu T, Xu L, He L, Zhao J, Zhang Z, Chen Q, et al. Selenium nanoparticles regulates selenoprotein to boost cytokine-induced killer cells-based cancer immunotherapy. Nano Today. 2020;35:100975.
14. Gao S, Li T, Guo Y, Sun C, Xianyu B, Xu H. Selenium-containing nanoparticles combine the NK cells mediated immunotherapy with radiotherapy and chemotherapy. Advanced Materials. 2020;32(12):1907568.
15. Luo H, Wang F, Bai Y, Chen T, Zheng W. Selenium nanoparticles inhibit the growth of HeLa and MDA-MB-231 cells through induction of S phase arrest. Colloids and Surfaces B: Biointerfaces. 2012;94:304-8.
16. Kong L, Yuan Q, Zhu H, Li Y, Guo Q, Wang Q, et al. The suppression of prostate LNCaP cancer cells growth by Selenium nanoparticles through Akt/Mdm2/AR controlled apoptosis. Biomaterials. 2011;32(27):6515-22.
17. Termini D, Den Hartogh DJ, Jaglanian A, Tsiani E. Curcumin against prostate cancer: current evidence. Biomolecules. 2020;10(11):1536.

18. Iguchi T, Tamada S, Kato M, Yasuda S, Machida Y, Ohmachi T, et al. Enzalutamide versus flutamide for castration-resistant prostate cancer after combined androgen blockade therapy with bicalutamide: the OCUU-CRPC study. International journal of clinical oncology. 2020;25:486-94.
19. Friedrich J, Seidel C, Ebner R, Kunz-Schughart LA. Spheroid-based drug screen: considerations and practical approach. Nature protocols. 2009;4(3):309-24.
20. Srivastava N, Mukhopadhyay M. Green synthesis and structural characterization of selenium nanoparticles and assessment of their antimicrobial property. Bioprocess and biosystems engineering. 2015;38:1723-30.
21. Kirupagaran R, Saritha A, Bhuvaneswari S. Green synthesis of selenium nanoparticles from leaf and stem extract of Leucas lavandulifolia Sm. and their application. Journal of Nanoscience and Technology. 2016:224-6.
22. Chen Y-t, Zhang W, Fan Y-q, Xu X-q, Zhang Z-x. Hydrothermal preparation of selenium nanorods. Materials chemistry and physics. 2006;98(2-3):191-4.
23. Kim JB, editor Three-dimensional tissue culture models in cancer biology. Seminars in cancer biology; 2005: Elsevier.
24. Takagi A, Watanabe M, Ishii Y, Morita J, Hirokawa Y, Matsuzaki T, et al. Three-dimensional cellular spheroid formation provides human prostate tumor cells with tissue-like features. Anticancer research. 2007;27(1A):45-53.
25. Phung YT, Barbone D, Broaddus VC, Ho M. Rapid generation of in vitro multicellular spheroids for the study of monoclonal antibody therapy. Journal of Cancer. 2011;2:507.
26. Miyake H, Hara I, Eto H. Clinical outcome of maximum androgen blockade using flutamide as second-line hormonal therapy for hormone-refractory prostate cancer. BJU international. 2005;96(6):791-5.
27. Akaza H, Homma Y, Usami M, Hirao Y, Tsushima T, Okada K, et al. Efficacy of primary hormone therapy for localized or locally advanced prostate cancer: results of a 10-year follow-up. BJU international. 2006;98(3):573-9.
28. Fan C, Chen J, Wang Y, Wong Y-S, Zhang Y, Zheng W, et al. Selenocystine potentiates cancer cell apoptosis induced by 5-fluorouracil by triggering reactive oxygen species-mediated DNA damage and inactivation of the ERK pathway. Free Radical Biology and Medicine. 2013;65:305-16.
29. Fan C, Zheng W, Fu X, Li X, Wong Y, Chen T. Enhancement of auranofin-induced lung cancer cell apoptosis by selenocystine, a natural inhibitor of TrxR1 in vitro and in vivo. Cell death & disease. 2014;5(4):e1191-e.
30. Fan C, Zheng W, Fu X, Li X, Wong Y-S, Chen T. Strategy to enhance the therapeutic effect of doxorubicin in human hepatocellular carcinoma by selenocystine, a synergistic agent that regulates the ROS-mediated signaling. Oncotarget. 2014;5(9):2853.
31. Zhang Y, Zheng S, Zheng J-S, Wong K-H, Huang Z, Ngai S-M, et al. Synergistic induction of apoptosis by methylseleninic acid and cisplatin, the role of ROS-ERK/AKT-p53 pathway. Molecular pharmaceutics. 2014;11(4):1282-93.
32. Fujikawa K, Matsui Y, Fukuzawa S, Takeuchi H. Prostate–Specific Antigen Levels and Clinical Response to Flutamide as the Second Hormone Therapy for Hormone–Refractory Prostate Carcinoma. European urology. 2000;37(2):218-22.
33. Nishimura K, Arichi N, Tokugawa S, Yoshioka I, Kishikawa H, Ichikawa Y. Effects of flutamide as a second-line agent for maximum androgen blockade of hormone refractory prostate cancer. International journal of urology. 2007;14(3):264-7.
34. Suzuki H, Okihara K, Miyake H, Fujisawa M, Miyoshi S, Matsumoto T, et al. Alternative nonsteroidal antiandrogen therapy for advanced prostate cancer that relapsed after initial maximum androgen blockade. The Journal of urology. 2008;180(3):921-7.

35. Shen P, Sun J, Xu G, Zhang L, Yang Z, Xia S, et al. KLF9, a transcription factor induced in flutamide-caused cell apoptosis, inhibits AKT activation and suppresses tumor growth of prostate cancer cells. The Prostate. 2014;74(9):946-58.
36. Lawrence MG, Lai J, Clements JA. Kallikreins on steroids: structure, function, and hormonal regulation of prostate-specific antigen and the extended kallikrein locus. Endocrine reviews. 2010;31(4):407-46.
37. Zheng L, Huang X, Lin X, Lin W, Yang F, Chen T. Thermosensitive hydrogels for sustained-release of sorafenib and selenium nanoparticles for localized synergistic chemoradiotherapy. Biomaterials. 2019;216:119220.
38. Liu T, Zeng L, Jiang W, Fu Y, Zheng W, Chen T. Rational design of cancer-targeted selenium nanoparticles to antagonize multidrug resistance in cancer cells. Nanomedicine: Nanotechnology, Biology and Medicine. 2015;11(4):947-58.
39. Zhang J, Wang X, Xu T. Elemental selenium at nano size (Nano-Se) as a potential chemopreventive agent with reduced risk of selenium toxicity: comparison with se-methylselenocysteine in mice. Toxicological sciences. 2008;101(1):22-31.
40. Wang H, Zhang J, Yu H. Elemental selenium at nano size possesses lower toxicity without compromising the fundamental effect on selenoenzymes: comparison with selenomethionine in mice. Free Radical Biology and Medicine. 2007;42(10):1524-33.
41. Zhang J-S, Gao X-Y, Zhang L-D, Bao Y-P. Biological effects of a nano red elemental selenium. Biofactors. 2001;15(1):27-38.
42. Zhang J, Wang H, Yan X, Zhang L. Comparison of short-term toxicity between Nano-Se and selenite in mice. Life sciences. 2005;76(10):1099-109.
43. Huang Y, He L, Liu W, Fan C, Zheng W, Wong Y-S, et al. Selective cellular uptake and induction of apoptosis of cancer-targeted selenium nanoparticles. Biomaterials. 2013;34(29):7106-16.
44. Nie T, Wu H, Wong K-H, Chen T. Facile synthesis of highly uniform selenium nanoparticles using glucose as the reductant and surface decorator to induce cancer cell apoptosis. Journal of Materials Chemistry B. 2016;4(13):2351-8.
45. Dong Y, Lee SO, Zhang H, Marshall J, Gao AC, Ip C. Prostate specific antigen expression is down-regulated by selenium through disruption of androgen receptor signaling. Cancer research. 2004;64(1):19-22.
46. Sonkusre P, Cameotra SS. Biogenic selenium nanoparticles induce ROS-mediated necroptosis in PC-3 cancer cells through TNF activation. Journal of nanobiotechnology. 2017;15(1):1-12.
47. Sonkusre P. Specificity of biogenic selenium nanoparticles for prostate cancer therapy with reduced risk of toxicity: an in vitro and in vivo study. Frontiers in Oncology. 2020;9:1541.
48. An Y, Zhao J. Functionalized selenium nanotherapeutics synergizes with zoledronic acid to suppress prostate cancer cell growth through induction of mitochondria-mediated apoptosis and cell cycle S phase arrest. Frontiers in Oncology. 2021;11:685784.
49. Shahverdi AR, Shahverdi F, Faghfuri E, Mavandadnejad F, Yazdi MH, Amini M. Characterization of folic acid surface-coated selenium nanoparticles and corresponding in vitro and in vivo effects against breast cancer. Archives of Medical Research. 2018;49(1):10-7.
50. Liu C, Fu Y, Li C-e, Chen T, Li X. Phycocyanin-functionalized selenium nanoparticles reverse palmitic acid-induced pancreatic β cell apoptosis by enhancing cellular uptake and blocking reactive oxygen species (ROS)-mediated mitochondria dysfunction. Journal of Agricultural and Food Chemistry. 2017;65(22):4405-13.
51. Huang G, Liu Z, He L, Luk K-H, Cheung S-T, Wong K-H, et al. Autophagy is an important action mode for functionalized selenium nanoparticles to exhibit anti-colorectal cancer activity. Biomaterials science. 2018;6(9):2508-17.

52. Vekariya KK, Kaur J, Tikoo K. ERα signaling imparts chemotherapeutic selectivity to selenium nanoparticles in breast cancer. Nanomedicine: Nanotechnology, Biology and Medicine. 2012;8(7):1125-32.
53. Jadhav V, Ray P, Sachdeva G, Bhatt P. Biocompatible arsenic trioxide nanoparticles induce cell cycle arrest by p21WAF1/CIP1 expression via epigenetic remodeling in LNCaP and PC3 cell lines. Life sciences. 2016;148:41-52.
54. Kitazawa H, Numakawa T, Adachi N, Kumamaru E, Tuerxun T, Kudo M, et al. Cyclophosphamide promotes cell survival via activation of intracellular signaling in cultured cortical neurons. Neuroscience letters. 2010;470(2):139-44.
55. Sun Y, Wang C, Wang L, Dai Z, Yang K. Arsenic trioxide induces apoptosis and the formation of reactive oxygen species in rat glioma cells. Cellular & Molecular Biology Letters. 2018;23(1):1-10.
56. Bauer G. Reactive oxygen and nitrogen species: efficient, selective, and interactive signals during intercellular induction of apoptosis. Anticancer research. 2000;20(6B):4115-39.
57. Adams JM, Cory S. The Bcl-2 apoptotic switch in cancer development and therapy. Oncogene. 2007;26(9):1324-37.
58. Wu TT-L, Wang J-S, Jiann B-P, Yu C-C, Tsai J-Y, Lin J-T, et al. Expression of vascular endothelial growth factor in Taiwanese benign and malignant prostate tissues. Journal of the Chinese Medical Association. 2007;70(9):380-4.
59. Maxwell PJ, McKechnie M, Armstrong CW, Manley JM, Ong CW, Worthington J, et al. Attenuating adaptive VEGF-A and IL8 signaling restores durable tumor control in AR antagonist–treated prostate cancers. Molecular Cancer Research. 2022;20(6):841-53.
60. Yang J, Wu HF, Qian LX, Zhang W, Hua LX, Yu ML, et al. Increased expressions of vascular endothelial growth factor (VEGF), VEGF-C and VEGF receptor-3 in prostate cancer tissue are associated with tumor progression. Asian journal of andrology. 2006;8(2):169-75.
61. Shang Z, Niu Y, Cai Q, Chen J, Tian J, Yeh S, et al. Human kallikrein 2 (KLK2) promotes prostate cancer cell growth via function as a modulator to promote the ARA70-enhanced androgen receptor transactivation. Tumor Biology. 2014;35:1881-90.
62. Diamandis EP, Yousef GM, Luo L-Y, Magklara A, Obiezu CV. The new human kallikrein gene family: implications in carcinogenesis. Trends in Endocrinology & Metabolism. 2000;11(2):54-60.
63. Kyte JA. Strategies for improving the efficacy of CAR T cells in solid cancers. Cancers. 2022;14(3):571.
64. Bonk S, Kluth M, Jansen K, Hube-Magg C, Makrypidi-Fraune G, Höflmayer D, et al. Reduced KLK2 expression is a strong and independent predictor of poor prognosis in ERG-negative prostate cancer. The Prostate. 2020;80(13):1097-107.
